# The Situated Assessment Method (SAM^2^): Establishing individual differences in habitual behavior

**DOI:** 10.1371/journal.pone.0286954

**Published:** 2023-06-22

**Authors:** Léo Dutriaux, Naomi E. Clark, Esther K. Papies, Christoph Scheepers, Lawrence W. Barsalou

**Affiliations:** 1 Laboratoire sur les Interactions Cognition, Action, Émotion (LICAÉ), Université Paris Nanterre, Nanterre Cedex, France; 2 School of Health and Life Sciences, Glasgow Caledonian University, Glasgow, United Kingdom; 3 School of Health & Wellbeing, University of Glasgow, Glasgow, United Kingdom; 4 School of Psychology and Neuroscience, University of Glasgow, Glasgow, United Kingdom; University of New South Wales, AUSTRALIA

## Abstract

From the perspectives of grounded, situated, and embodied cognition, we have developed a new approach for assessing individual differences. Because this approach is grounded in two dimensions of situatedness—situational experience and the Situated Action Cycle—we refer to it as the *Situated Assessment Method* (SAM^2^). Rather than abstracting over situations during assessment of a construct (as in traditional assessment instruments), SAM^2^ assesses a construct in situations where it occurs, simultaneously measuring factors from the Situated Action Cycle known to influence it. To demonstrate this framework, we developed the SAM^2^ Habitual Behavior Instrument (SAM^2^ HBI). Across three studies with a total of 442 participants, the SAM^2^ HBI produced a robust and replicable pattern of results at both the group and individual levels. Trait-level measures of habitual behavior exhibited large reliable individual differences in the regularity of performing positive versus negative habits. Situational assessments established large effects of situations and large situation by individual interactions. Several sources of evidence demonstrated construct and content validity for SAM^2^ measures of habitual behavior. At both the group and individual levels, these measures were associated with factors from the Situated Action Cycle known to influence habitual behavior in the literature (consistency, automaticity, immediate reward, long-term reward). Regressions explained approximately 65% of the variance at the group level and a median of approximately 75% at the individual level. SAM^2^ measures further exhibited well-established interactions with personality measures for self-control and neuroticism. Cognitive-affective processes from the Situated Action Cycle explained nearly all the variance in these interactions. Finally, a composite measure of habitualness established habitual behaviors at both the group and individual levels. Additionally, a composite measure of reward was positively related to the composite measure of habitualness, increasing with self-control and decreasing with neuroticism.

## Introduction

From the perspectives of grounded, situated, and embodied cognition, the processes that implement human cognition are not abstract, amodal, and decontextualized but instead incorporate situations, embodiment, and action intrinsically into their operation. Decontextualizing cognition by abstracting over situations, embodiment, and action ignores fundamental processes and produces distorted accounts. Decades of research have established the grounded, situated, and embodied perspectives theoretically and empirically [1–10].

Inspired by these perspectives, we have developed a new approach for assessing individual differences. Because this approach is grounded in two dimensions of situatedness—situational experience and the Situated Action Cycle—we refer to it as the *Situated Assessment Method* (SAM^2^). In the remainder of the introduction, we review properties of traditional assessment instruments. We then use these properties to motivate the SAM^2^ framework. Finally, we present a specific implementation of this framework: the SAM^2^ Habitual Behavior Instrument (SAM^2^ HBI).

### Traditional assessment instruments

From the perspective of situated cognition, it is striking how disconnected traditional assessment instruments often are from real-life situations. Typically, assessment instruments use *decontextualized* items to assess an individual difference of interest. To illustrate, consider an item that assesses conscientiousness in the classic Five Factor Model: “I am someone who perseveres until a task is finished” [11–14]. In responding to this item, individuals must abstract over situations to establish a general assessment of how much they agree with it. Similarly consider an item from the Self-Control Scale, “I am good at resisting temptation” [15]. Again, individuals must abstract over situations to establish a general assessment. Many other assessment instruments similarly ask individuals to respond generally across situations to decontextualized items, including the Perceived Stress Scale [16], the Positive and Negative Affect Schedule [17], the Life Satisfaction Scale [18], the Three-Factor Eating Questionnaire [19], and the Five-Facet Mindfulness Questionnaire [20].

#### Instrument construction

To develop traditional instruments like these that assess a construct across individuals, an initial pool of decontextualized items related to the construct is developed. To assess neuroticism, for example, items related to emotional reactivity, worry, and moodiness might be sampled. Items can be generated in a bottom up manner from lexical sources such as dictionaries [13] or from preliminary qualitative research [21, 22]. Alternatively, items can be generated in a top-down manner via literature review and expert intuition [12].

To identify a subset of items that coherently measures the target construct, exploratory factor analysis and other methods establish the latent factor structure of the item pool. Items that only load highly on the critical construct are then extracted from the pool and assessed again in a preliminary test instrument. Confirmatory factor analysis and other methods verify that all final test items measure the same target construct well (i.e., high test coherence).

By aggregating scores across these items into an average or sum, an overall measure for the test is established that orders individuals from high to low on the construct (e.g., high to low neuroticism). Ideally, the aggregate measure should order individuals reliably—it must exhibit satisfactory *test reliability*. As assessed by Cronbach’s alpha and related statistics [23–27], an aggregate measure of a presumably stable trait such as neuroticism should exhibit test reliability of at least .7 to .8. Intuitively, alpha estimates how highly aggregate scores for individuals on one occasion would correlate with scores from the same test administered to the same individuals again under ideal conditions (e.g., no change in the construct over time, no carry-over effects from the initial test). In other words, alpha estimates how accurately a test captures stable individual differences in the construct assessed.

A satisfactory measure must also demonstrate construct validity—it must adequately measure the construct of interest and only that construct [28]. Specifically, the measure should first exhibit content validity, covering all relevant features of the target construct, not excluding any important ones. Additionally the measure should also demonstrate convergent and discriminant validity, capturing the same construct as related measures, while being unrelated to measures for different constructs [29]. A measure of neuroticism, for example, should cover all its relevant facets and correlate highly with a measure of emotionality but not with a measure of honesty.

Finally, traditional instruments sometimes assess a related set of constructs, using a single instrument with multiple subscales. For example, the Five Factor Model uses five subscales to assess five domains of personality: extroversion, agreeableness, openness, conscientiousness, and neuroticism [11]. Hierarchical assessment models may contain further subscales. In the Five Factor Inventory, for example, each personality domain is associated with subscales that measure six facets of it [12]. For neuroticism, individual subscales assess the facets of anxiety, angry hostility, depression, self-consciousness, impulsiveness, and vulnerability. Regardless of how many subscales an instrument includes, the criteria of item coherence, test reliability, and construct validity apply to each.

### Limitations of traditional assessment instruments

A variety of issues bear on the use of traditional assessment instruments: judgment accuracy, situational variance, and theoretical understanding. Each is addressed in turn.

#### Judgment accuracy

What cognitive processes produce responses to decontextualized test items such as, “I am someone who perseveres until a task is finished”? It seems unlikely that one can consult a running average in memory for how efficiently one has performed recent tasks, much less over the past year or even a lifetime. So, how does an individual produce a response to this question? How accurate is it?

One possibility is that individuals have an intuitive theory about themselves that they consult [30–34]. According to this account, individuals don’t consult experiences in specific situations but instead draw inferences about themselves from abstract causal theories. Although these theories may reflect actual experience and be somewhat accurate, they may also reflect a variety of beliefs, goals, and biases. Another possibility is that individuals partially sample memories of situational experience but only access memories that are currently available, such that a biased sample results [35, 36]. To the extent that individuals use intuitive theories and/or the availability heuristic, responses to decontextualized test items are unlikely to be fully accurate [although they can be accurate to some extent; 37, 38].

#### Varying levels of a construct across situations

Considerable research shows that individuals do not exhibit a constant level of a construct, such as extroversion, across different situations [38–47]. Instead, the construct’s manifestation in behavior varies widely. Whereas an individual might be extraverted while dining with their family, they might be introverted while dining with co-workers.

From the classic theoretical perspective of interactionism [39, 48, 49], varying levels of extroversion expressed in different situations result from how the individual interacts with situations dynamically over time. Furthermore, expression in a particular situation does not simply result from a linear combination of the individual and the situation. Instead, different individuals respond to the same situations differently, such that an individual by situation interaction results.

It follows that assessing a construct in different situations for each individual is likely to be informative for characterizing individual differences in it. It further follows that predicting an individual’s behavior will be more accurate when taking specific situations into account than when using only trait-level information about the individual [37, 38, 43, 50–53].

#### Explaining constructs theoretically

Finally, simply establishing a fixed level of a construct in an individual offers little theoretical insight into it. Often it’s assumed that the level established represents a stable reified disposition or trait within the individual that operates consistently across situations [54]. Many personality researchers, however, question whether such dispositions literally exist within an individual’s cognitive-affective system. Saying, for example, that an individual is neurotic doesn’t necessarily mean that an enduring internal disposition for neuroticism exists in an individual’s cognitive-affective system, producing related behavior.

These kinds of trait attributions make the same error as concluding that a disposition of *briskness* exists in external climate conditions when one perceives the weather as “brisk.” Instead, briskness, like neuroticism, is a *perception* that says more about the perceiver than it does about mechanisms in the perceived entity that led to that perception. To mechanistically explain an individual’s level of neurotic behavior in specific situations, it is essential to establish the underlying cognitive and affective mechanisms that interact with specific situations to produce varying levels of neuroticism within specific situations, as well as stable perceived tendencies in behavior across them [38–42, 44, 45, 47].

### The Situated Assessment Method (SAM^2^)

SAM^2^ is a general assessment framework that can be used to measure diverse constructs, including stress, trichotillomania, eating, social connectedness, and sustainable behavior. We developed the SAM^2^ Habitual Behavior Instrument (SAM^2^ HBI) for several reasons: First, no instrument currently establishes habitual behaviors for an individual across a broad range of human activities. Second, no current instrument evaluates how extensively individuals vary in habitual behavior, nor addresses factors that predict these individual differences. Third, no current instrument establishes individual difference measures for positive versus negative habits and relates them to measures of personality, such as self-control and neuroticism. Fourth, a large literature on habits offers considerable guidance for developing a psychometric instrument that measures habitual behavior. Finally, the SAM^2^ HBI has potential to address important unresolved issues concerning the definition of habit and the relation of reward to habitual behavior.

Developing a SAM^2^ assessment instrument requires three steps: (a) Identify a representative set of situations where the target construct occurs and establish memory cues for sampling them, (b) Identify an appropriate measure of the target construct, (c) Identify scientifically-established factors in the Situated Action Cycle known to influence the target construct, maximizing coverage of influential factors. The next three subsections address each step in turn.

#### Measuring situational experience

SAM^2^ is designed to assess individual differences for a construct in situations where it occurs. Instead of asking individuals to generalize about the construct across situations without taking them into account explicitly, SAM^2^ first identifies situations where the construct is likely to occur and then asks individuals to assess the construct in each.

Experience sampling offers one approach for assessing situations, where participants assess the behavior of interest at randomly sampled moments in everyday life [38]. Two factors limit this approach for psychometric purposes. First, the time and effort needed to collect experience sampling data lies beyond the scope of many psychometric assessments. Second, the random nature of experience sampling limits the ability to control the situations assessed (i.e., the situations sampled cannot be determined in advance and vary widely across individuals).

For these reasons, SAM^2^ uses features of situations to cue relevant experiences of situations from memory across individuals systematically. As much research has established, a diverse collection of features constitutes a situation, including spatiotemporal context, people, behaviors, objects, events, emotions, and so forth [55–57]. When a situation is experienced, an episodic memory is established that represents its features [58–61]. If, for example, someone runs for exercise in a park one day at sunrise, a memory of the episode becomes established that contains features for running, exercise, park, and sunrise.

On later occasions, when one of these features is encountered, it can activate the memory via content-addressable retrieval mechanisms [62]. If, for example, someone is asked how regularly they exercise, the word “exercise” may activate this situational memory and other situational memories like it. In this manner, a feature of a situation can activate situational experiences. Indeed, memory researchers have used this approach to activate and study memories for decades, as exemplified by the Galton-Crovitz method and many others [63–66].

In the typical SAM^2^ instrument, a set of situational features is developed to cue relevant situational experience of interest. By using a constant set of features across individuals, SAM^2^ systematically assesses situations across individuals efficiently in a psychometric instrument. We assume further, however, that a given feature, such as “exercise,” activates widely differing situational memories between individuals, thereby establishing individual differences of interest.

Our approach to sampling situations is similar to other approaches that sample important life events [67, 68], repetitive daily events [69], and situated interaction [70]. Like SAM^2^, all these approaches emphasize the importance of taking an individual’s rich situational experience into account. Our approach also resonates strongly with approaches that incorporate the rich structure of everyday experience into psychological research [71–75]. Finally, our approach follows longstanding traditions in personality and social psychology that champion the importance of situations in understanding cognition, emotion, and behavior [38–40, 45, 76, 77].

*Measuring situational experience with the SAM*^*2*^ *HBI*. The SAM^2^ HBI uses behaviors to activate relevant situational memories. Specifically, as Table 1 illustrates, the SAM^2^ HBI uses 80 common behaviors likely to be habitual from 10 domains of human activity. Because our initial study assessed habitual behavior in college students, we selected behaviors relevant for this population. All later studies used the same 80 behaviors and continued to assess them in the student population. The 10 behavior domains were established by reviewing the literature on habits and exploring online resources associated with habit change. Our aim was to establish a broad representative range of common behaviors likely to be habitual across diverse life activities, ranging from food and drink to protecting the environment.

**Table 1:** The 80 behaviors assessed for all participants (4 positive and 4 negative from each of 10 domains).

Positive behaviors	Negative behaviors
**Food and Drink**	
1. Eat fruit	5. Drink alcohol
2. Eat healthy snacks	6. Eat dessert
3. Eat vegetables	7. Eat fast foods
4. Check food labels before making purchases	8. Drink soft drinks
**Exercise**	
9. Exercise	13. Be sedentary for long periods of time
10. Walk or bike when possible	14. Avoid long walks
11. Participate in sports activities and clubs	15. Reward myself with food and/or drink after exercise
12. Take standing and walking breaks when sitting for long periods of time	16. Use the lift instead of taking the stairs
**Affective**	
17. Take time to relax	21. Use substances to relax
18. Do at least one thing a day that I enjoy and look forward to	22. Worry
19. Express my emotions constructively	23. Criticise myself
20. View challenges with a positive attitude	24. Ignore my own needs
**Social**	
25. Maintain contact with family	29. Use bad language in public
26. Maintain contact with friends	30. Interrupt others
27. Hold doors open for others	31. Pay little attention to others when they are talking
28. Say ‘please’ and ‘thank you’	32. Make myself the centre of conversation
**Technology**	
33. Make back-up copies of important documents and files	37. Spend a large amount of time on social media
34. Charge my devices	38. Use my phone as a social crutch (e.g. use my phone when I am alone in social situations)
35. Limit the amount of time each day I spend using technology	39. Check my phone multiple times a day
36. Restrict my use of technology before sleep	40. Use my phone whilst on the toilet
**Work and Study**	
41. Study for my course(s)	45. Procrastinate
42. Take study breaks	46. Work whilst watching TV or listening to music
43. Set goals before engaging in a task	47. Skip lectures
44. Pack what I need the night before	48. Multi-task during work
**Personal Hygiene**	
49. Shower every day	53. Pick my nose
50. Cover my mouth when sneezing, coughing or yawning	54. Pick my spots and/ or scabs
51. Brush my teeth twice a day	55. Chew on pencils and/ or pens
52. Go to sleep and wake up at the same times	56. Bite my nails
**Household**	
57. Wash my clothes	61. Allow messes to build up in my work area
58. Put things back after I have finished using them	62. Ignore stains and spills
59. Empty the bins	63. Leave dishes to wash later
60. Clean my residence	64. Leave clothes lying around
**Finance**	
65. Budget	69. Dip into funds I have set aside
66. Buy from charity and/ or second-hand shops	70. Spend to make myself feel better
67. Use shopping lists	71. Buy brand name products
68. Shop for groceries	72. Make impulsive purchases
**Environment**	
73. Turn off lights when leaving a room	77. Litter
74. Recycle	78. Buy new condition items
75. Reuse carrier bags	79. Leave plug sockets switched on
76. Use reusable cups	80. Throw away food

We do *not* assume that every behavior in Table 1 is a habitual behavior for *every* individual. Instead, we simply assume that these are common habitual behaviors in the population. We offer evidence for this claim later, showing that most of these behaviors were highly habitual for at least some individuals. Within each domain, we manipulated behavior valence, sampling both positive (good) and negative (bad) behaviors. As also discussed later, Study 2 provided strong support for the a priori valence assignments in Table 1, along with insight into the valence distinction: Whereas the positive behaviors were highly associated with long-term reward and social approval, the negative behaviors were not.

#### Measuring the target construct

For each individual, our goal was to establish the habitualness of each behavior in Table 1. Many measures of habitualness have been proposed, including the frequency of a behavior, the consistency of the situations that elicit it, the automaticity of the behavior, and the automaticity of the cognitions that precede it. Considerable discussion continues to surround these issues [78–92].

Our take on this literature is that the most fundamental property of a habitual behavior is *the regularity of performing it in situations where doing so is possible*. The raw frequency of performing a behavior is *not* the critical determinant of habitualness—instead it is the regularity of performing the behavior in relevant situations. From this perspective, attending Christmas Eve mass every year is as habitual as turning off the lights every time one leaves a room, even though the latter occurs much more frequently. Thus, for each behavior in Table 1, we asked participants to evaluate, “How frequently do you perform this behaviour in situations where doing so is possible?” (on a 0 to 100 scale). Again, we assume that each behavior cues situational memories that participants evaluate to produce a judgment.

It is certainly possible that behaviors performed regularly are also performed automatically in consistent situations [82, 87]. Many researchers, however, argue that habitual behaviors are not always performed automatically [80, 85, 88, 92]. From the latter perspective, it is an empirical question whether behaviors performed regularly are also performed automatically (and/or consistently). To assess this issue, we included both automaticity and consistency as factors from the Situated Action Cycle that potentially influence regularity (described later).

*Establishing situational variability*. By assessing how regularly participants perform the 80 behaviors in Table 1, we can establish classic situational effects (via situational memories that the behaviors activate). To the extent that some behaviors are performed more regularly than others, regularity should generally be higher in the associated situations across individuals. Interactions between situations and individuals can also be assessed similarly. To the extent that different individuals exhibit different patterns of regularity across the same behaviors, individuals should interact with the associated situations differently.

*Establishing situation-based trait-level measures*. Although constructs like regularity are likely to exhibit considerable variability across behaviors and their associated situations, they can also exhibit surprising stability [38, 46, 93, 94]. When the varying levels of a construct across situations are aggregated (e.g., averaged), the resulting ‘trait-level’ measure tends to be highly stable, typically correlating .7 to .9 with comparable aggregate measures of the same construct. Individuals, for example, whose aggregate neuroticism is high across multiple assessments over a week tend to also exhibit high neuroticism across multiple assessments the following week. Although one might be tempted to reify this stability into an internal trait mechanism, a more plausible approach is that it reflects an individual-specific configuration of cognitive-affective processes that produces a relatively stable pattern in behavior while simultaneously adapting to different situations dynamically [38–40, 45, 48].

By aggregating assessments of regularity for an individual across the behaviors in Table 1, SAM^2^ establishes trait-level information about the construct in the associated situations. Importantly, trait-level information in SAM^2^ is established empirically, statistically aggregating levels across behaviors. As a consequence, trait-level measures in SAM^2^ are likely to differ from traditional decontextualized measures, which again may instead reflect intuitive theories and the availability heuristic. Previous comparisons of these two approaches for establishing trait-level information do indeed demonstrate significant differences [38, 95].

*Item coherence*. As we saw earlier, traditional assessment instruments attempt to maximize coherence of the items that assess a latent construct, thereby capturing the construct on a single factor. In contrast, item coherence is not necessary in SAM^2^ instruments, nor even desirable. In SAM^2^ instruments, the construct of interest is assessed by cuing a representative sample of situations where the construct occurs. When these situations differ significantly, each is likely to order individuals differently. Individuals exhibiting high levels of the construct in one situation may exhibit low levels in another. As a consequence, large individual by situation interactions emerge, producing low item coherence (i.e., situations do not load on a single factor). While this may not appear desirable from the traditional assessment perspective, it *is* desirable from the SAM^2^ perspective, reflecting the sometimes necessary cost of assessing a target construct comprehensively across a diverse sample of situations.

The Spearman-Brown formula indicates how to compensate for low item coherence [23–27]. When item coherence is low, assessing a construct with *a large number of test items* can establish satisfactory test reliability of the aggregate measure. Imagine that coherence is only .05 across situations for a SAM^2^ instrument. If 80 situations are assessed, the Spearman-Brown formula estimates that alpha will be .81, indicating adequate test reliability. Because the aggregate measure is based on so many items, it is reliable, even though coherence is low. Moreover, using such a large number of situations increases the likelihood that the situations assessed do a reasonable job of covering situations representatively where the construct occurs.

#### Measuring situational experience with respect to the Situated Action Cycle

To more completely understand a construct in the situations where it occurs, it is useful to establish its role in the embodied goal-directed action that transpires in them. Thus, SAM^2^ doesn’t simply measure a construct in relevant situations, it measures additional factors of these situations likely to influence the construct. By doing so, SAM^2^ establishes both content and convergent validity, comprehensively measuring situational factors related to the target construct of interest.

**Fig 1:**
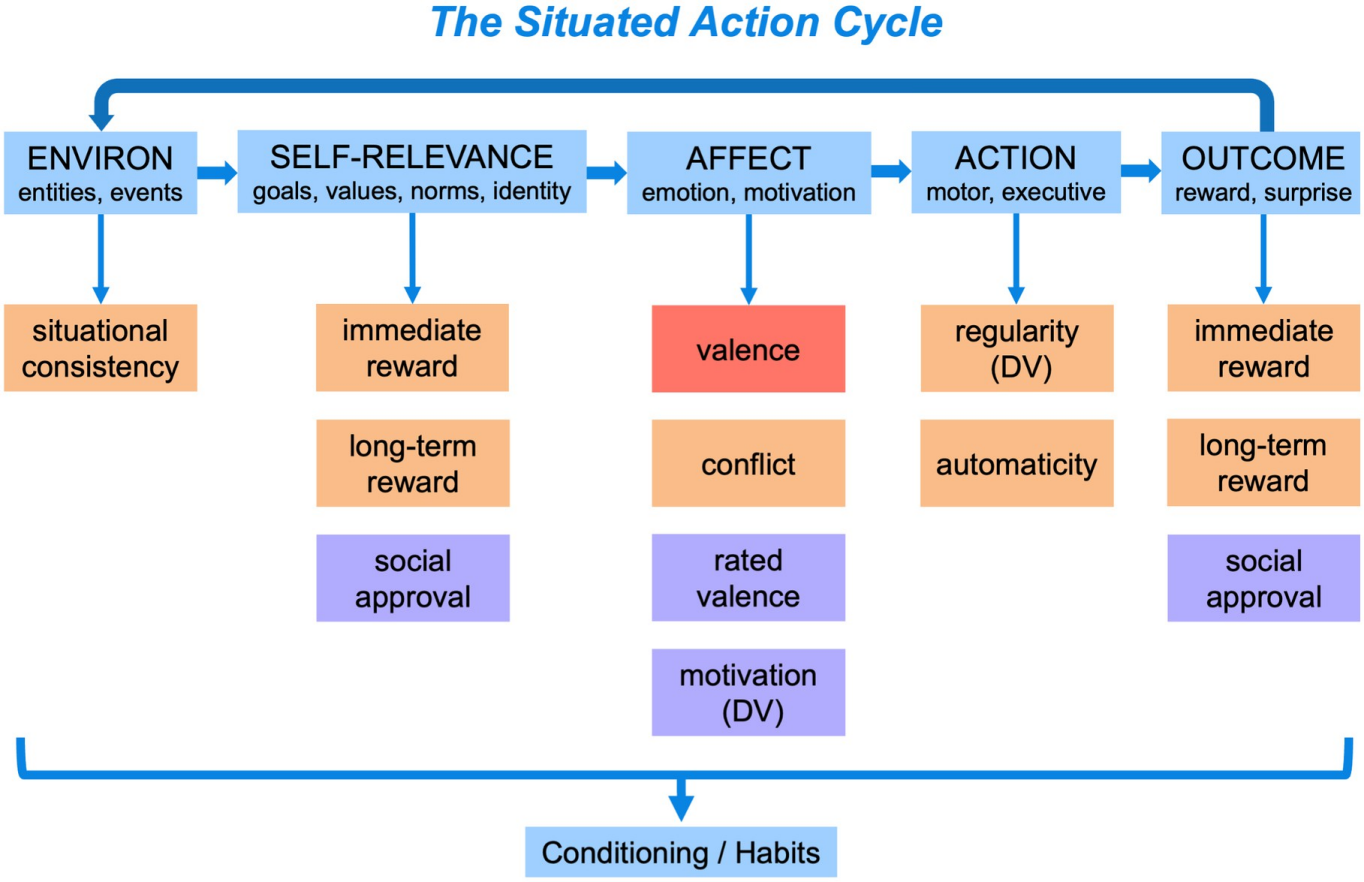
The five phases of the situated action cycle, along with measures sampled from it for the SAM^2^ habits instrument. *Note*. Valence in red was assigned a priori during the behavior selection process and validated later in Study 2. Measures in orange were assessed across Studies 1-all, Study 2, and Study 3. Measures in purple were only assessed in Study 2. In later regression analyses, regularity and motivation were dependent variables (DVs), and all other measures were predictors. Measures for the self-relevance and outcome phases of the situated action cycle are the same, given that they can be anticipated initially as self-relevant and then occur later as outcomes.

The Situated Action Cycle provides a powerful theoretical tool for identifying factors that influence a target construct [96]. Fig 1 illustrates the Situated Action Cycle as an idealized series of discrete linear phases. In actual operation, these phases may overlap in time, occur iteratively, or be omitted. Also, various loops and alternative relations between phases may emerge. The idealized representation in Fig 1 simply illustrates critical phases of the Situated Action Cycle and their approximate relations to each other.

As Fig 1 illustrates, phases of the Situated Action Cycle include: the environment, self-relevance, affect, action, and outcomes. In the environment phase, agents experience situations that offer affordances for action and trigger habitual behavioral patterns. In the self-relevance phase, entities and events in the environment implicitly activate self-relevant goals, values, identities, norms, etc. Once a situation’s relevance for the agent is established, it initiates diverse affective states associated with emotion and motivation, often expressed in the body. In turn, emotion and motivation induce diverse actions, ranging from gross bodily movements to eye movements and executive functions. Finally, the actions performed lead to outcomes, such as reward and prediction error. Fig 1 represents the iterative character of the situated action cycle with an arrow that loops back from outcomes to resultant changes in the environment that initiate the next cycle. Fig 1 further represents how each iterative run contributes to conditioning and habit development in long-term memory.

The Situated Action Cycle is hardly a novel idea, with variants playing central roles across disciplines for decades [96]. Behavioral conditioning, for example, offers a classic example of the Situated Action Cycle in psychology, where environmental cues signal reward outcomes that can be obtained with instrumental actions [97, 98]. In cognitive science, theories of goal pursuit offer a different take on the Situated Action Cycle, inserting internal cognitive and affective states between the environment, action, and outcomes central to behaviorism. Examples of this expanded approach can be found in computational accounts of planning and problem solving [99, 100], production systems [101, 102], reinforcement learning [103, 104], and neuroscience [105–107]. In discourse analysis, theories of narrative structure propose that conceptual structures similar to the Situated Action Cycle organize people’s knowledge of events during event processing and autobiographical memory [108–111]. Grounded theories of conceptual processing similarly propose that knowledge becomes organized around situated action [5, 6, 96, 112, 113]. Because the Situated Action Cycle captures an organized set of processes central to many activities and processes, its ubiquitous role across disciplines is no accident. For this reason, measuring factors across phases of the Situated Action Cycle that influence a target construct is likely to produce comprehensive and informative individual difference measures in a psychometric instrument.

*Assessing factors from the Situated Action Cycle in the SAM*^*2*^ *HBI*. To establish factors from the Situated Action Cycle that influence behavior regularity, we turned to the large literature on habitual behavior. The red, orange, and purple boxes in Fig 1 illustrate the specific factors in the literature we elected to assess in the studies that follow.

Two important factors associated with habitual behavior are consistency and automaticity. On the one hand, consistency contributes to the development, learning, and triggering of habitual behavior [78, 87, 97, 114, 115]. On the other, as triggering stimuli become frequently and consistently mapped to related responses, automaticity results in both cognition and behavior from repetitive practice [81, 82, 90, 91, 116–118]. Considerable literature documents the roles of consistency and automaticity in the conditioning process that produces habitual behavior. Based on this literature, we asked participants to assess the consistency and automaticity of each behavior, predicting that both would be strongly related to behavior regularity, while exhibiting individual differences.

A contentious issue in the literature is whether reward modulates habitual behavior or not. To assess this issue, we asked participants in all studies to evaluate both the immediate and the long-term reward of each target behavior. Based on extensive evidence that reward plays a central role in habitual behavior [86], we predicted that both forms of reward would be positively related to behavior regularity at the group level [also see 88, 89, 92, 97, 101]. We further predicted, however, that consistency and automaticity would be more strongly related to habitual behavior than reward, reflecting the dominant effects of conditioning that make habitual behavior somewhat ballistic. Again, however, we expected to observe large individual differences.

Further literature suggests that as a behavior becomes increasingly habitual, the amount of self-regulation accompanying it decreases [119–121]. As a consequence, the amount of conflict experienced with performing the behavior decreases as well. Whereas conflict should arise as people explicitly decide—and perhaps struggle—with what behavior to perform in a particular situation, conflict should decrease as they settle on a single behavior that becomes increasingly habitual. Thus, we predicted that conflict would decrease as behavior regularity increased, again though, expecting individual differences.

*Assessing relations between personality and habitual behavior*. Previous literature shows that self-control is associated with increased performance of positive behaviors and decreased performance of negative behaviors [88, 122–125]. Conversely, previous literature shows that neuroticism is associated with increased performance of negative behaviors and decreased performance of positive behaviors [126–129]. To assess whether the SAM^2^ HBI captures these well-established interactions between personality measures and behavior valence, we included traditional measures of self-control and neuroticism. We also assessed whether factors from the Situated Action Cycle in Fig 1 offer insight into the cognitive and affective processes associated with these personality dispositions.

## Methods

### Overview of methods in the individual studies

Study 1a was an initial exploratory assessment of the SAM^2^ HBI performed in our University of Glasgow laboratory (*n* = 31). Study 1b was an exact replication of Study 1a, again performed in our Glasgow laboratory (*n* = 31). Study 1c was an exact replication of Studies 1a and 1b combined, performed online with participants sampled across the UK from the Prolific platform (*n* = 66). Because these three studies were identical except for the details above, and because they exhibited comparable results, they were combined into “Study 1-all” (*n* = 128).

Study 2 was an online replication of Study 1-all with a larger UK-wide sample from Prolific (*n* = 199), while assessing three additional factors from the Situated Action Cycle: rated valence, social approval, and motivation (the purple factors in Fig 1).

Study 3 was another replication of Study 1-all using a UK-wide sample from Prolific (*n* = 115). Whereas Study 1-all and Study 2 collected judgments for regularity, consistency, immediate reward, long-term reward, conflict, and automaticity in a fixed blocked order, Study 3 randomized the order of assessing the predictive factors individually, thereby assessing whether the specific format for collecting judgments was important. Study 3 also added an additional metacognitive measure that assessed whether participants were aware of the factors that predicted their regularity judgments.

### Common methods across studies

#### Participants

All participants were required to be fluent English speakers, UK residents, and students aged 18 to 30. Online participants (paid £6/hour) were required to have participated in at least 10 Prolific studies and to have achieved at least a 95% approval rate. No participants were excluded in Studies 1a, 1b, 1c, or 2. In Study 3, two participants were excluded for taking very little time and responding randomly on measures that assessed information in the data (established prior to hypothesis testing).

#### Design

All participants assessed the same 80 behaviors in Table 1 (40 positive, 40 negative) for the 6 orange factors from the Situated Action Cycle in Fig 1 (behavior regularity, consistency, immediate reward, long-term reward, conflict, automaticity). Additionally, all participants received a score for the Brief Self-Control Scale and for the five subscales of the Five Factor Inventory (extroversion, neuroticism, agreeableness, conscientiousness, openness to experience). In a multilevel design, behavior regularity served as the dependent variable, with consistency, immediate reward, long-term reward, conflict, and automaticity predicting regularity at the behavior level, and with self-control and neuroticism predicting regularity at the individual level.

#### Materials

To cover a broad range of potential habits, we first included behaviors addressed regularly in the habits literature and then turned to internet sites that list habits and help people work with them. Besides attempting to cover as many relevant domains as possible, we manipulated behavior valence, including both positive and negative behaviors (Table 1).

Study 2 assessed the validity of our a priori assignments for valence, with all results reported in the supplemental materials, S1 File. As shown there, rated valence in Study 2 verified that the positive behaviors in Table 1 were strongly positive and that the negative behaviors were strongly negative, with no overlap. Regressions further found that the valence manipulation reflected SAM^2^ predictors from the Situated Action Cycle, with positive behaviors being high in long-term reward and social approval, and with negative behaviors being low.

#### Procedure

All participants performed the study online in an anonymous Qualtrics survey. Participants first received an information sheet about the study and clicked a digital form to provide informed consent (ethics approval for this work was received on 1 August 2017 from the Ethics Committee for the College of Science and Engineering at the University of Glasgow, Application 300160200).

Participants then evaluated the 80 behaviors in blocks for behavior regularity, consistency, immediate reward, long-term reward, conflict, and automaticity. Table 2 presents the scales used to collect these judgments. As described shortly, the format for collecting these judgments varied across Studies 1-all, 2, and 3. Additionally, Study 2 included judgments for habit motivation, valence, and social approval (described in S1 File).

**Table 2:** Measures assessed in Studies 1-all, 2, and 3, with interrater agreement for each.

Scale Name / Query / Values / Labels	Study:	Interrater agreement between individuals when judging behaviors (ICC2)								
		All 80 behaviors			40 positive			40 negative		
		1-all	2	3	1-all	2	3	1-all	2	3
**Regularity**		.32	.28	.28	.26	.25	.25	.30	.25	.27
How frequently do you perform this behaviour in situations where doing so is possible?										
(0 to 100) (Never, Occasionally, Half the time, Regularly, Always)										
**Consistency**		.18	.14	.20	.16	.13	.18	.16	.12	.18
How often do you perform this behaviour in same situations, or at the same places and times?										
(0 to 100) (Never, Occasionally, Half the time, Regularly, Always)										
**Immediate Reward**		.22	.19	.21	.15	.12	.13	.23	.18	.25
How much do you want to perform this behaviour because it will feel good in the moment?										
(-5 to 5) (Not at all, Somewhat, A lot)										
**Long-Term Reward**		.59	.48	.53	.09	.09	.09	.13	.13	.13
How much do you want to perform this behaviour because it will be good for you in the long term?										
(-5 to 5) (Not at all, Somewhat, A lot)										
**Conflict**		.16	.08	.10	.14	.06	.08	.12	.08	.10
How conflicted do you feel about wanting vs. not wanting to perform this behaviour?										
(0 to 100) (Never, Occasionally, Half the time, Regularly, Always)										
**Automaticity**		.22	.17	.18	.22	.16	.18	.21	.17	.20
How much do you perform this behaviour automatically with little thought or effort?										
(0 to 100) (Never, Occasionally, Half the time, Regularly, Always)										

**Note.** The left side presents the scale for each of the six measures common across Studies 1-all, 2, and 3. The right side presents values for inter-rater agreement as measured by intra-class correlations (specifically, the ICC2 measure from the ICC function in the R Psych package). In this context, the intra-class correlation estimates how much different individuals agree in how they order behaviors from high to low on a given measure (interrater agreement). Because the ICC2 form of the intraclass correlation estimates random effects, these values are likely to generalize across other individuals from the same population. Values of the ICC2 across all 80 behaviors are shown on the left; values for the 40 positive behaviors are shown in the middle; values for the 40 negative behaviors are shown on the right.

After making these judgments, participants completed the Five Factor Inventory [14] and the Brief Self-Control Scale [15]. Finally, participants provided demographic information, were debriefed, and thanked for their participation. Participants typically took about 50 to 60 min to complete the study, with Studies 2 and 3 taking a little longer than Study 1-all. To ensure that the construct of *habit* was not salient to participants, the term “habit” was not mentioned in Studies 2 and 3 and was mentioned only once in the introduction of Study 1-all.

### Analyses

Analyses performed include computations of intraclass correlations [25, 26], Cronbach’s alpha [23, 24], the Spearman-Brown formula [24, 26, 27], and multilevel mixed-effect regressions [130, 131]. S1 File presents the analysis pipeline used for all regression analyses.

Most analyses to follow focus on effect size, replicability, and generalizability, with little formal hypothesis testing [132–134]. We report *p* values when inferences about statistical significance are potentially informative.

### Unique methods for individual studies

#### Data collection mode

To assess whether our results replicated across different modes of data collection, Studies 1a and 1b were performed in our laboratory, whereas Studies 1c, 2, and 3 were performed online.

#### Data collection format

In Studies 1a, 1b, and 1c, participants performed three blocks of judgments. In the first block, participants assessed each of the 80 behaviors one at a time for both regularity and situational consistency. In the second block, participants assessed each behavior for both immediate reward and long-term reward. In the third block, participants assessed each behavior for both conflict and automaticity. In each block, the same 80 behaviors were randomly ordered for each participant. S1 File presents our reasons for blocking judgments in this manner, together with results that justify our reasoning.

In Study 2, the data collection format was the same as the procedure of Study 1-all, with the following exceptions. Judgments of behavior motivation were added to block 2, and a new block 4 was added that included rated valence and social approval. Blocks 1 and 3 were the same as in Study 1-all. To streamline the main text here, results for behavior motivation, rated valence, and social approval are only presented in S1 File. In all cases, they complement the main results reported here and do not raise issues that bear on them.

In Study 3, only one judgment was made on each trial instead of two or three. Because behavior regularity was the dependent variable, it was always assessed in a first block of the 80 randomized behaviors (so as not be influenced by the subsequent blocks for the five predictive factors). The predictive factors (consistency, immediate reward, long-term reward, conflict, automaticity) were then assessed one at a time in five subsequent blocks randomized for each participant, with the 80 behaviors randomized within each. Of interest was whether collecting unblocked judgments with the predictive factors in a random order replicated the pattern of results obtained with the blocked judgment procedure.

S1 File provides examples of the screens used to collect judgments in all studies. S1 File also provides the rationale for implementing blocked judgments initially in Studies 1-all and 2. As will be seen in the main text, data collection format had no impact on the general pattern of results obtained. S1 File presents additional evidence bearing on this issue.

#### Metacognitive judgments

In Study 3, participants were asked to estimate how much influence each of the SAM^2^ factors—consistency, immediate reward, long-term reward, conflict, and automaticity—has on them when performing the 80 behaviors in Table 1 (just prior to filling out the Five Factor Inventory and Self-Control instruments). S1 File presents results showing that participants were relatively unaware of the factors associated with their regularity judgments.

## Results

For all results in the main text and S1 File, data and analysis scripts are publicly available online. S1 File presents the correlation matrix for the measures common across studies, along with judgment means for the 80 behaviors across SAM^2^ measures.

### Individual differences in behavior regularity

The SAM^2^ HBI provides an overall trait-level measure of an individual’s behavior regularity. Averaging an individual’s 80 regularity judgments provides an aggregate measure of how regularly they perform the 80 behaviors in Table 1. The median of these individual means in Studies 1-all, 2, and 3 was 54.91, 56.25, and 56.29, respectively, with inter-quartile ranges of 24.27, 21.60, and 21.46. These values provide a general sense of behavior regularity in the population for these kinds of behaviors, and for how much it varies across individuals. Because the scale value of 50 was labeled, “half the time,” these values indicate that participants performed the 80 behaviors in Table 1 over half the time, on average, in situations where possible, with some participants exhibiting more regularity and others exhibiting less.

**Fig 2:**
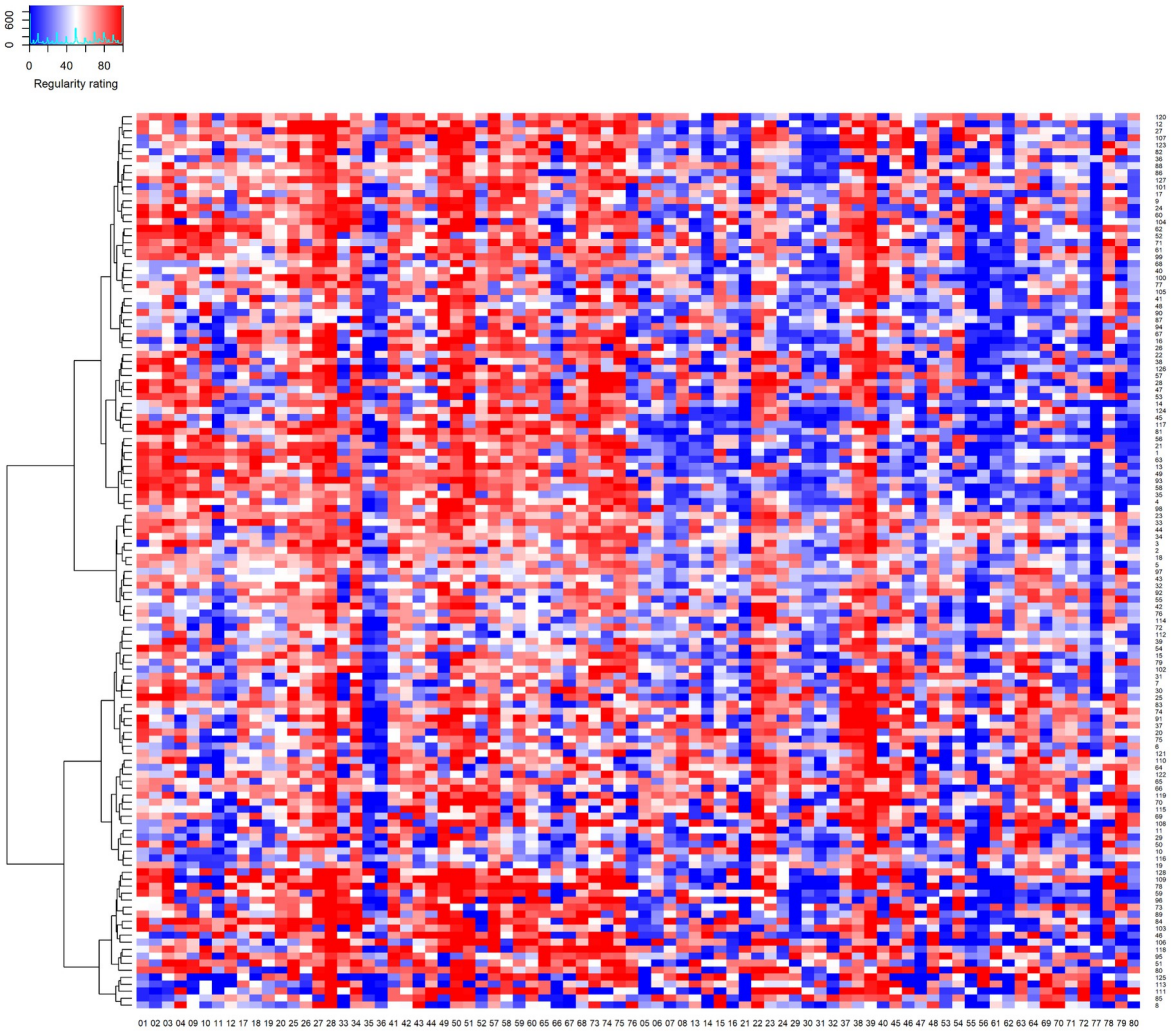
A heatmap that visualizes the individual × behavior interaction for behavior regularity in Study 2. *Note*. The 80 regularity judgments for each of the 199 participants are presented in a single row, with their judgments for 40 positive behaviors in the left half, and their judgments for 40 negative behaviors in the right half. The number below each column corresponds to the number of the corresponding behavior in Table 1. As a cell becomes increasingly red, the regularity judgment increasingly approached 100 (on a scale of 0 to 100). As a cell becomes increasingly blue, the regularity judgment increasingly approached 0. As a cell becomes increasingly white, the regularity judgment increasingly approached 50. On the left, a hierarchical clustering dendrogram establishes groups of participants having similar vectors of regularity values across positive and negative behaviors (from hierarchical clustering with the Ward D measure). Analogous heatmaps for Studies 1-all and 3 can be found in S1 File. Table 1 provides intraclass correlations that assess interrater reliability of the judgments in this map.

It is important to note, however, that a given individual performed many behaviors much more regularly than their overall mean, indicating that some behaviors were performed very regularly. To see this, consider the heatmap in Fig 2 that visualizes each participant’s regularity judgments in Study 2 (S1 File presents analogous heatmaps for Studies 1-all and 3). The number below each column corresponds to the number of the corresponding behavior in Table 1 (with positive behaviors on the left and negative behaviors on the right). When a participant performed a behavior 50% to 60% of the time when possible, its cell is relatively white in the heat map. As can be seen, however, the cells for many behaviors are bright red, indicating that they were performed 80% to 100% of time. Later when we address an individual’s most habitual behaviors, we will again see that many behaviors were performed with very high regularity.

**Fig 3:**
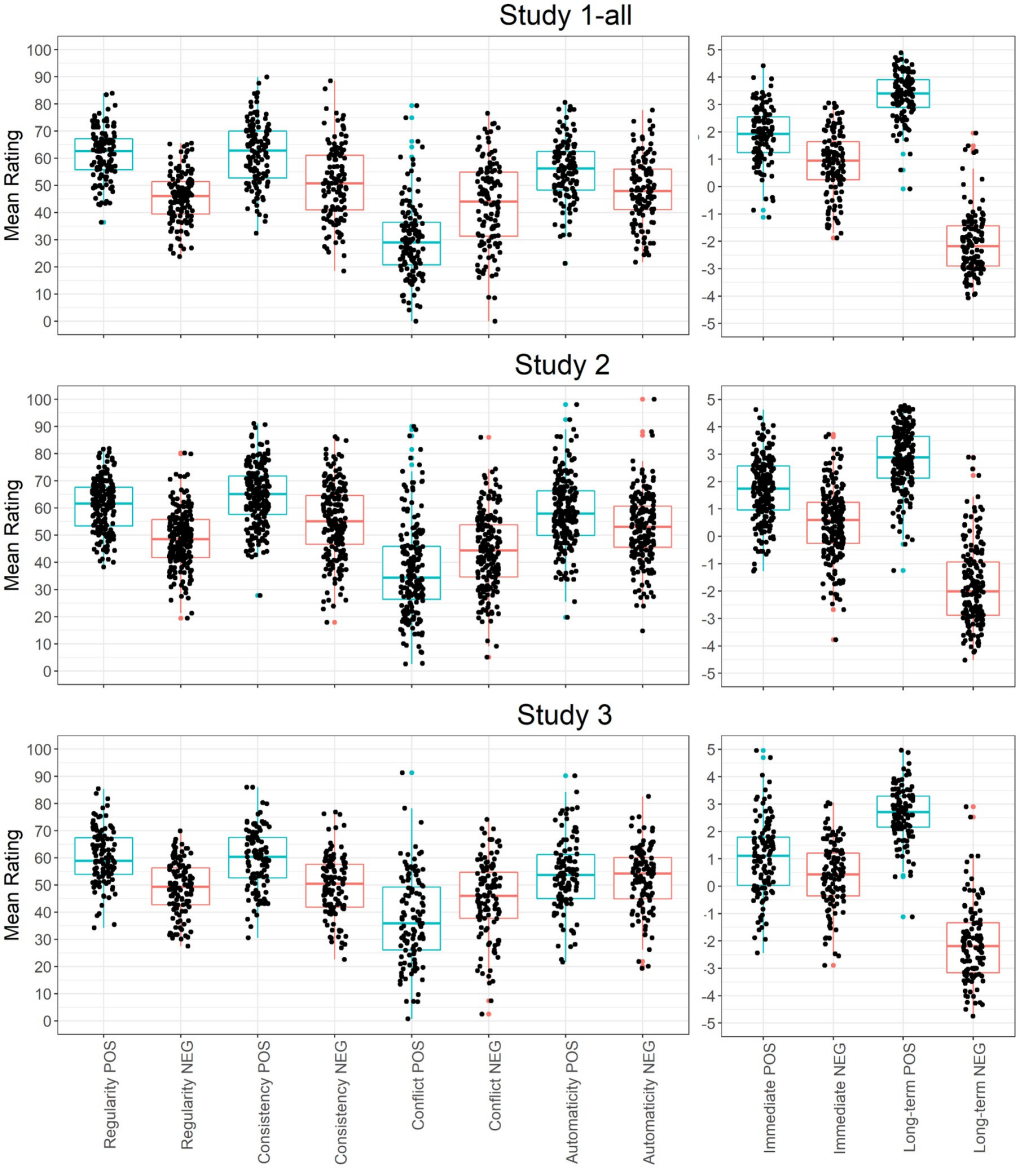
For each individual participant, the mean judgment on each measure (regularity, consistency, immediate reward, long-term reward, conflict, automaticity) for positive (POS) and negative (NEG) behaviors. *Note*. Each point is the mean judgment across 40 behaviors for a single individual. Box and whisker plots show the median and inter-quartile range of the means across participants for each measure.

The SAM^2^ HBI further provides trait-level measures for how regularly individuals perform positive and negative behaviors. Fig 3 presents two means for how regularly each individual performs the 40 positive behaviors and the 40 negative behaviors in Table 1. Across studies, the median of these participant means was around 60 for positive behaviors and around 50 for negative behaviors, indicating that positive behaviors were performed more regularly than negative behaviors. Again, however, the bright red cells on the left and right of Fig 2 illustrate that a given individual performed many positive and negative behaviors 80% to 100% of the time.

Interestingly, trait-level measures for the regularity of positive and negative behaviors tended to be negatively correlated. When the mean regularity of performing positive behaviors was correlated with the mean regularity of performing negative behaviors across participants, the correlations were -.22 in Study 1-all, -.03 in Study 2, and -.21 in Study 3 (*t*(126) = -2.54, *p* = .01; *t*(197) = -.16, *p* = .71; *t*(113) = -2.34, *p* = .02). As individuals tended to perform positive behaviors more regularly, they did not also tend to perform negative behaviors more regularly. In other words, these two individual difference measures exhibited divergent validity, measuring different trait-level constructs in an individual. Later we will see that personality measures for self-control and neuroticism integrate these divergent measures of positive and negative regularity, and that factors from the Situated Action Cycle explain relations between them.

The SAM^2^ HBI provides additional individual difference measures for factors from the Situated Action Cycle that influence habitual behavior. Consider the individual means for consistency and automaticity in Fig 3, whose median values were generally around 50 or higher across studies. These relatively high values indicate that the 80 behaviors in Table 1 tended to be performed automatically in consistent situations—hallmarks of habitual behavior.

Fig 3 also presents the individual means for immediate reward and long-term reward across positive and negative behaviors. As can be seen, the median values for immediate reward tended to be greater than 0, indicating that participants tended to find both positive and negative behaviors immediately rewarding. Perhaps surprisingly, positive behaviors were consistently associated with higher immediate reward than were negative behaviors. As Fig 3 further illustrates, long-term reward behaved more as expected, with a positive median value for positive behaviors but with a negative median value for negative behaviors.

Overall, the pattern of results for regularity, consistency, automaticity, immediate reward, and long-term reward across positive and negative behaviors offers construct validity for the SAM^2^ HBI. Across the Situated Action, Cycle factors related to habitual behavior reflected well-established patterns in the literature.

### Individual differences in behavior regularity across situations

We anticipated that large differences would exist in how regularly individuals perform the 80 behaviors in Table 1. Specifically, we expected that the behaviors one individual performed regularly would differ considerably from the behaviors that another individual performed regularly—an individual by behavior interaction. To assess this prediction, we used the intraclass correlation to assess interrater agreement in how regularly participants performed the 80 behaviors [25]. Conceptually, the intraclass correlation in this context estimates the average correlation between all possible pairs of individuals in their regularity judgments across behaviors, establishing the overall similarity in their judgments (i.e., interrater agreement).

The first row of Table 2 presented earlier displays the intraclass correlations for behavior regularity across studies, with the lower rows presenting values for the other five measures from the Situated Action Cycle. Because these values were computed using the random effects version of the intraclass correlation [the ICC2 measure in 24, 25], they generalize beyond the current individuals in each study to other individuals in the larger population.

As the columns for all 80 behaviors in Table 2 illustrate, interrater agreement for behavior regularity was only around .30 across studies, suggesting large individual differences in how regularly individuals perform specific behaviors. On the average, the regularity of one individual’s behaviors only correlated about .30 with the regularity of another individual’s behaviors. As the additional values of the ICC2 illustrate for the 40 positive behaviors and for the 40 negative behaviors, comparable levels of interrater agreement for regularity tended to hold within each subset of behaviors as well. Although this low agreement between individuals could simply reflect noise, we will see later that it is highly systematic.

Fig 2 offers insight into the relatively low interrater agreement for regularity. As can be seen by comparing different columns with one another, regularity varied widely and systematically across the 80 behaviors. Nevertheless, the pattern of regularity across the 80 behaviors varied in different manners for different individuals, as can be seen by comparing different rows. It is these differences in patterns of regularity across rows that low interrater reliability in Table 2 is capturing.

In general, differences in the columns and rows of Fig 2 capture classic sources of variance associated with the interactionist approach [38–40, 45, 48]. First, individuals exhibit different trait-level forms of a construct, as captured by overall differences in regularity across rows. Second, a construct takes widely varying forms across behaviors (and their associated situations), as captured by overall differences in regularity across columns. Third, a construct exhibits an individual by behavior interaction, with the construct’s levels across behaviors exhibiting different patterns for different individuals (reflected in low interrater agreement).

Additionally, the dendrogram on the left of Fig 2 establishes clusters of individuals who exhibited similar patterns of regularity across the 80 behaviors. Whereas the cluster comprising the top half of Fig 2 captures individuals who perform positive habits more than negative habits, the cluster comprising the bottom half captures individuals who perform positive habits relatively less and negative habits relatively more. Within each of these large clusters, smaller clusters capture other profiles of habit regularity. For example, a cluster just above the midline captures individuals who perform positive habits the most and negative habits the least.

Finally, interrater agreement in Table 2 for other measures from the Situated Action Cycle varied widely, ranging from ~.10 for conflict to ~.50 for long-term reward, with automaticity (~.18), consistency (~.18), and immediate reward (~.20) falling in between. In general, these values indicate that the SAM^2^ HBI exhibits large individual differences across all its measures. The relatively high agreement for long-term reward across all 80 behaviors (~.50) most likely reflects the salient difference between positive and negative behaviors. When agreement was assessed separately for positive and negative behaviors, interrater reliability for long-term reward dropped considerably to ~.10, indicating much less agreement within each behavior subset.

### Test reliability

As discussed earlier, SAM^2^ establishes an overall trait-level measure of behavior regularity for each individual across the 80 behaviors in Table 1, along with more specific trait-level measures for positive and negative behaviors (Fig 3). Of interest next is how reliable these trait-level measures are (i.e., how accurately they capture individual differences in behavior regularity). We used Cronbach’s alpha to establish test reliability [23, 26; specifically the ICC3k measure in 24, 25].

The top row of Table 3 presents alpha for the three SAM^2^ measures of behavior regularity. As can be seen, these alphas are satisfactory, clustering around the conventionally acceptable level of .70 to .80. The rows immediately below similarly demonstrate acceptable values for factors from the Situated Action Cycle that influence behavior regularity.

**Table 3:** Test reliability and inter-behavior coherence for all measures in Studies 1-all, 2, and 3.

Judgment	Study:	Cronbach’s alpha (ICC3k) (test reliability of aggregated participant scores)								
		All behaviors			Positive behaviors			Negative behaviors		
		1-all	2	3	1-all	2	3	1-all	2	3
regularity		.74	.82	.78	.82	.83	.86	.80	.84	.83
consistency		.91	.91	.87	.85	.85	.87	.88	.89	.85
immediate reward		.85	.88	.88	.89	.90	.93	.89	.89	.89
long-term reward		.83	.87	.90	.87	.91	.90	.90	.93	.95
conflict		.95	.95	.95	.94	.94	.94	.93	.90	.91
automaticity		.84	.89	.85	.84	.89	.90	.85	.86	.87
Judgment	Study:	Inter-behavior coherence in ordering participants (ICC3)								
		All behaviors			Positive behaviors			Negative behaviors		
		1-all	2	3	1-all	2	3	1-all	2	3
regularity		.03	.05	.04	.10	.11	.13	.09	.12	.11
consistency		.11	.12	.08	.12	.13	.14	.16	.16	.13
immediate reward		.06	.08	.08	.17	.19	.26	.16	.17	.17
long-term reward		.06	.08	.10	.15	.20	.19	.18	.24	.30
conflict		.20	.19	.18	.27	.29	.28	.26	.18	.20
automaticity		.06	.09	.07	.11	.17	.18	.12	.13	.14

**Note.** For each SAM^2^ measure, the top half presents Cronbach’s alpha (ICC3k), which estimates the reliability of individual scores across assessment occasions (i.e., test reliability). For each study, values of alpha are shown for scores that were aggregated across all 80 behaviors, across the 40 positive behaviors, or across the 40 negative behaviors (computed with the ICC function in the R Psych package). The bottom half presents inter-behavior coherence (ICC3), which measures how much behaviors agree in their orderings of individual participants from high to low on a measure (i.e., the consistency of the test items). For each study, inter-behavior coherence is shown for all 80 behaviors, for the 40 positive behaviors, or for the 40 negative behaviors. As captured in the Spearman-Brown formula, Cronbach’s alpha increases with both behavior coherence (ICC3) and the number of behaviors aggregated in the overall measure. Both measures (ICC3k, ICC3) estimate fixed effects, assuming that the test instrument contains the same 80 behaviors across test occasions.

The Spearman-Brown formula offers insight into the values of alpha in Table 3 [23–27]. According to Spearman-Brown, alpha increases as a function of two parameters: (a) the coherence between test items (how much they intercorrelate), and (b) the number of test items. Given that the SAM^2^ aggregate measures used 40 to 80 items, the values of alpha at the top of Table 3 might be expected to be much higher than .80. The relatively low values of item coherence in the lower half of Table 3 explain the relatively low alphas at the top (the fixed-effects version of the intra-class correlation, ICC3, was used to compute these coherence values, reflecting the assumption that a constant set of behaviors is used to assess regularity across occasions [24, 25].

For the overall measure of behavior regularity across all 80 behaviors, inter-behavior coherence was around .04. These low values indicate that the 80 behaviors differed considerably in how they ordered individual participants from high to low on behavior regularity—individuals high in regularity for one behavior were low for another. In the columns of Fig 2, the widely varying patterns of regularity across individuals for different behaviors visualize the low coherence between behaviors. Most importantly, however, 80 test items are sufficient to produce acceptable alphas near .80, even when coherence is a low .04 (following Spearman-Brown).

Why might we be seeing such low coherence for the SAM^2^ HBI? According to the Situated Assessment Method, measuring a construct effectively requires assessing it in the situations where it occurs. To ensure that an aggregate measure across situations is not biased toward one kind of situation, the behavior should be assessed in a representative sample. It follows that if situations within a representative sample vary widely, then using them as a set of SAM^2^ test items is likely to exhibit low coherence. To the extent that different situations order individuals differently on the construct from high to low, coherence across a representative set of situations could be low.

One potential source of variance in habitual behaviors is whether they are positive or negative. If so, then computing coherence separately for positive vs. negative behaviors should produce greater coherence. As Table 3 illustrates, coherence for regularity increased from .04 to around .11 for both positive and negative behaviors. As a consequence, corresponding values of alpha in the top-half of Table 3 increased as well.

### Construct validity with respect to the Situated Action Cycle: Group level

If the SAM^2^ measure of behavior regularity is a valid measure of habitualness, then it should be associated with factors known to influence habitualness in the scientific literature (Fig 1). To the extent that a SAM^2^ HBI measure of regularity behaves as expected when factors such as consistency, automaticity, immediate reward, and long-term reward vary, it demonstrates construct validity.

We used multilevel mixed-effect modelling to assess whether group-level data collected with the SAM^2^ HBI exhibited this pattern of construct validity. Specifically, the SAM^2^ measure of behavior regularity was regressed onto the factors in the Situated Action Cycle common across studies in Fig 1. All models included all main effects, two-way interactions, and three-way interactions, together with random intercepts for participants and behaviors, and with relevant random slopes. S1 File provides a detailed account of the regression pipeline, along with detailed regression results for findings reported here and later.

**Fig 4:**
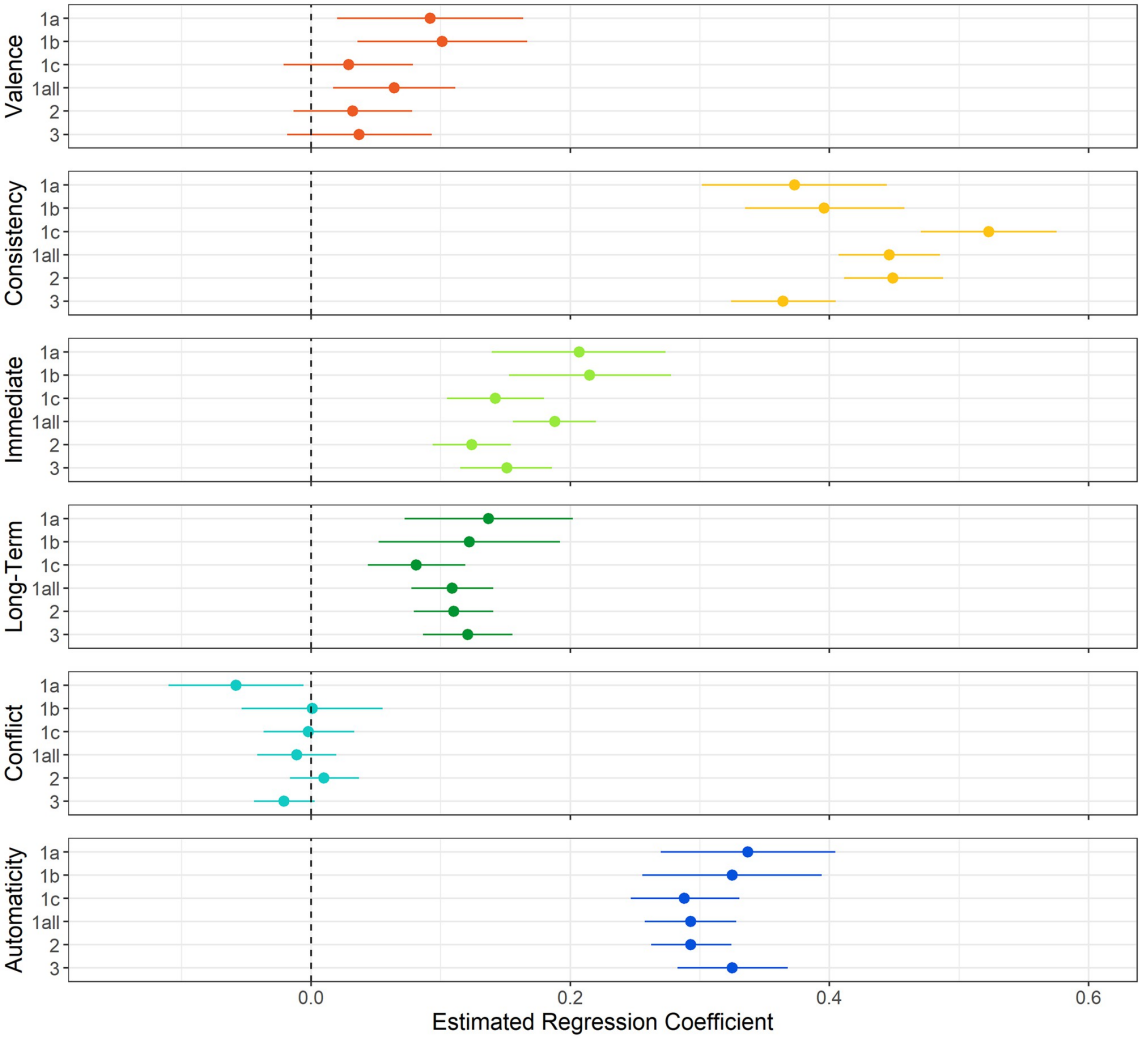
Estimated regression coefficients and their 95% confidence intervals at the group level for the prediction of behavior regularity across Studies 1-all, 2, and 3, including predictors for a priori valence, consistency, immediate reward, long-term reward, conflict, and automaticity. *Note*. To demonstrate replicability, even with small sample sizes, Studies 1a, 1b, and 1c are shown, along with these three studies combined for Study 1-all. All coefficients are standardized and were established in regression models that included random intercepts, random slopes, and interactions (all two- and three-way). S1 File provides details of the regression analysis procedure and results.

Fig 4 presents the standardized regression coefficients from these analyses with their 95% confidence intervals. By virtue of being standardized, these coefficients provide a measure of effect size. To demonstrate robustness of the coefficients across studies (and especially for small sample sizes), Fig 4 includes coefficients for the three parts of Study 1-all. The value for each coefficient in Fig 4 was obtained from an analysis where random slopes were included for the respective fixed effect, thereby conservatively assessing its generalizability across participants and behaviors.

As Fig 4 illustrates, a common pattern emerged across studies, demonstrating that the SAM^2^ HBI produces highly replicable results. The same pattern of results occurred regardless of whether sample size was 31 (Studies 1a and 1b) or 199 (Study 2), regardless of whether data were collected in the lab (Studies 1a and 1b) or online (Studies 1c, 2, and 3), regardless of whether judgment blocks were fixed (Studies 1-all and 2) or randomized (Study 3).

Most importantly, these results establish construct validity for the SAM^2^ HBI. Consistent with the habits literature, factors associated with conditioning—consistency and automaticity—were most strongly related to behavior regularity. A behavior became more regular as it was performed more automatically in increasingly consistent situations. Additionally, immediate and long-term reward were also associated with behavior regularity, albeit to a weaker extent than consistency and automaticity. Unexpectedly, conflict was not consistently related to regularity, with most standardized coefficients clustering around 0. Although valence exhibited a weak positive relation with regularity, its most potent effects emerged through interactions with self-control and neuroticism, addressed later.

Finally, these group-level analyses generally explained 65% to 70% of the total variance in behavior regularity (S1 File provides detailed results). This explanatory success demonstrates content validity of the SAM^2^ HBI: Its measures across the Situated Action Cycle are sufficiently thorough and complete to explain the bulk of the variance in habitual behavior.

### Construct validity with respect to the Situated Action Cycle: Individual level

As we will see both here and in the remaining sections, results at the individual level further demonstrate construct validity for the SAM^2^ HBI. We first address whether each individual’s judgments of consistency, automaticity, immediate reward, long-term reward, and conflict tended to exhibit predicted relations with habitual behavior observed in the literature. As participants judged each of these factors, did their judgments covary with their judgments of behavior regularity? Notably, participants did not appear to be explicitly aware of how the various predictors covary with behavior regularity, given their general inability to report these relationships accurately (see supporting evidence from an analysis of awareness judgments in S1 File). Thus, if these factors from the Situated Action Cycle covary with behavior regularity, they are likely to instead reflect implicit structure across habitual behaviors that the SAM^2^ HBI captures.

**Fig 5:**
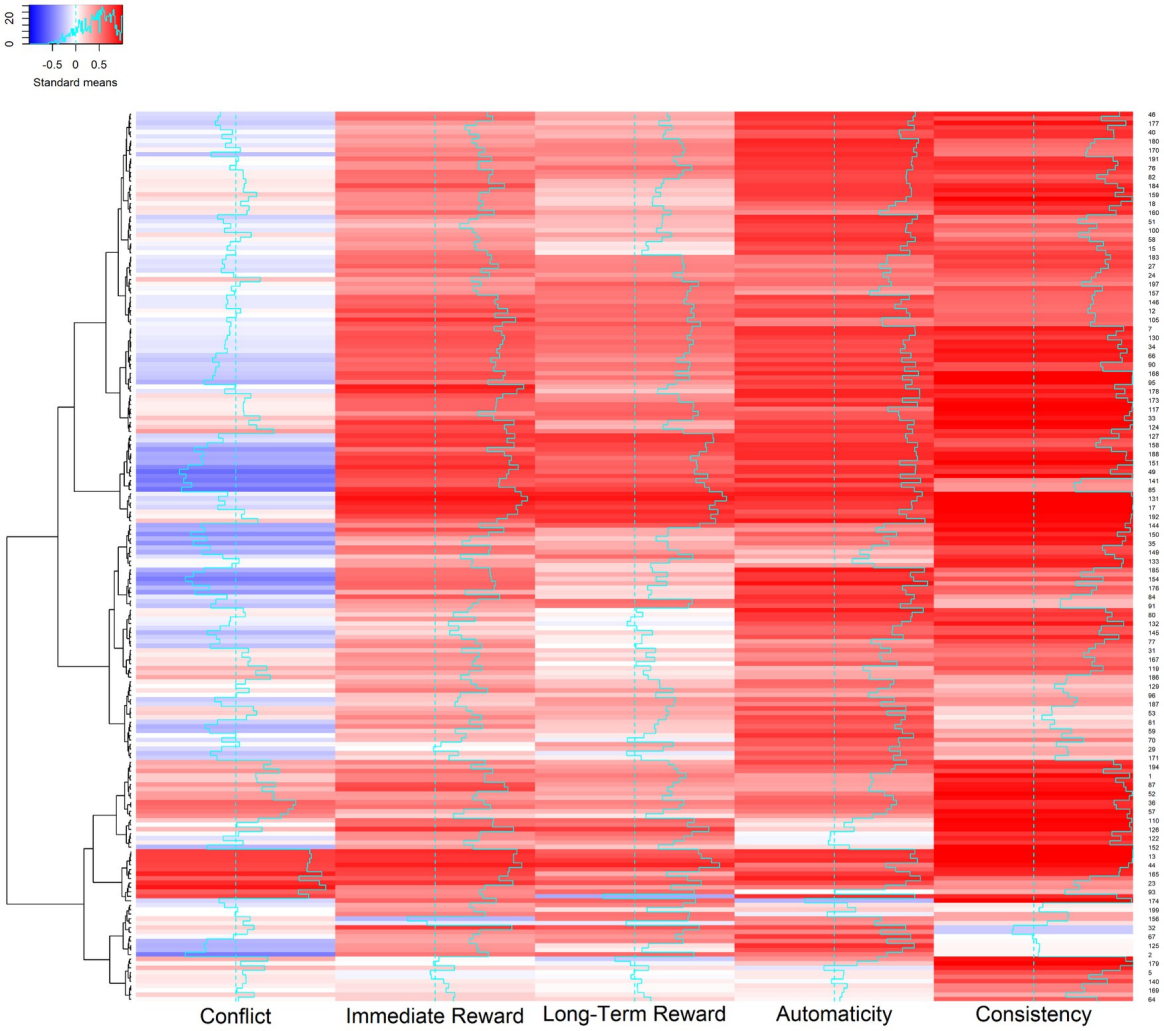
A heatmap that visualizes correlations between behavior regularity and individual factors from the Situated Action Cycle (conflict, immediate reward, long-term reward, automaticity, and consistency). *Note*. The six correlations for each of the 199 participants in Study 2 appear in a single row. As a cell becomes increasingly red, the correlation was increasingly positive. As a cell becomes increasingly blue, the correlation was increasingly negative. As a cell becomes increasingly white, the correlation increasingly approached 0. On the left, a hierarchical clustering dendrogram establishes groups of participants having similar prediction vectors (from hierarchical clustering with the Ward D measure). Analogous heatmaps for Studies 1-all and 3 can be found in S1 File. Table 4 summarizes the correlations in all three studies.

Each row of the heat map in Fig 5 visualizes the correlations between behavior regularity and the five predictive factors from the Situated Action Cycle for each of the 199 participants in Study 2 (S1 File presents analogous heatmaps for Studies 1-all and 3). As a cell becomes redder, the correlation approaches +1; as it becomes bluer, it approaches -1; as it becomes whiter, it approaches 0. Table 4 summarizes the correlations in Fig 5, presenting the 25^th^, 50^th^, and 75^th^ quartiles for each correlation across participants in Studies 1-all, 2, and 3.

**Table 4:** Medians (interquartile ranges) of the individual correlations in Fig 5 between behavior regularity and factors from the Situated Action Cycle (Consistency, immediate reward, long-term reward, conflict, and automaticity).

Study	Consistency	Immediate Reward	Long-Term Reward	Conflict	Automaticity
1-all	.75 (.57—.87)	.49 (.38—.58)	.37 (.20—.47)	-.08 (-.23—.08)	.70 (.60—.78)
2	.72 (.53—.89)	.49 (.37—.63)	.39 (.21—.56)	-.02 (-.18—.13)	.66 (.48—.77)
3	.75 (.61—.84)	.40 (.25—.53)	.31 (.10—.49)	-.01 (-.16—.12)	.74 (.55—.81)

**Note**. All median values for consistency, immediate reward, long-term reward, and automaticity in Studies 1-all, 2, and 3 differed significantly from 0 in the predicted positive direction (using Mann-Whitney tests, with all *p* < 2.2e-16, one-tailed). None of the median values for conflict in Studies 1-all, 2, and 3 differed significantly from 0.

As both Fig 5 and Table 4 illustrate, the general pattern for individual prediction conformed to the pattern at the group level. Again, consistency and automaticity were most strongly related to behavior regularity, exhibiting significant median correlations around .75 and .70, respectively. Again, immediate and long-term reward were also related to regularity significantly but to a lesser extent, exhibiting median correlations around .45 and .35, respectively. Again, conflict appeared relatively unrelated to regularity. These results further establish construct validity for the SAM^2^ HBI. Not only does it capture well-established relations between constructs at the group level, it also captures them at the individual level.

We hasten to add that considerable individual differences were nevertheless present. In Fig 5, behavior regularity failed to correlate highly with consistency or automaticity for quite a few individuals. The absence of correlations for some individuals is even more prevalent for reward, especially for long-term reward. Furthermore, major differences in individual prediction vectors differentiate clusters of related individuals, as captured by the hierarchical clustering dendrogram on the left.

### Content validity in individual regressions

Earlier we saw considerable individual differences in judgments of regularity (ICC2 values in Table 2), consistent with a large individual by behavior interaction (Fig 2). Of interest here is whether these large individual differences simply reflected noise or whether they were systemic. If they were systematic, then factors from the Situated Action Cycle in Fig 1 should generally explain high levels of variance in the regularity judgments of individual participants. In other words, the SAM^2^ HBI should again illustrate a high level of content validity.

To assess this prediction, a simple linear regression was performed for each participant in Studies 1-all, 2, and 3 on their individually standardized data (*n* = 128, 199, 115, respectively). Each regression only modeled fixed effects, given that no random effects were logically possible in the individual models. Each regression included behavior regularity as the dependent variable, along with consistency, immediate reward, long-term reward, conflict, and automaticity as predictors. Only main effects were modeled with no interactions. The goal of these simple multiple regressions was to construct a prediction profile of behavior regularity for each individual.

**Fig 6:**
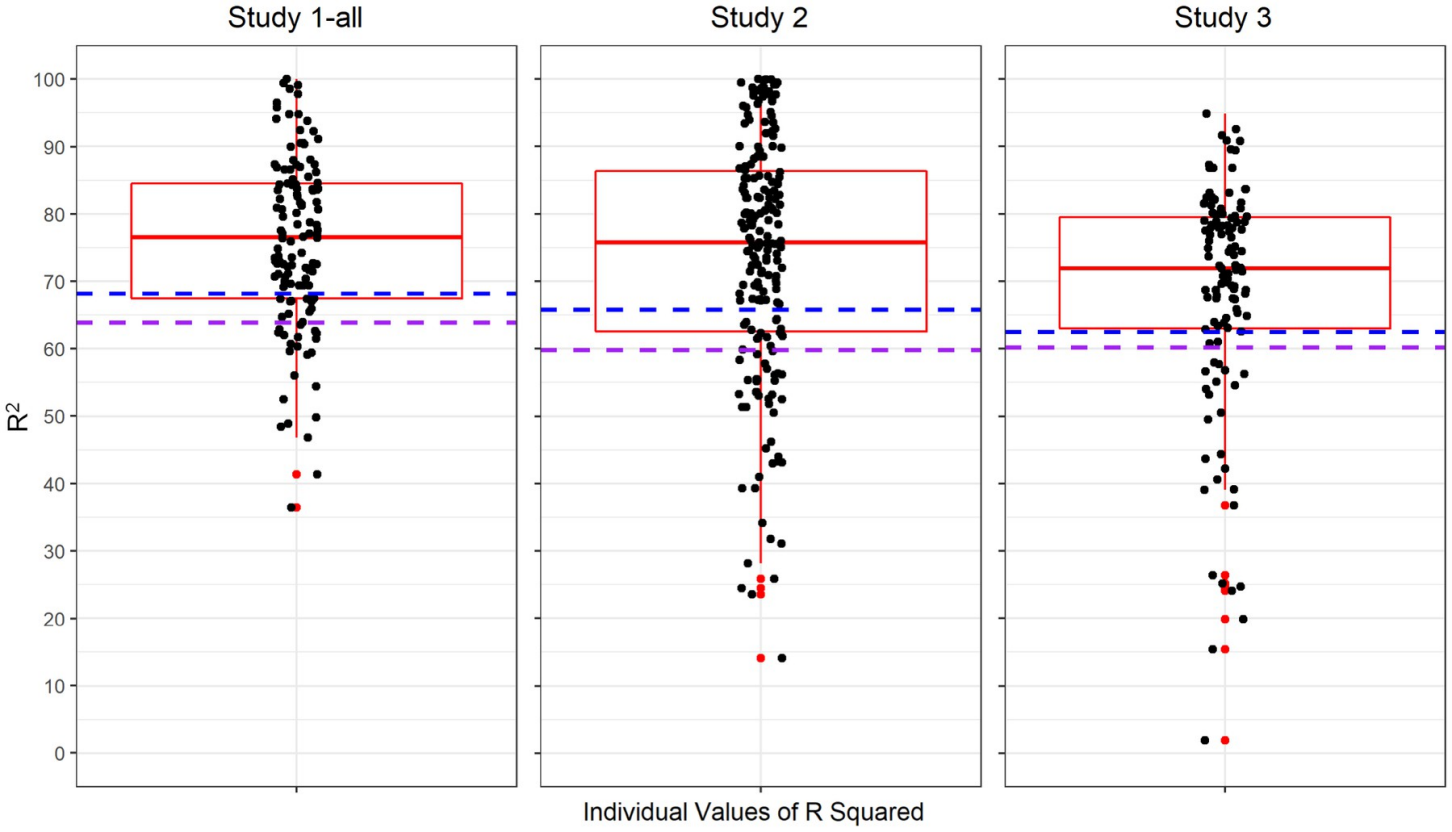
*R*^2^ plotted as percentages for the individual regressions in Studies 1-all, 2, and 3 (in percentage points). *Note*. Each dot is the *R*^2^ for a participant’s individual regression, where each regression predicts the individual’s judgments of behavior regularity from their judgments of consistency, immediate reward, long-term reward, conflict, and automaticity (with no interactions or random effects). A box and whisker plot shows the median and inter-quartile range in each study. The lower dashed purple line shows *R*^2^ for the same main-effects-only individual model with no interactions or random effects when run at the group level. The upper dashed blue line shows *R*^2^ for Model 1 at the group level with random intercepts and interactions from Table 5. Each median *R*^2^ for the individual regressions in Studies 1-all, 2, and 3 differed significantly from the respective main-effects-only group model in the predicted positive direction (using Mann-Whitney tests, all *p* < 1.0e-6, one-tailed). Each median also differed significantly from the respective random-effects-plus-interactions group model in the predicted positive direction (using Mann-Whitney tests, all *p* < 1.0e-4, one-tailed).

Fig 6 presents the variance explained (*R*^2^) in the individual regressions, with the median *R*^2^ being 77% in Study 1-all, 75% in Study 2, and 72% in Study 3 (with *R*^2^ expressed as a percentage). The *R*^2^ for the same main-effects-only individual model with no random effects or interactions when run at the group level is plotted as the lower dashed purple line. The *R*^2^ for a group-level model with random intercepts and interactions is plotted as the upper dashed blue line. As can be seen, the median *R*^2^ for the individual models was significantly higher than the variance explained by both group-level models in every study.

**Table 5:** The 80 behaviors ordered by the composite measure of habitualness (averaged across Study 2 participants), with their composite reward values shown as well.

Behavior number* / name		Composite measure		Behavior number* / name		Composite measure	
		Habitualness	Reward			Habitualness	Reward
28	Say ’please’ and ’thank you’	87.25	3.57	42	Take study breaks	58.07	1.87
39	Check my phone multiple times a day	85.03	1.31	54	Pick my spots and/ or scabs	57.64	-0.99
34	Charge my devices	83.57	2.94	08	Drink soft drinks	57.29	0.11
50	Cover my mouth when sneezing, coughing or yawning	81.25	2.79	02	Eat healthy snacks	57.19	2.63
27	Hold doors open for others	78.51	2.58	24	Ignore my own needs	56.65	-1.55
49	Shower every day	74.33	3.17	71	Buy brand name products	56.24	0.47
38	Use my phone as a social crutch	72.77	1.03	79	Leave plug sockets switched on	56.24	-1.47
57	Wash my clothes	72.66	2.77	43	Set goals before engaging in a task	55.84	2.34
03	Eat vegetables	72.23	3.08	67	Use shopping lists	54.95	1.73
74	Recycle	71.80	2.64	61	Allow messes to build up in my work area	53.38	-1.55
22	Worry	71.56	-1.82	33	Make back-up copies of important documents and files	53.23	2.01
37	Spend a large amount of time on social media	71.15	0.25	52	Go to sleep and wake up at the same times	51.58	1.69
75	Reuse carrier bags	70.82	2.50	12	Take standing and walking breaks when sitting for long periods of time	51.12	2.09
73	Turn off lights when leaving a room	70.24	2.17	15	Reward myself with food and/or drink after exercise	51.08	0.09
68	Shop for groceries	69.26	2.41	09	Exercise	50.93	2.18
23	Criticise myself	69.24	-1.13	16	Use the lift instead of taking the stairs	50.85	0.14
25	Maintain contact with family	68.48	2.74	19	Express my emotions constructively	50.46	1.92
40	Use my phone whilst on the toilet	68.28	0.77	29	Use bad language in public	50.42	-1.17
45	Procrastinate	67.90	-0.33	06	Eat dessert	50.04	0.27
51	Brush my teeth twice a day	67.48	2.95	70	Spend to make myself feel better	49.43	0.18
58	Put things back after I have finished using them	66.42	2.15	72	Make impulsive purchases	48.36	-0.22
48	Multi-task during work	66.24	1.31	07	Eat fast foods	46.84	-0.21
41	Study for my course(s)	66.13	2.51	53	Pick my nose	45.56	-0.82
60	Clean my residence	65.60	2.75	05	Drink alcohol	44.22	-0.55
46	Work whilst watching TV or listening to music	64.64	0.85	80	Throw away food	43.99	-1.87
01	Eat fruit	63.45	3.07	69	Dip into funds I have set aside	43.73	-0.84
59	Empty the bins	63.40	2.02	56	Bite my nails	41.06	-2.00
17	Take time to relax	63.10	2.90	31	Pay little attention to others when they are talking	40.78	-1.61
04	Check food labels before making purchases	62.11	1.94	14	Avoid long walks	40.71	-1.00
13	Be sedentary for long periods of time	61.73	-0.72	66	Buy from charity and/ or second-hand shops	38.54	0.74
78	Buy new condition items	61.28	1.72	62	Ignore stains and spills	36.29	-1.57
63	Leave dishes to wash later	60.90	-0.51	30	Interrupt others	35.64	-2.00
10	Walk or bike when possible	60.69	2.40	11	Participate in sports activities and clubs	34.79	1.08
64	Leave clothes lying around	60.62	-1.03	32	Make myself the centre of conversation	34.77	-1.46
26	Maintain contact with friends	60.55	2.90	55	Chew on pencils and/ or pens	33.04	-2.31
44	Pack what I need the night before	60.07	2.06	35	Limit the amount of time each day I spend using technology	31.90	0.28
65	Budget	59.84	2.40	36	Restrict my use of technology before sleep	30.86	0.37
18	Do at least one thing a day that I enjoy and look forward to	58.79	2.72	47	Skip lectures	29.28	-1.76
20	View challenges with a positive attitude	58.47	2.65	21	Use substances to relax	29.22	-1.93
76	Use reusable cups	58.46	1.34	77	Litter	20.05	-3.09

* From Table 1

These results for the individual regressions indicate that the relatively low interrater agreement in Table 2 did not reflect noise but instead reflected systematic individual differences. Specifically, high *R*^2^ values for the individual regressions show that the SAM^2^ judgments within individuals tended to be highly systematic. As a result, low agreement between individuals for behavior regularity reflected large differences in their systematic judgments of it. These results demonstrate the content validity of the SAM^2^ HBI at the individual level, explaining even more variance in behavior regularity than at the group level.

### Construct validity with respect to self-control and neuroticism

As described earlier, the literature on habitual behavior has established interactions between personality measures and habit valence. As an individual’s self-control increases, they tend to perform positive behaviors more and negative ones less (a self-control × valence interaction). Conversely, as an individual’s neuroticism increases, they tend to perform negative behaviors more and positive ones less (a neuroticism × valence interaction). If the SAM^2^ measure of behavior regularity is a valid measure of habitualness, it should capture these interactions.

**Fig 7:**
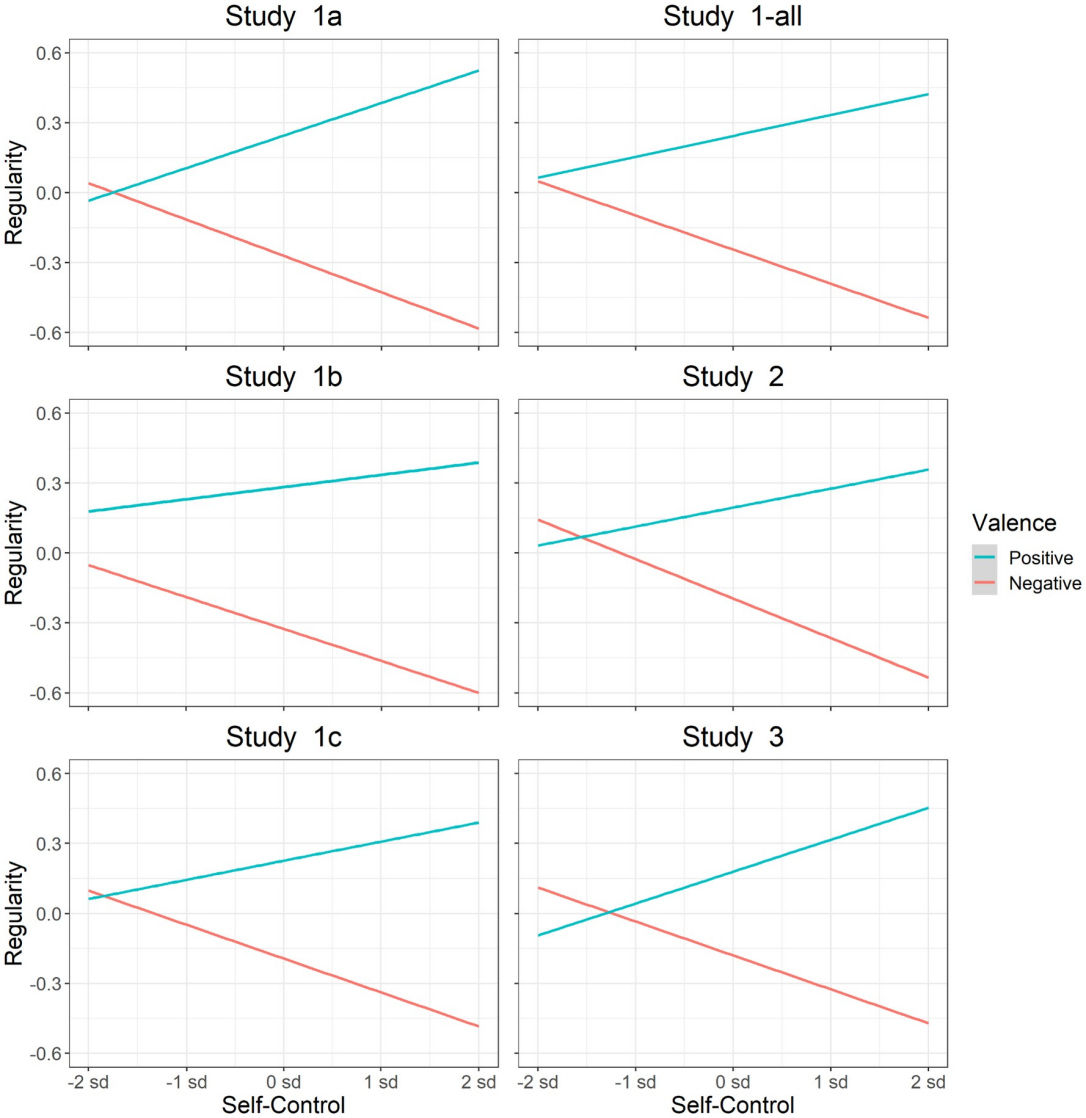
The interaction of behavior valence with self-control across Studies 1-all, 2, and 3. *Note*. Each interaction modeled here was established in a mixed-effect regression analysis that predicted behavior regularity as a function of only valence and self-control (with all three variables standardized prior to analysis). Valence was an a priori variable that contrasted positive versus negative behaviors (Table 1). S1 File provides details of the regression analysis procedure and results.

**Fig 8:**
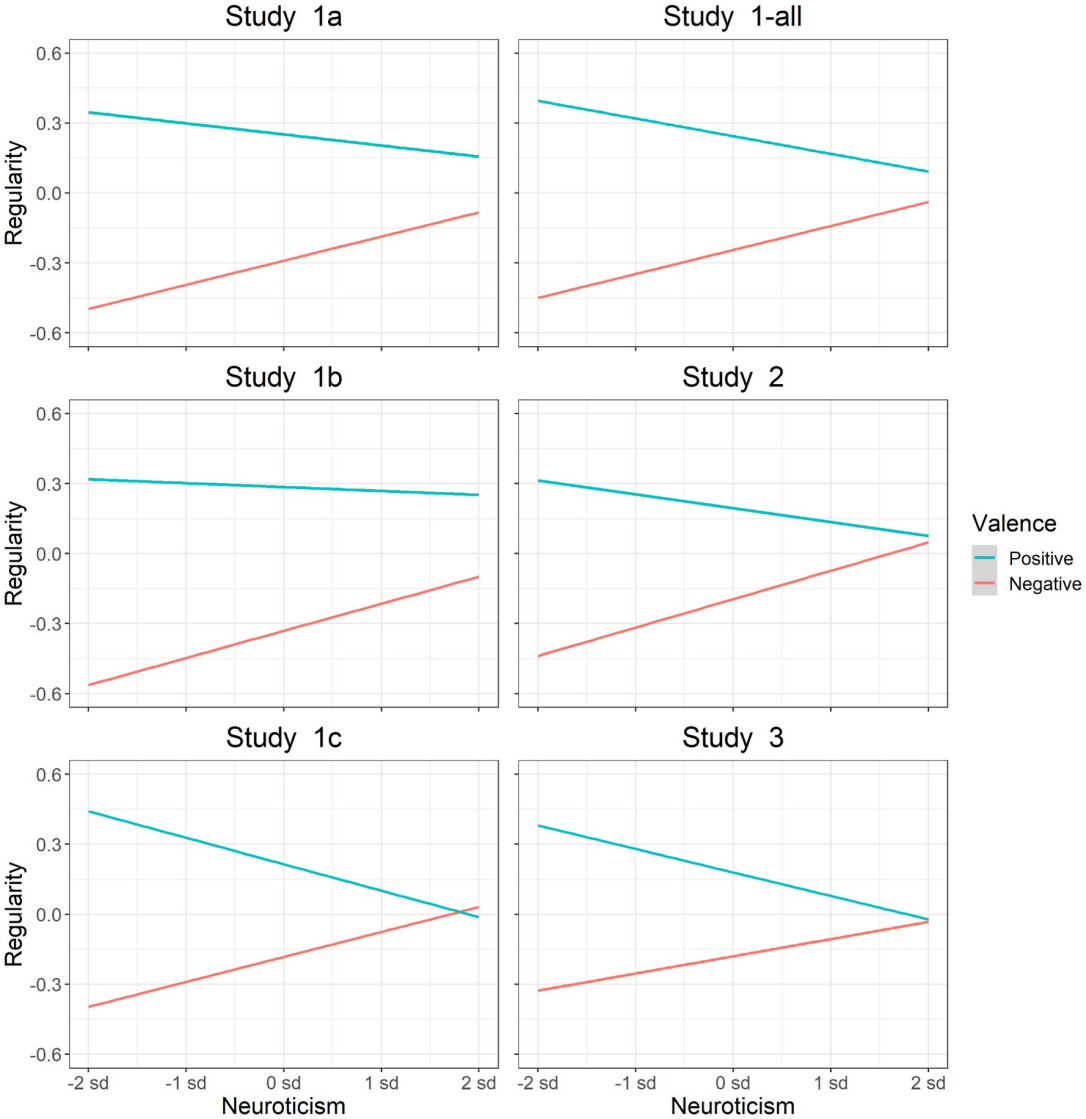
The interaction of behavior valence with neuroticism across Studies 1-all, 2, and 3. *Note*. Each interaction modeled here was established in a mixed-effect regression analysis that predicted behavior regularity as a function of only valence and neuroticism (with all three variables standardized prior to analysis). Valence was an a priori variable that contrasted positive versus negative behaviors (Table 1). S1 File provides details of the regression analysis procedure and results.

Figs 7 and 8 present these interactions across studies, including the three sub-studies of Study 1-all to demonstrate robustness in small samples. As Fig 7 illustrates, self-control was associated with increasing regularity of positive behaviors and decreasing regularity of negative behaviors across studies. As Fig 8 illustrates, neuroticism was associated with decreasing regularity of positive behaviors and increasing regularity of negative behaviors. S1 File presents detailed analyses of these interactions, all remaining robust when random slopes were included for participants and behaviors, demonstrating their generalizability.

These findings further establish construct validity for the SAM^2^ HBI. Consistent with previous literature on relations between personality and habit valence, self-control and neuroticism strongly affected the regularity of positive and negative behaviors in opposite manners.

### Using the Situated Action Cycle to understand self-control and neuroticism

As discussed earlier, viewing self-control and neuroticism as internal trait mechanisms that cause behavior offers limited theoretical understanding of these behavioral dispositions. To understand self-control and neuroticism, it is instead necessary to develop accounts of the underlying cognitive and affective processes as they interact with situations to produce these stable tendencies in behavior [38–42, 44, 45, 47]. As will see in these next analyses, the SAM^2^ framework offers a novel and effective approach for establishing such accounts.

Of interest in these analyses was whether factors from the Situated Action Cycle for habitual behavior in Fig 1—consistency, automaticity, immediate reward, long-term reward, and conflict—explain variance in self-control and neuroticism. To see this, first consider the self-control × valence interactions in Fig 7, which resulted from regressing behavior regularity onto only self-control, valence, and their two-way interaction (including random intercepts and slopes as described in S1 File). If factors from the Situated Action Cycle for habitual behavior in Fig 1 underlie self-control, then adding all these factors into the initial regression models should eliminate self-control’s interactions with valence. If these factors don’t underlie self-control, then the self-control × valence interactions should remain.

When all factors from the Situated Action Cycle for habitual behavior were added to the original regression models, the self-control × valence interactions in Fig 7 completely disappeared across studies. When the same procedure was applied to the neuroticism × valence interactions in Fig 8, they, too, disappeared completely. These results demonstrate that factors from the Situated Action Cycle underlie the behavioral tendencies associated with self-control and neuroticism. S1 File presents these analyses in detail.

We next used stepwise regression to identify the *specific* SAM^2^ factor(s) from the Situated Action Cycle that explained variance in these interactions. At each regression step, all factors were entered, one at a time, to assess how much adding each alone decreased the estimated regression coefficient for the interaction of interest. After each step, we added the (remaining) factor that decreased the coefficient the most, continuing until all factors had been added. S1 File provides the details of these analyses.

**Fig 9:**
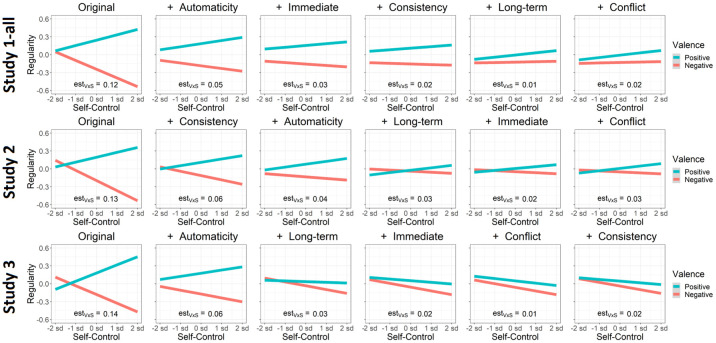
Results from stepwise regression to establish the SAM^2^ predictors that explained variance in the valence × self-control interaction in Studies 1-all, 2, and 3 (Fig 7). *Note*. In the original analyses (left column), behavior regularity was regressed onto only valence and self-control in Model 2 from the analysis pipeline to test the valence × self-control interaction maximally. The five primary SAM^2^ predictors (Fig 1) were then added one at a time into the original regression to assess how much each alone decreased the coefficient for the valence × self-control interaction (est_V×S_). The predictor that decreased est_V×S_ the most is shown in the second column, along with a plot of the resulting interaction and the new value of est_V×S_. In four further iterations of the stepwise process, the remaining SAM^2^ predictors were again added one by one to identify the predictor that next decreased the est_V×S_ interaction the most. These results are shown in the third, fourth, fifth, and sixth columns. S1 File provides details of the regression analysis procedure and results.

**Fig 10:**
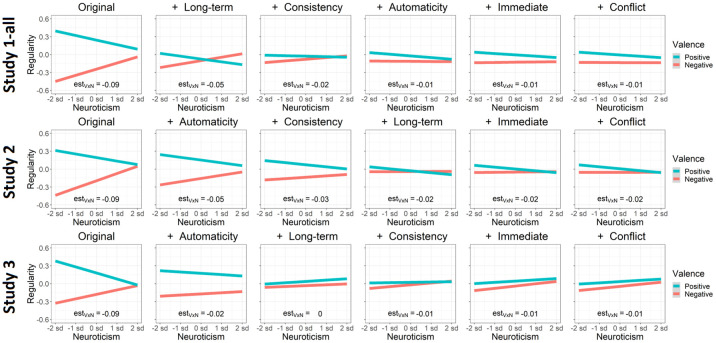
Results from stepwise regression to establish the SAM^2^ predictors that explained variance in the valence × neuroticism interaction in Studies 1-all, 2, and 3 (Fig 8). *Note*. In the original analyses (left column), behavior regularity was regressed onto only valence and neuroticism in Model 2 from the analysis pipeline to test the valence × neuroticism interaction maximally. The five primary SAM^2^ predictors (Fig 1) were then added one at a time into the original regression to assess how much each alone decreased the coefficient for the valence × neuroticism interaction (est_V×N_). The predictor that decreased est_V×N_ the most is shown in the second column, along with a plot of the resulting interaction and the new value of est_V×N_. In four further iterations of the stepwise process, the remaining SAM^2^ predictors were again added one by one to identify the predictor that next decreased the est_V×N_ interaction the most. These results are shown in the third, fourth, fifth, and sixth columns. S1 File provides details of the regression analysis procedure and results.

Fig 9 presents results from the stepwise process for the self-control × valence interactions in Studies 1-all, 2, and 3. Across studies, automaticity, consistency, long-term reward, and immediate reward explained the increased regularity of positive behaviors and the decreased regularity of negative behaviors associated with self-control. Fig 10 presents the analogous results for the neuroticism × valence interactions. Across studies, automaticity, long-term reward, and consistency explained the decreased performance of positive behaviors and the increased performance of negative behaviors associated with neuroticism.

These analyses further demonstrate construct validity of the SAM^2^ HBI. Not only does this instrument capture well-established relations between behavior regularity and relevant personality measures, it also provides insights into factors from the Situated Action Cycle that underlie the associated behavioral dispositions.

### Establishing habitual behaviors

#### The dimension of habitualness

We next illustrate how the SAM^2^ HBI can be used to establish habitual behaviors for both a group and an individual. As discussed earlier, considerable disagreement surrounds the criteria that establish a behavior as a habit. For example, a habit could be a behavior produced automatically or a behavior preceded by automatic cognition. A habit could be a behavior that is performed frequently in consistent situations. A habit could be a ballistic behavior not modulated by reward. A habit could be a behavior performed regularly as conditions allow, regardless of automaticity, consistency, and reward.

To address this issue, we developed a composite measure of habitualness that combined regularity, consistency, and automaticity (i.e., for each behavior in Table 1, the average of an individual’s judgements of regularity, consistency, and automaticity). Essentially, this measure assumes that as behavior becomes increasingly regular, consistent, and automatic, it becomes increasingly habitual in a continuous manner.

#### Establishing habitualness at the group level

Table 5 presents the 80 behaviors from Table 1 ordered by composite habitualness averaged across the 199 participants in Study 2. The 40 most habitual behaviors appear on the left, and the 40 least habitual behaviors appear on the right. Examining the most and least habitual behaviors confers face validity on the composite measure of habitualness. The most habitual behaviors strike us as intuitively likely to be highly habitual (e.g., saying please and thank you, check my phone, shower every day, worry). In contrast, the least habitual behaviors strike us as intuitively likely to be less habitual (e.g., chew on pencils, skip lectures, use substances to relax, litter).

**Fig 11:**
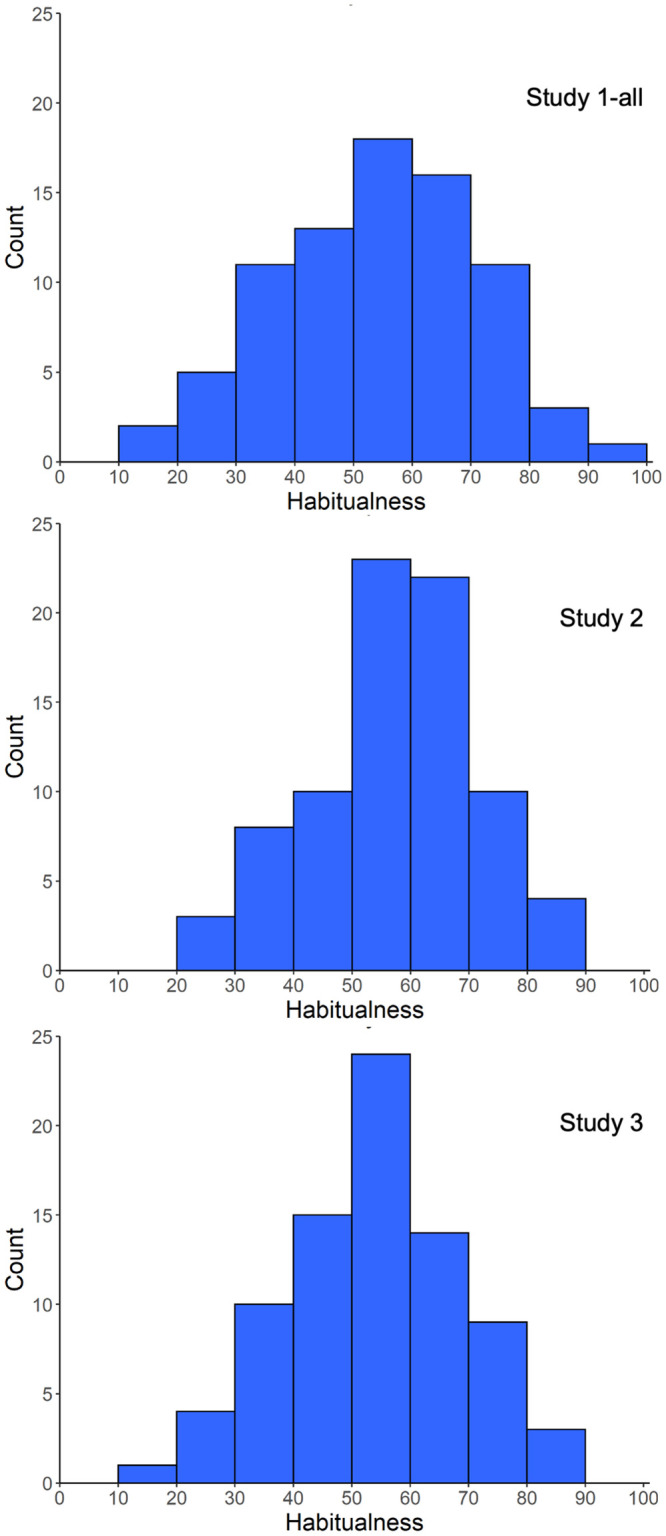
Histograms showing counts of the 80 behaviors in Table 1 on their compositive values for habitualness averaged across participants in Studies 1-all, 2, and 3.

For each study, Fig 11 presents a histogram for the 80 behaviors as a function of composite habitualness. Strong similarity in the distributions holds across studies, with maximal density in the 50 to 60 range. An interesting question is where one might establish a threshold on this distribution for classifying a behavior as a habit. Because all measures going into composite habitualness used a 0 to 100 likelihood scale, a value of 50 can be viewed as a behavior that was performed regularly, automatically, and in consistent situations about 50% of the time. What should the threshold of this measure be for calling a behavior a habit? If the threshold were set at 80%, very few behaviors would be habits at the group level. If the threshold were set around 50%, many habits would be performed regularly and automatically in consistent situations 50% of the time. Should these be called habits? Many are behaviors that people might well consider habitual, such as *take study breaks*, *eat healthy snacks*, and *use shopping lists*.

An alternative way of viewing these plots is to conceptualize habitualness as a dimension that varies continuously across behaviors. From this perspective, attempting to establish a category of bona fide habits is not likely to be easy, successful, or theoretically meaningful. Similarly, instead of asking how long it takes to learn a habit [135], it perhaps is more useful to establish how the habitualness of a behavior grows continuously over time.

#### Establishing habitualness at the individual level

As we saw earlier in Fig 2 and Table 2, considerable differences occurred in how regularly individuals perform the 80 behaviors in Table 1. Thus, the values of composite habitualness for specific behaviors are likely to vary considerably across individuals as well.

We first addressed this issue as follows. For each study, we computed an intraclass correlation that established how much the composite measure of habitualness varied between individuals [ICC2; 24, 25]. Analogous to Table 2, these intraclass correlations assessed interrater agreement in habitualness for the 80 behaviors. For Studies 1-all, 2, and 3, these values were .31, .26, and .27, respectively, indicating large individual differences in habitualness. Because the ICC2 estimates random effects, these values generalize to other individuals in the population.

To further document individual differences in habitualness, we established how many of the 80 behaviors in Table 1 were *the first* most habitual behavior for at least one individual. In Studies 1-all, 2, and 3, this number was 35, 53, and 39 out of 80, respectively. We then assessed the number of behaviors that fell into the *top five* most habitual behaviors for at least one individual. In Studies 1-all, 2, and 3, this number was 65, 73, and 70 out of 80, respectively. Earlier we proposed that the 80 behaviors in Table 1 could potentially be highly habitual for at least some individuals. These results support that claim.

**Fig 12:**
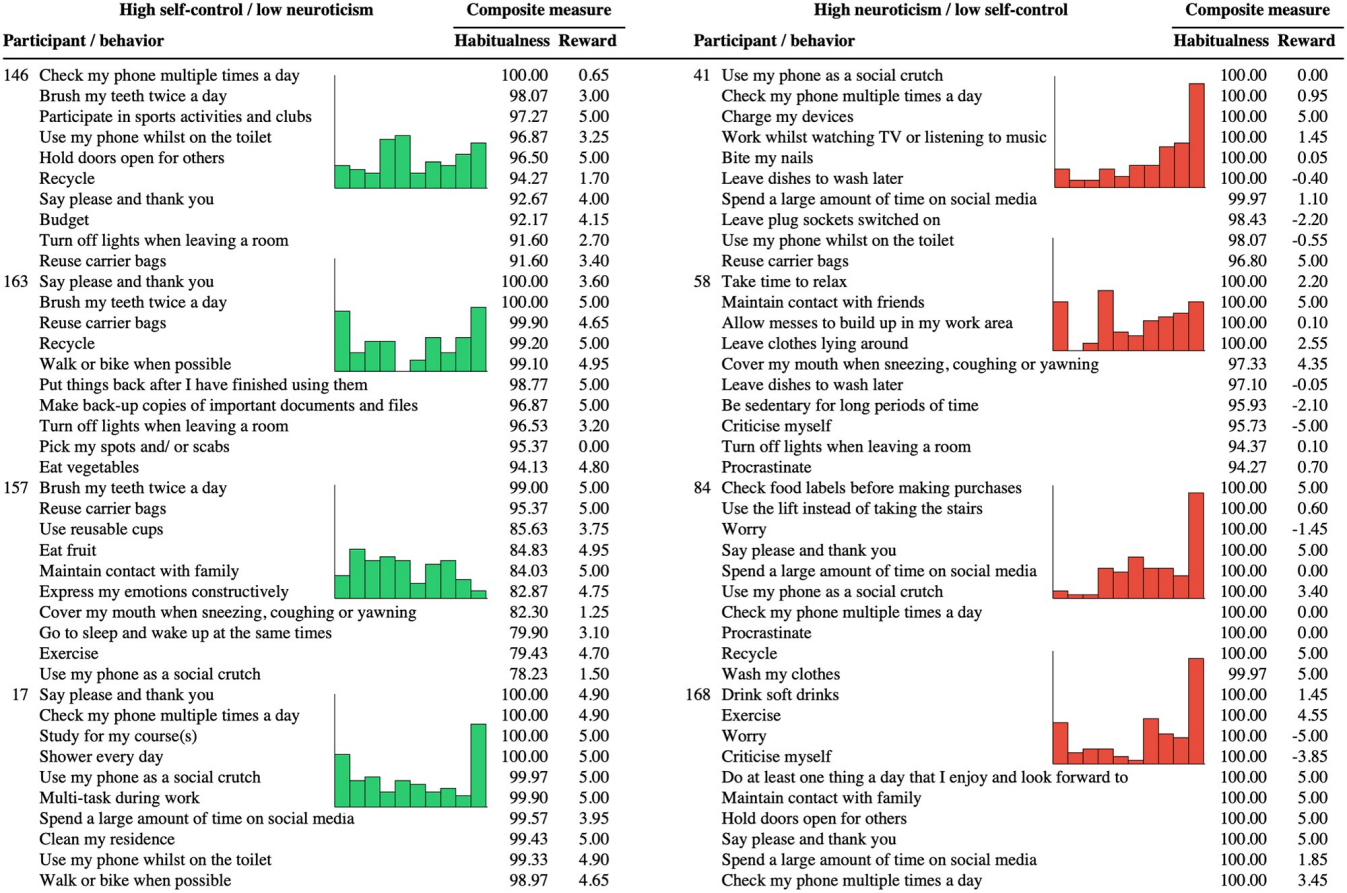
The top ten most habitual behaviors from four Study 2 participants who were high in self-control and low in neuroticism (left), and the top ten most habitual behaviors from four Study 2 participants who were high in neuroticism and low in self-control (right). *Note*. Composite values of habitualness and reward are shown for each behavior. Each inset Fig shows the habitualness histogram for a participant’s 80 behaviors, with the same axes as the histograms in Fig 11.

Fig 12 further illustrates these large individual differences, showing the top ten most habitual behaviors for eight individuals in Study 2. Whereas the four individuals on the left were relatively high in self-control and low in neuroticism, the four individuals on the right were relatively low in self-control and high in neuroticism. As can be seen, considerable variability existed in the top ten most habitual behaviors for even individuals with the same personality profile. Even more striking differences occurred between profiles.

For each individual, an inset figure plots the histogram of their 80 behaviors for the measure of composite habitualness (with the axes being the same as the group distributions in Fig 11). As can be seen, the individual distributions varied widely, departing considerably from the group distributions. Again, large individual differences occurred, and it is not clear how one might motivate a consistent habit threshold across them in a principled manner.

### Establishing the relation between habitualness and reward

Finally, we illustrate how the SAM^2^ HBI can be used to establish the relation between habitualness and reward at both the group and individual levels. As described earlier, some researchers propose that habits run ballistically, not modulated by reward. In contrast, other researchers make compelling empirical and theoretical cases that reward modulates the implementation of habitual behavior.

To establish the relation between habitualness and reward, we constructed a composite measure of reward from the SAM^2^ measures for immediate and long-term reward: the average of an individual’s judgements of immediate and long-term reward for each behavior in Table 1. Table 5 presents the compositive values averaged across participants in Study 2. Fig 12 presents the compositive values for the top ten most habitual behaviors of eight participants in Study 2.

#### Assessing the relation between habitualness and reward at the group level

**Fig 13:**
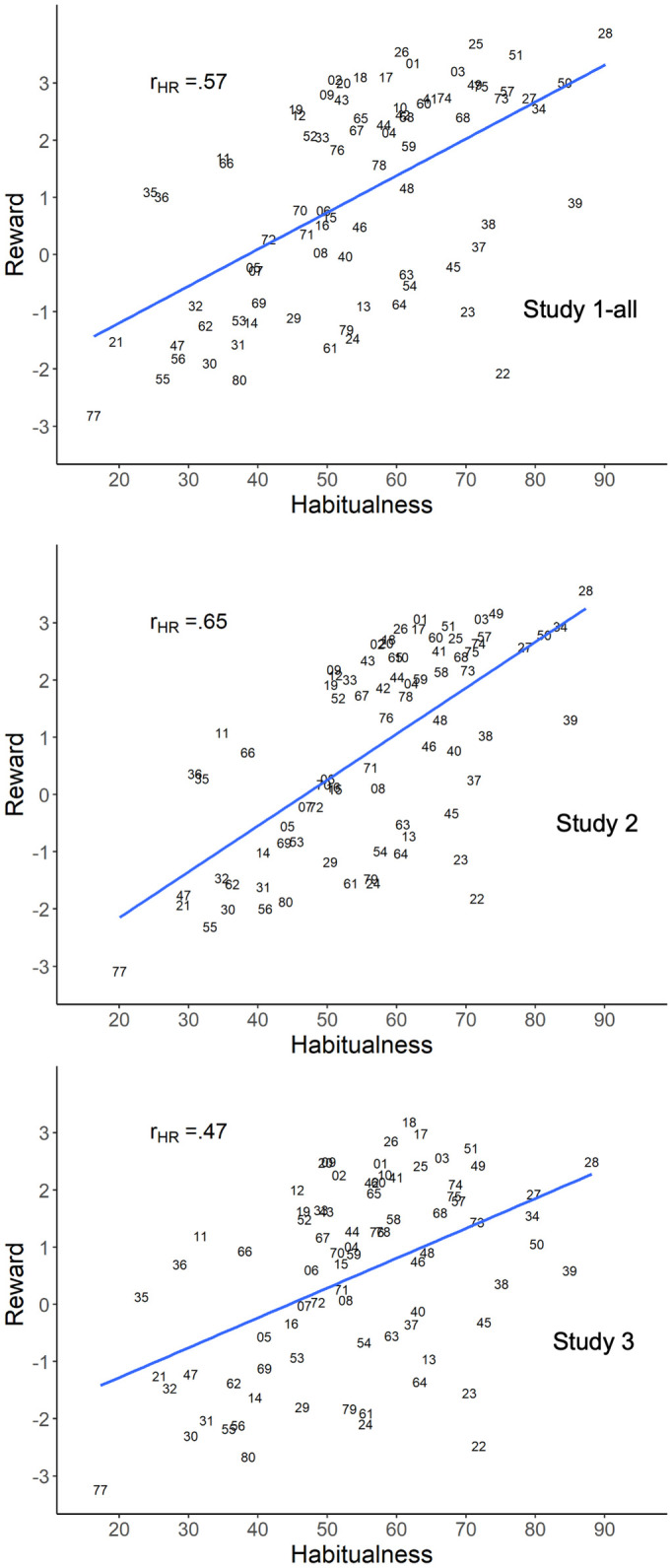
Scatterplots of the 80 behaviors in Table 1 on their compositive values for reward and habitualness averaged across participants in Studies 1-all, 2, and 3. *Note*. The behavior numbers correspond to those in Table 1. r_HR_ is the correlation between the two composite means across behaviors.

Fig 13 presents scatterplots for the composite measures of habitualness and reward across the 80 behaviors at the group level (averaged across participants within Studies 1-all, 2, and 3). The point in a scatterplot for each behavior is designated by its number in Tables 1 and 5. As can be seen, habitualness and reward were positively related across studies, exhibiting correlations of .57, .65., and .47 for Studies 1-all, 2, and 3, respectively (every *p* < .0001, two-tailed). As a behavior became increasingly habitual, it became increasingly rewarding as well.

Examining specific behaviors in Fig 13 is informative. Across studies, *Saying please and thank you* (behavior 28) was simultaneously most habitual and most rewarding. Conversely, at the other end, *littering* (behavior 77) was simultaneously least habitual and least rewarding. Other interesting cases include *worry* (behavior 22)—a highly habitual behavior low in reward—and *participate in sports activities and clubs* (behavior 11)—a relatively non-habitual behavior high in reward. The position of a specific behavior within a scatterplot was strikingly consistent across studies, again demonstrating the replicability of results obtained with the SAM^2^ HBI.

#### Assessing the relation between habitualness and reward at the individual level

**Fig 14:**
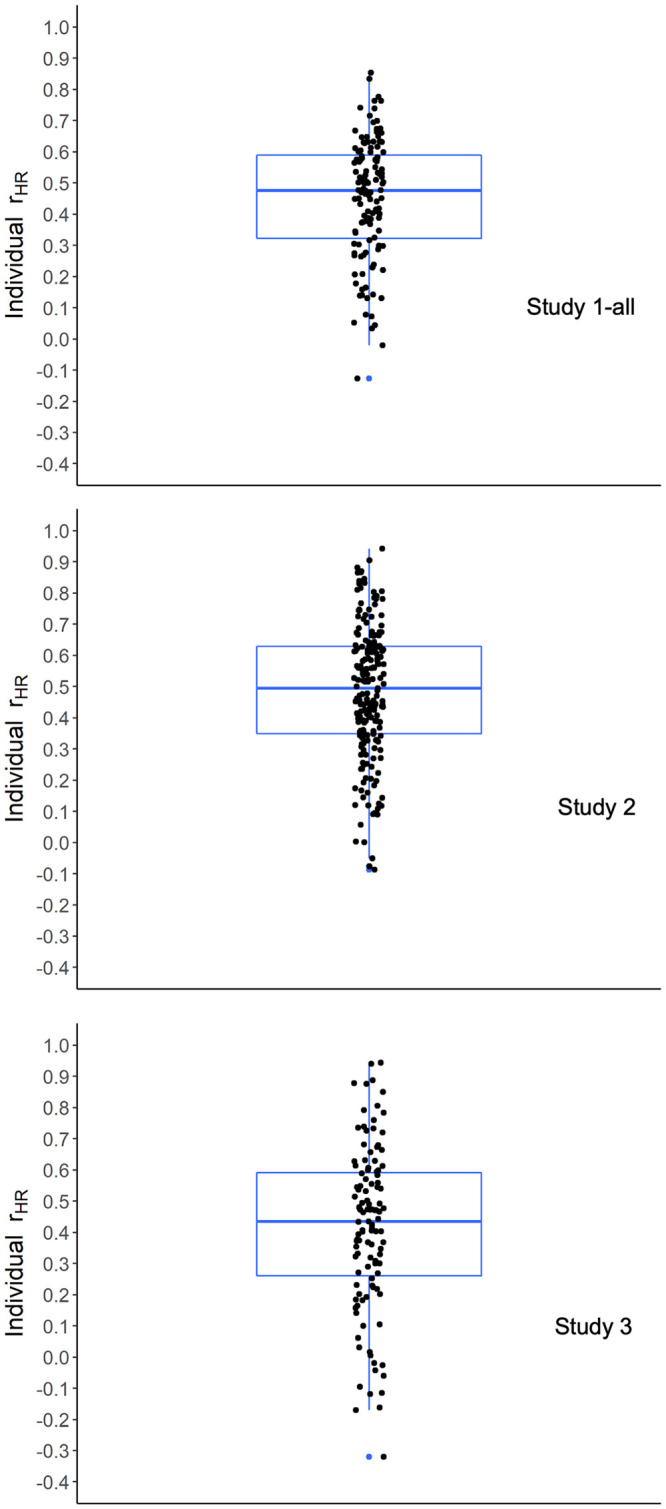
Values of the correlation between the composite measures of habitualness and reward, r_HR_, for individual participants in Studies 1-all, 2, and 3.

We next assessed the correlation between the composite measures of habitualness and reward for each individual across the 80 behaviors. Fig 14 plots the distribution of these correlations in each study, where a point represents the correlation for one individual. Similar to the analogous correlations at the group level, habitualness tended to be positively related to reward across individuals, with mean values of .45, .48, and .41 for Studies 1-all, 2, and 3, respectively (all different from 0, two-tailed: *t* = 26.27, SE = .02, *p* < .0001; *t* = 32.04, SE = .02, *p* < .0001; *t* = 16.86, SE = .02, *p* < .0001).

**Fig 15:**
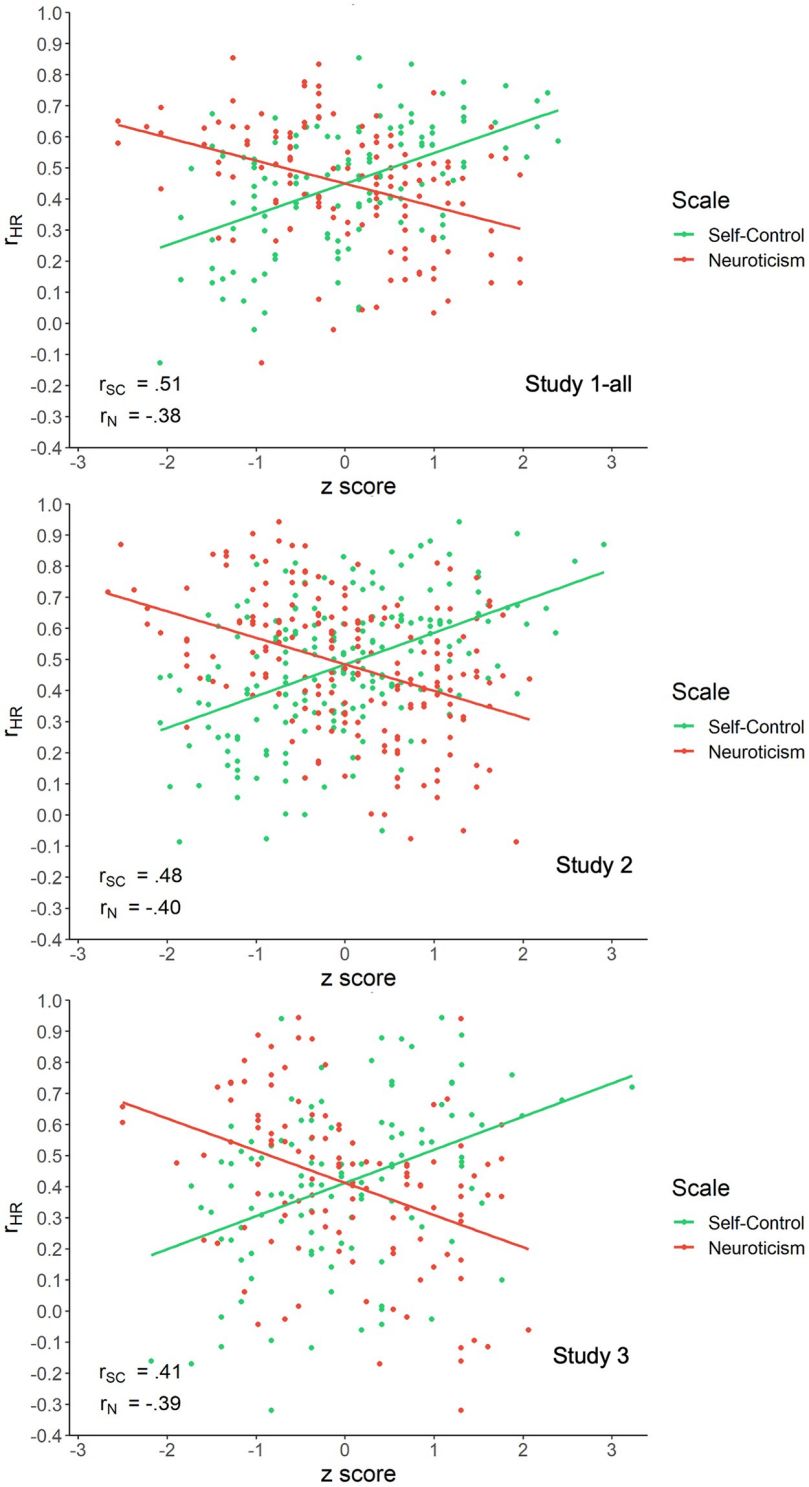
Individual correlations in Studies 1-all, 2, and3 between the composite measures of habitualness and reward, r_HR_, plotted as a function of individual measures for self-control and neuroticism. *Note*. The composite measures of habitualness and reward were both transformed to z scores before being plotted along the X axis.

Finally, we assessed whether individual correlations of habitualness and reward were related to the personality measures of self-control and neuroticism. As Fig 15 illustrates, a consistent pattern emerged across studies. As an individual’s self-control increased, the positive relation between reward and habitualness tended to become stronger, exhibiting positive correlations of .51, .48., and .41 for Studies 1-all, 2, and 3, respectively (every *p* < .0001, two-tailed). In contrast, as an individual’s neuroticism increased, the positive relation between reward and habitualness tended to become weaker, exhibiting negative correlations of -.38, -.40., and -.39 for Studies 1-all, 2, and 3, respectively (every *p* < .0001, two-tailed). These results suggest that self-control was associated with increased reward monitoring, whereas neuroticism was associated with decreased reward monitoring.

## Discussion

### Summary of results

Three studies demonstrated that the SAM^2^ HBI produces highly replicable findings. The same robust pattern of results that occurred in the larger samples of Studies 2 and 3 (*n* = 199 and *n* = 115, respectively) also occurred in samples as small as n = 31 (Studies 1a and 1b in Study 1-all). This pattern also replicated regardless of whether SAM^2^ judgments were collected in the laboratory (Studies 1a and 1b) or online (Studies 1c, 2, and 3), whether judgments were collected in a fixed order (Studies 1-all and 2) or in a random order (Study 3), whether additional measures were collected (Study 2). We next summarize the robust pattern of results that replicated across studies.

#### Trait-level measures

The SAM^2^ HBI produced a variety of trait-level measures that provided insight into behavior regularity at both the group and individual levels. Most basically, the SAM^2^ HBI produced a trait-level measure of behavior regularity, showing that, on average, participants performed the 80 behaviors in Table 1 about 50% of the time when possible (with some behaviors performed more regularly and others performed less). More specific trait-level measures for the regularity of positive behaviors and for the regularity of negative behaviors captured personality differences in self-control and neuroticism (Figs 3, 7 and 8).

Finally, a composite measure of habitualness established habitual behaviors at the group and individual levels (Figs 11 and 12), and a composite measure of reward established relations between habitualness and reward (Figs 13–15).

#### Individual differences

Participants exhibited considerable differences in the behaviors they performed regularly. When we assessed interrater reliability between participants in how regularly they performed the 80 behaviors, we only observed agreement around .30 (Table 2). For a composite measure of habitualness, participants similarly only exhibited agreement around .28. Behaviors that were habitual for some participants were not habitual for others. These results indicate that the SAM^2^ HBI captured large individual differences in habitual behavior. Consistent with the classic interactionist perspective [38–40, 45, 48], an individual’s behavior regularity varied considerably across situations, with the pattern of variability taking different forms for different individuals (i.e., the individual × behavior interaction in Fig 2).

Although such variability could simply reflect noise in judgments of regularity, individual regressions explained a median variance of approximately 75% in individual regularity judgments (Fig 6). Such a high level of explained variance suggests that individual regularity judgments were highly systematic—not noisy. If low interrater agreement had simply reflected noise, such high percentages of explained variance at the individual level would not have been observed.

#### Test reliability

When Cronbach’s alpha was used to assess test reliability, acceptable values around .80 and above were observed (Table 3). Interestingly, the 80 behaviors in the overall measure of behavior regularity exhibited relatively low coherence around .04, indicating that individual behaviors ordered participants quite differently. As we suggested, such low coherence most likely resulted from attempting to sample behaviors representatively. Broad coverage ensured that an overall measure of regularity reflected as many potentially relevant behaviors as possible.

Including a large number of 80 behaviors, however, compensated for low coherence, producing satisfactory values of alpha [23–26]. When coherence was computed separately for positive and negative behaviors, alpha increased as the behaviors within each group became more coherent (around .11).

#### Content and construct validity with respect to the Situated Action Cycle

When behavior regularity was regressed onto factors from the Situated Action Cycle associated with habitual behavior, multilevel mixed-effect regressions generally explained approximately 65% of the total variance at the group level. This explanatory success demonstrates content validity of the SAM^2^ HBI: Its measures across the Situated Action Cycle are sufficiently thorough and complete to explain the bulk of the variance in habitual behavior.

Two additional findings consistent with the habits literature further demonstrate construct validity for the SAM^2^ HBI (Fig 4). First, regularity was most highly associated with factors that reflect conditioning—consistency and automaticity. Second, immediate and long-term reward were also associated with regularity, albeit to a lesser extent. Because the SAM^2^ measure of behavior regularity captured this well-established pattern of results, it exhibited construct validity.

Regressions performed at the individual level were even more successful in establishing construct validity. As just noted, individual regressions explained more variance in regularity than did group-level regressions, with the median explained variance around 75% (Fig 6). Because regularity exhibited large individual differences, individual regressions explained more variance than group regressions, where individual differences attenuated explained variance. Explanatory success at the individual level again demonstrates content validity, with factors from the Situated Action Cycle explaining the bulk of the variance in behavior regularity.

Similar to the group-level regressions, regularity in the individual regressions tended to be best predicted by consistency and automaticity, with immediate and long-term reward making lesser contributions (Table 4). Nevertheless, substantial individual differences occurred, with different individuals exhibiting different patterns of prediction (Fig 5). As these patterns illustrate, the SAM^2^ HBI established individual models of behavior regularity, specifying factors from the Situated Action Cycle that best predicted regularity for each individual.

#### Validity with respect to self-control and neuroticism

Interactions of behavior valence with self-control and neuroticism further established the validity of the SAM^2^ HBI (Figs 7 and 8). In every study, increasing self-control was associated with increasing regularity of positive behaviors and decreasing regularity of negative behaviors (a self-control × valence interaction). Conversely, increasing neuroticism was associated with decreasing regularity of positive behaviors and increasing regularity of negative behaviors (a neuroticism × valence interaction). The fact that the SAM^2^ HBI captured these well-established interactions between personality and valence further demonstrates its construct validity.

Additional construct validity follows from SAM^2^’s ability to explain these interactions with factors from the Situated Action Cycle. As Figs 9 and 10 illustrate, automaticity, consistency, long-term reward, and immediate reward explained the variance in the interactions between valence, self-control, and neuroticism (across all three studies). These results suggest that self-control and neuroticism can be understood as emerging from individual patterns in these basic cognitive-affective processes [38–40, 45, 48].

#### The construct of habitualness

As Fig 5 illustrates, the automaticity of executing a behavior was not strongly associated with behavior regularity for all participants, nor was consistency. These results question whether automaticity and consistency are universal features of habitual behavior. As Fig 5 further illustrates, the predictive models for individual participants varied widely. Although clusters of individuals exhibited similar profiles of the factors that predict regularity, considerable differences existed between clusters. These large individual differences in predictive models further suggest caution in trying to develop a narrow definition of habitual behavior.

Still another problem is the distributions of the composite habitualness measure in Figs 11 and 12. On the one hand, it’s not clear where one can effectively set a threshold that distinguishes habits from non-habits. On the other, many behaviors fall in the middle of the distribution, where behaviors exhibit regularity, consistency, and automaticity a little over 50% of the time. Many of these behaviors could well be habitual, at least to some extent.

Together, these results suggest that a definition of habits based on necessary features such as automaticity and consistency may not be feasible or theoretically justifiable. Instead, the construct of habitualness may be better understood as a family resemblance structure, having prototypical features that are not definitional [136, 137]. From this perspective, habitual behaviors are statistically likely to be automatic, consistent, and rewarding, with none of these features being necessary. Because habitualness takes so many different forms, attempting to understand how its complex multidimensional structure manifests across individuals and behaviors may be more productive than attempting to establish a rigid definition. Similar conclusions have been reached about the construct of automaticity [138–140].

Finally, it may be useful to view this heterogeneous construct of habitualness as a continuous dimension. Rather than attempting to distinguish categories of habits from non-habits, it may be more useful to ask how habitual a behavior is, where habitualness tends to be continuously associated with increasing regularity, consistency, and automaticity.

#### The relation of reward to habitualness

A common view is that a behavior only becomes a habit if it is elicited ballistically, no longer modulated by reward. In their classic review of the non-human conditioning literature, Yin and Knowlton [118] document how organisms generally monitor reward and adjust behavior accordingly, with increases in reward increasing response rates to obtain reward (and vice versa). Reward only falls away at the very end of the conditioning process, following extreme levels of practice. Researchers often argue that humans never reach these levels, with reward always continuing to modulate behavior [78, 80, 88, 92]. Recent reviews document extensive evidence for the ever-present role of reward in habitual behavior, along with compelling theoretical arguments for why we should expect this [79, 83, 86].

Several of our results bear on this issue. Most basically, immediate and long-term reward were consistently related to behavior regularity at both the group and individual levels (Table 4, Figs 4 and 5). Additionally, composite measures of habitualness and reward were positively related at both the group and individual levels (Figs 13 and 14). As a behavior became more habitual, it became more rewarding, challenging the widespread assumption that reward becomes increasingly non-influential as behaviors become increasingly habitual. Instead, high levels of reward may induce high levels of regularity to maintain or increase the reward.

Finally, the relation between the composite measures of reward and habitualness behaved quite differently as self-control vs. neuroticism varied (Fig 15). As self-control increased, the positive relation between reward and habitualness increased as well. One speculative interpretation is that individuals high in self-control closely monitor sources of reward in their experience. When they find a reward of interest, they increasingly regularize the behavior needed to obtain it [68, 95, 106]. Conversely, the positive relation between reward and habitualness decreased as neuroticism increased (Fig 15). One speculative possibility is that the strong emotional reactions associated with neuroticism deplete the regulatory resources available for monitoring reward and adapting behavior as reward changes [141]. As a consequence, behavior becomes increasingly ballistic as poorly monitored behaviors become rigidly automated in response to situational cues.

### Limitations

#### Correlational results

As informative as these results are in assessing habitual behaviors at the individual and group levels, their correlational nature does not support causal interpretations. Indeed, one must be careful to avoid the temptation of drawing causal conclusions from the rich descriptive data available.

Responsible speculation about causality, however, can still be useful. On the one hand, correlational evidence can contribute to the accumulation of evidence related to causal claims, being consistent or inconsistent with them. On the other, correlational evidence is typically easier to gather than causal evidence and can thus serve as an effective form of discovery, to be followed later by careful experimental assessment when useful and possible.

#### Population sampled

Another limitation of the current findings is that they were obtained from UK students aged 18–30. It is important to explore whether the SAM^2^ HBI exhibits similar results in other populations. Although we are optimistic that these results will generalize robustly, verification is necessary. To the extent that different populations exhibit different habitual behaviors, the behaviors in Table 1 must be adapted. To the extent that relevant factors from the Situated Action Cycle vary, they may need to be adapted as well (Fig 1). We assume, however, that other implementations are likely to reproduce many features of the results reported here. Specifically, we anticipate that behavior regularity will exhibit similar relations to conditioning, reward, and personality, along with a comparable range of individual differences.

### Developing SAM^2^ assessment instruments

The SAM^2^ framework is sufficiently open-ended to support a wide variety of instruments for diverse purposes. We next explore a few of the possibilities.

#### Full-length SAM^2^ instruments

In contexts where thorough assessment of a construct is desired, a relatively comprehensive and representative set of situations may be optimal, along with a thorough sampling of relevant factors from the Situated Action Cycle. The SAM^2^ HBI explored here constitutes an example.

If one wanted to develop a version of the SAM^2^ HBI for another population, one could sample behaviors and factors from the Situated Action Cycle differently. To assess habitual behavior in children across levels of socioeconomic status, for example, one could sample behaviors related to education, play, and family, along with factors from the Situated Action Cycle related to resources, supervision, and choice. Alternatively, to assess habitual behavior in socially anxious adults, one could focus on social and achievement behaviors, along with factors from the Situated Action Cycle related to threat, social support, and acceptance.

More generally, SAM^2^ instruments can be developed for many other constructs besides habitual behaviors. In our work, we have explored instruments for stress, trichotillomania, social connectedness, eating, drinking, emotion regulation, mindfulness, wellbeing, and sustainable behavior. In each case, we first establish relevant situations where the target construct occurs and develop a set of systematic cues for activating them (often informed by empirical norming). We then establish a measure of the construct. Finally, we examine the relevant scientific literature to identify factors from the Situated Action Cycle known to influence the construct in the situations of interest. We then build a SAM^2^ instrument around this content. Typically, we find that such instruments take 30 to 60 minutes for participants to complete, depending on the domain.

#### Brief SAM^2^ instruments

We appreciate the need for briefer instruments in some contexts. One brief instrument that may often be sufficient is to only obtain judgements of the target construct across situations and to *not* collect judgments for influential factors from the Situated Action Cycle. To the extent that a larger initial study has validated judgments of the construct with respect to these factors, judgments of the target construct alone may be sufficient for many purposes.

Consider the SAM^2^ HBI developed here. A brief version would only include regularity judgments for the 80 situations (taking 5–10 minutes). We now know that regularity judgments exhibit construct validity because we validated them here in relation to factors from the Situated Action Cycle and to personality measures. We also know that constructing separate overall trait-level measures for the regularity of positive vs. negative behaviors can be informative, making them potentially applicable in a variety of clinical and applied settings.

A second way to create a brief SAM^2^ instrument is to compress either or both dimensions of situatedness: (a) situations, (b) factors from the Situated Action Cycle. To compress situations, cluster analyses could establish clusters of related behaviors, with the most central behavior sampled for each cluster. Another possibility would be to sample one positive behavior and one negative behavior from each of the domains in Table 1, reducing the number of behaviors assessed from 80 to 20. To compress factors from the Situated Action Cycle, a set of existing factors, such as those in Fig 1, could be submitted to exploratory factor analysis. Again, groups of closely related measures from the Situated Action Cycle could be identified, with only one from each group included in a brief instrument.

In this manner, brief compressed versions of full-length instruments can be developed. It is an empirical question whether a brief version is as informative as a complete version. In clinical settings, another interesting question is whether using a brief version leads to comparable insight and behavior change in clients as using a complete version.

#### Targeted SAM^2^ instruments

A different approach to creating brief instruments is to increasingly focus the target construct and then only sample situations and factors from the Situated Action Cycle relevant to this narrower focus. In the domain of habitual behavior, for example, one could focus on health behaviors, social behaviors, or sustainable behaviors. For each focus, a smaller set of behaviors becomes relevant, and perhaps also fewer factors from the Situated Action Cycle.

Another approach is to create a brief SAM^2^ instrument adapted to a specific individual. By only including situations and factors relevant for the individual, a brief instrument for individual use can be created. In the domain of habitual behaviors, for example, a unique instrument for each individual could be developed that tracks their most positive and negative behaviors over time. Such instruments might also be useful in experience sampling applications.

*Clinical use*. SAM^2^ instruments could be designed to support a variety of clinical functions. Most basically, such instruments could map out a client’s habitual tendencies and identify factors from the Situated Action Cycle associated with them. For example, such instruments could establish the regularity of performing positive versus negative behaviors (or other target behaviors of interest). A predictive model could then be developed of factors from the Situated Action Cycle associated with these behaviors, with this model then being used to guide behavior change interventions. Finally, longitudinal assessment could track the effectiveness of these interventions over time.

## Conclusion

From the theoretical perspectives of grounded, situated, and embodied cognition, we have developed a new approach for assessing individual differences—the *Situated Assessment Method* (SAM^2^). Rather than asking individuals to abstract across situations when evaluating a target construct, SAM^2^ situates the construct in two ways: First, SAM^2^ assesses the construct in situations where it occurs (using features of these situations to activate them). Second, SAM^2^ assesses factors from the Situated Action Cycle that potentially influence the construct in these situations. The result is a rich descriptive profile for each individual that embeds the construct in their unique situational experience of it.

In the implementation for habitual behaviors developed here, we saw that this approach produces useful measures of behavior regularity that are reliable, valid, and potentially useful for a variety of functions. For each individual, a detailed profile of habitual behavior emerges. The general SAM^2^ framework can be implemented in diverse ways to achieve a wide variety of assessment goals.

## Supporting information

S1 File Supplemental materials for the methods, analyses, and results of all studies. (PDF)
