# Supplementary material for: The Situated Assessment Method (SAM2): Establishing individual differences in habitual behavior
Source: PLoS One. 2023 Jun 22;18(6):e0286954. doi: 10.1371/journal.pone.0286954 (PMC10287018; doi:10.1371/journal.pone.0286954)
Supplement: S1 File — (PDF) [file pone.0286954.s001.pdf]

Supplemental Material

The Situated Assessment Method (SAM^2): Establishing individual differences in habitual behavior

Léo Dutriaux[1] Naomi E. Clark[2] Esther K. Papies[3] Christoph Scheepers[3] Lawrence W. Barsalou[3]

[1] Laboratoire sur les Interactions Cognition, Action, Émotion (LICAÉ), Université Paris Nanterre, 200 avenue de La République, 92001 Nanterre Cedex, France

[2] School of Health and Life Sciences, Glasgow Caledonian University

[3] School of Psychology and Neuroscience, University of Glasgow

1. Screens for collecting judgments across studies together with the associated rationale and supporting data ... 3
Figure SM-1. Screens for collecting judgments in Study 1-all ... 4
Figure SM-2. Screens for collecting judgments in Study 2 ... 5
Figure SM-3. Screens for collecting judgments in Study 3 ... 6

2. Group correlation matrices ... 7
Table SM-1. Correlation matrices in Studies 1-all, 2, and 3 ... 8

3. Judgment means for the 80 behaviors in Study 2 ... 9
Table SM-2. Average SAM^2 judgements for each behavior in Study 2 ... 10

4. Regression analysis pipeline ... 12

5. Assessing the validity of the a priori valence assignments ... 14
Figure SM-4. Rated valence judgments for the 80 behaviors as a function of a priori valence. 15
Table SM-3. Regression results for predicting a priori and rated valence in Study 2 ... 16

6. Clustered heatmaps for individual × behavior interactions ... 17
Figure SM-5. Individual × behavior interaction in Study 1-all ... 18
Figure SM-6. Individual × behavior interaction in Study 3 ... 19

7. Regression results for the group-level analyses of Studies 1-all, 2, and 3 ... 20
Table SM-4. Regression results for predicting regularity in Study 1-all ... 21
Table SM-5. Regression results for predicting regularity in Study 2 ... 23
Table SM-6. Regression results for predicting regularity in Study 3 ... 25

8. Clustered heatmaps of individual predictive correlations ... 27
Figure SM-7. Clustered heatmap of individual predictive correlations in Study 1-all ... 28
Figure SM-8. Clustered heatmap of individual predictive correlations in Study 3 ... 29

9. Regression analyses that assessed self-control and neuroticism ... 30
Table SM-7. Regression results for assessing interactions of valence and personality ... 31

10. Stepwise regressions to establish processes from the Situated Action Cycle that underlie self-control and neuroticism ... 32
Figure SM-9. Valence × self-control interactions for full models in Studies 1-all, 2, and 3 ... 33
Figure SM-10. Valence × neuroticism interactions for full models in Studies 1-all, 2, and 3 ... 34
Table SM-8. Stepwise regressions to explain the valence × self-control interactions ... 35
Table SM-9. Stepwise regressions to explain the valence × neuroticism interactions ... 36

11. Valence × personality interactions using *rated* valence in Study 2 37
Figure SM-11. Valence × personality interactions for a priori and rated valence in Study 2 38

12. Analysis of social approval as a predictor of behavior regularity 39
Figure SM-12. Regression coefficients for predicting regularity and motivation in Study 2 40
Table SM-10. Regression results for regularity in Study 2, including social approval............. 41

13. Predicting behavior motivation 43
Table SM-11. Regression results for predicting behavior motivation in Study 2 44

14. Assessing explicit awareness of factors that predict behavior regularity 47
Figure SM-13. Screen for collecting meta-cognitive prediction estimates in Study 3 48
Figure SM-14. Correlations between estimated and actual predictive correlations 49

1. Screens for collecting judgments across studies together with the associated rationale and supporting data

Examples of screens used to collect judgments in Studies 1-all, 2, and 3

Figure SM-1 illustrates the 3 screens used to collect judgments in Study 1-all. Figure SM-2 illustrates the 4 screens used to collect judgments in Study 2. Figure SM-3 illustrates the 6 screens used to collect judgments in Study 3. The rationale for using these various screen formats is presented below, along with results that bear on their use.

Rationale for using blocked judgments in Studies 1-all and 2

In Study 1-all, we implemented the six judgments as two pairs of judgments in a fixed sequence of three blocks for three reasons: First, collecting two judgments in each of three blocks significantly decreased the total time required to perform the study, relative to the six blocks that would have been required for performing one judgment per block.

Second, we believed that performing related pairs of judgments would help participants perceive the differences between them, thereby producing more accurate judgments. Specifically, we assumed that judging regularity and consistency together would help participants discriminate the difference between how regularly a behavior occurs versus how consistently it occurs in the same situation. Similarly, we assumed that judging immediate and long-term reward together would help participants discriminate these two kinds of reward.

Third, we assumed that judging conflict and automaticity together would help participants perceive the difference between effortfully deciding to perform a behavior versus performing it with little thought. Instructions for each pair of judgments helped participants discriminate the two judgments.

Finally, we believed that judging the six scales in a fixed order made the task more intuitive for participants. Specifically, it seemed most natural to begin with generally assessing the regularly of performing a behavior and the consistency of the situations where it is performed. It then seemed intuitive to have participants focus on contrasting the immediate reward and long-term reward of the behavior. Finally, it seemed natural to have participants conclude with assessing the conflict associated with performing the behavior (perhaps associated with the contrast between immediate and long-term reward), followed by assessing how automatically they perform it.

Results that bear on use of blocked judgments

As the main article and the SM document extensively, the same general pattern of results occurred for Studies 1-all and 2 (where blocked judgments were collected) and for Study 3 (where individual randomized judgments were collected). The effects of the personality variables and the prediction of behavior regularity remained remarkably constant across all these studies at both the group and individual levels. Additionally, blocking did not alter the general patterns observed for the intraclass correlations, correlation matrices, and individual difference data.

As Table SM-11 illustrates for pairs of measures collected together in Studies 1-all and 2, blocked data collection had little effect compared to individual randomized data collection in Study 3. First, the correlation between regularity and consistency was actually slightly higher when they were collected separately in Study 3 (.71) than when they were collected together in Studies 1-all and 2 (.68 and .66, respectively). Second, the correlations between immediate and long-term reward in Studies 1-all and 2 (.33 and .46, respectively) were comparable to the correlation in Study 3 (.27). Third, the correlations between conflict and automaticity in Studies 1-all and 2 (-.04 and .03, respectively) were comparable to the correlation in Study 3 (.01).

Together these results indicate that blocking judgments together had little impact on the results obtained.

Figure SM-1. Examples of the three screens used to collect judgments in Study 1-all.

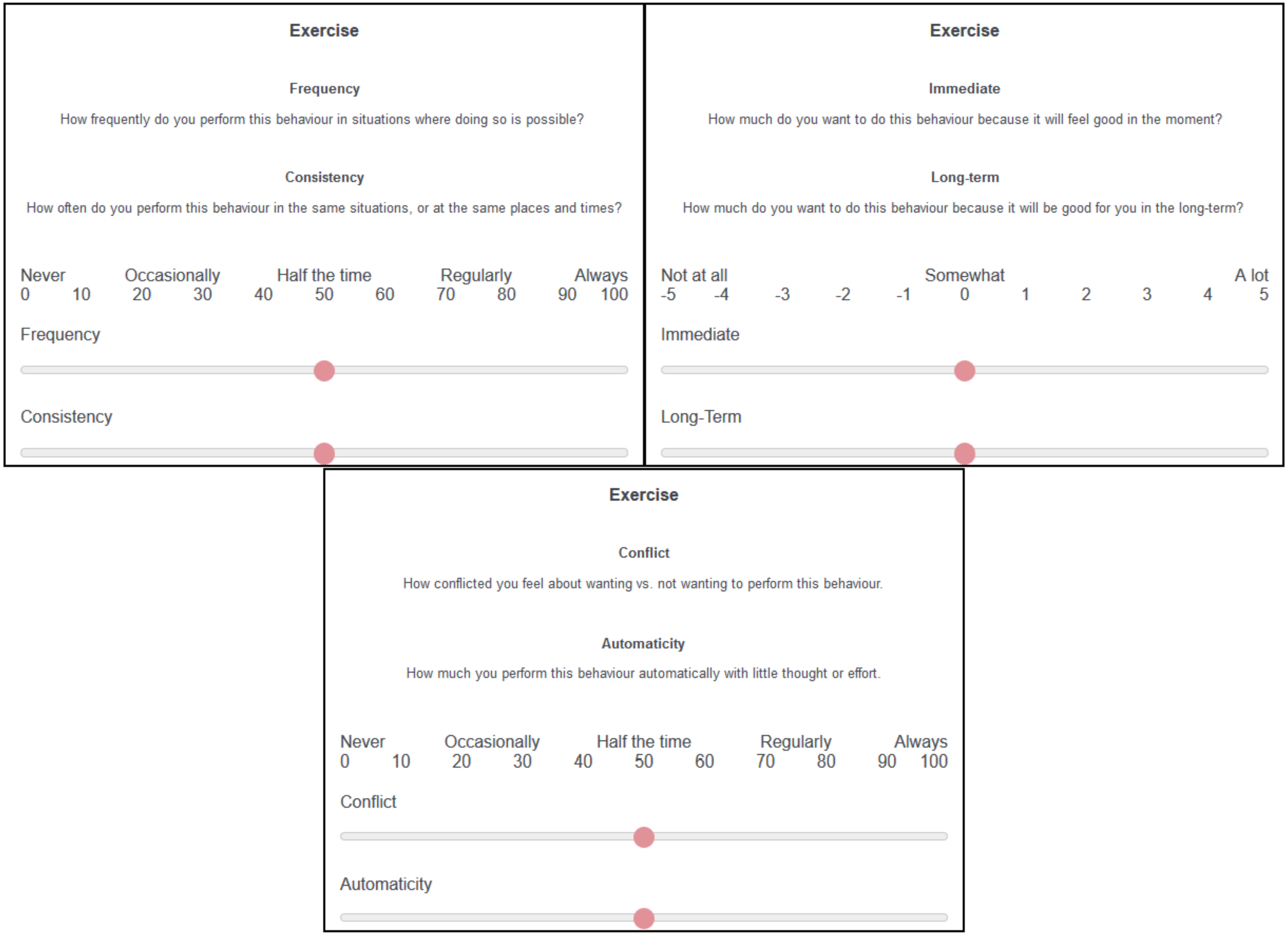

Figure SM-2. Examples of the four screens used to collect judgments in Study 2.

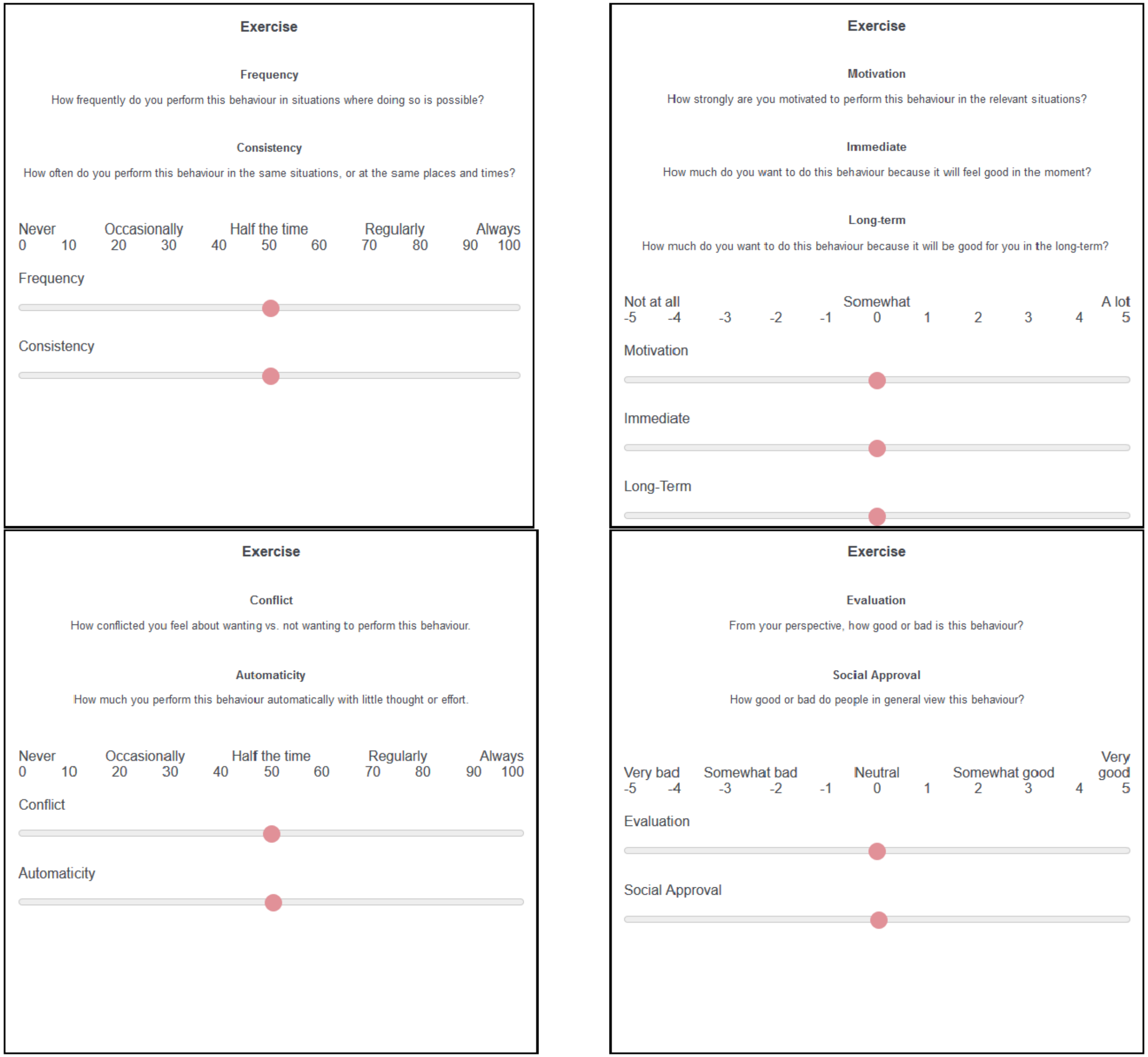

Figure SM-3. Examples of the six screens used to collect judgments in Study 3.

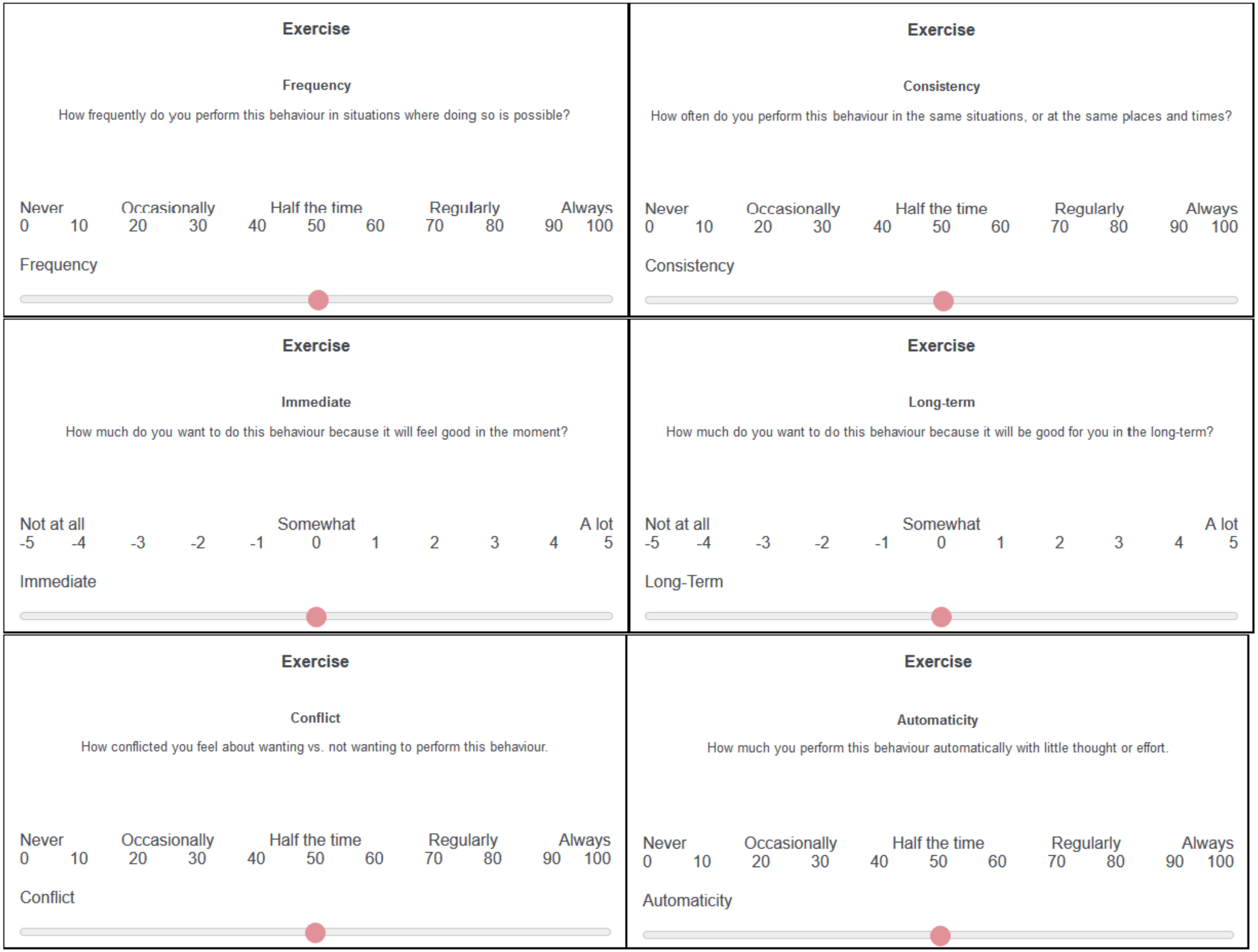

2. Group correlation matrices

Table SM-1 presents the correlation matrices for all three studies. As can be seen, the same general pattern of correlations emerged across studies. See the table heading and the main article for further details.

Table SM-1. Pearson correlations between measures in Studies 1-all/2/3 (across participants and behaviors within participants).

	Consistency	Immediate Reward	Long-term Reward	Conflict	Automaticity	Self-control	Neuroticism	Social Approval	Motivation	Rated Valence
Regularity	.68/.66/.71	.47/.50/.39	.35/.39/.30	-.05/.03/.00	.66/.61/.66	-.03/-.04/-.01	.01/.03/-.01	/.24/	/.66/	/.41/
Consistency		.33/.40/.38	.24/.29/.27	-.04/.02/.02	.50/.45/.64	-.03/-.07/.01	.01/.03/.01	/.19/	/.48/	/.28/
Immediate Reward			.33/.46/.27	.01/.06/.07	.39/.42/.38	-.04/-.02/.02	.01/.03/-.03	/.27/	/.70/	/.39/
Long-Term Reward				-.20/-.06/-.07	.20/.26/.14	.00/-.01/.04	.00/-.01/-.05	/.61/	/.50/	/.68/
Conflict					-.04/.03/.01	-.11/-.09/-.04	.08/.12/.05	/-.06/	/.05/	/-.06/
Automaticity						-.06/-.05/-.01	.03/.06/-.01	/.14/	/.53/	/.30/
Self-control							-.34/-.48/-.23	/.01/	/-.04/	/-.02/
Neuroticism								/-.01/	/.04/	/.01/
Social Approval									/.32/	/.76/
Motivation										/.48/

Note. Correlations are significant at $p < .05$, two-tailed when larger than |.18|, |.14|, and |.19| in Studies 1-all, 2, and 3, respectively (reflecting sample sizes of 128, 199, and 115, respectively). Correlations are significant at $p < .01$ (two-tailed) when larger than |.23|, |.19|, and |.24| in Studies 1-all, 2, and 3, respectively. Correlations are significant at $p < .0001$, two-tailed when larger than |.29|, |.24|, and |.31| in Studies 1-all, 2, and 3, respectively.

3. Judgment means for the 80 behaviors from Study 2

For researchers interested in using norms established here for the 80 behaviors, Table SM-2 presents the mean standardized rating across participants for each behavior on each SAM^2 judgment in Study 2. Study 2 was used because it contained the greatest number of measures (similar norms can be computed for Studies 1-all and 3 using the data files and scripts on the OSF site for this article). The means and standard deviations used to compute the standardized values are shown at the bottom, such that mean values in the original scales can be recovered.

Table SM-2. For each of the 80 behaviors in Study 2, the standarized average rating across participants is shown. The means and standard deviations used to compute the standardized values are shown at the bottom, such that mean values in the original scales can be recovered. A sortable spreadsheet for this table can be found at: https://osf.io/s34bj/

			Average rating								
**Domain**	**Valence**	**Behavior**	**Regularity**	**Consistency**	**Motivation**	**Immediate**	**Long-Term**	**Conflict**	**Automaticity**	**Rated Valence**	**Social Approval**
**Food and drink**	-	Drink alcohol.	-0.97	-0.62	-0.50	0.17	-1.16	-0.22	-0.95	-0.60	-0.11
	-	Eat dessert.	-0.51	-0.40	0.38	0.83	-0.86	0.82	-0.47	-0.21	-0.16
	-	Eat fast foods.	-0.72	-0.50	0.14	0.73	-1.19	1.63	-0.80	-0.94	-0.95
	-	Drink soft drinks.	-0.04	0.25	0.09	0.53	-0.83	0.47	-0.06	-0.63	-0.53
	+	Eat fruit.	0.58	0.32	0.92	1.01	1.25	-1.25	0.45	1.12	1.16
	+	Eat healthy snacks.	0.08	-0.07	0.52	0.48	1.19	-0.03	0.06	0.95	1.06
	+	Eat vegetables.	1.12	1.20	1.04	0.86	1.34	-0.67	0.89	1.22	1.22
	+	Check food labels before making purchases.	0.32	0.39	0.50	0.06	0.87	-1.18	0.42	0.69	0.46
**Exercise**	-	Be sedentary for long periods of time.	0.09	0.33	-0.41	-0.32	-1.03	0.89	0.66	-1.09	-1.11
	-	Avoid long walks.	-1.28	-1.10	-1.16	-0.91	-0.94	-0.20	-0.90	-1.15	-0.91
	-	Reward myself with food and/or drink after exercise.	-0.44	-0.52	0.14	0.36	-0.76	0.51	-0.22	-0.39	-0.43
	-	Use the lift instead of taking the stairs.	-0.59	-0.53	-0.21	0.08	-0.57	0.14	-0.07	-0.56	-0.36
	+	Exercise.	-0.27	-0.23	0.01	-0.02	1.10	1.06	-0.71	1.03	1.19
	+	Walk or bike when possible .	0.35	0.22	0.54	0.39	1.06	0.10	0.23	0.92	0.95
	+	Participate in sports activities and clubs.	-1.48	-1.34	-0.88	-0.54	0.51	-0.29	-1.71	0.53	0.95
	+	Take standing and walking breaks when sitting for long periods of time.	-0.35	-0.29	0.03	0.36	0.82	-0.37	-0.51	0.69	0.60
**Affective**	-	Use substances to relax.	-1.83	-1.91	-1.84	-1.53	-1.34	-0.94	-1.98	-1.07	-1.15
	-	Worry.	0.94	0.63	-0.18	-1.71	-1.15	2.06	1.48	-1.07	-1.11
	-	Criticise myself.	0.93	0.53	0.19	-1.49	-0.73	1.20	1.08	-0.75	-0.83
	-	Ignore my own needs	-0.11	-0.16	-0.81	-1.54	-1.03	1.44	0.26	-1.21	-1.05
	+	Take time to relax.	0.45	0.50	1.25	1.66	0.77	0.61	0.38	0.85	0.67
	+	Do at least one thing a day that I enjoy and look forward to.	0.38	-0.04	0.95	1.29	0.82	0.05	0.01	0.99	0.94
	+	Express my emotions constructively.	-0.19	-0.42	0.28	0.20	0.78	0.83	-0.74	0.71	0.71
	+	View challenges with a positive attitude.	0.41	0.11	0.57	0.74	1.06	0.40	-0.23	0.89	0.96
**Social**	-	Use bad language in public.	-0.41	-0.94	-1.08	-0.90	-1.08	0.12	-0.03	-1.29	-1.53
	-	Interrupt others.	-1.42	-1.59	-1.71	-1.77	-1.26	0.15	-1.39	-1.59	-1.59
	-	Pay little attention to others when they are talking.	-1.31	-1.22	-1.43	-1.44	-1.14	-0.27	-0.75	-1.46	-1.42
	-	Make myself the centre of conversation.	-1.44	-1.35	-1.76	-1.56	-0.95	-0.50	-1.76	-1.11	-0.94
	+	Maintain contact with family.	0.94	0.99	0.89	0.87	1.07	-0.12	0.50	0.99	1.10
	+	Maintain contact with friends.	0.46	0.10	0.91	1.08	1.08	0.15	0.17	0.98	1.19
	+	Hold doors open for others.	1.48	1.02	1.41	1.54	0.57	-1.53	1.96	1.22	1.11
	+	Say "please" and "thank you".	2.00	2.09	1.90	1.87	1.19	-2.06	2.26	1.40	1.27
**Technology**	-	Spend a large amount of time on social media.	0.76	0.91	0.46	0.53	-0.72	0.58	1.36	-0.78	-0.55
	-	Use my phone as a social crutch (e.g. use my phone when I am alone in social situ	1.03	1.09	0.95	1.08	-0.40	0.21	1.22	-0.45	-0.67
	-	Check my phone multiple times a day.	1.99	1.84	1.33	1.21	-0.25	-0.22	2.02	-0.33	-0.39
	-	Use my phone whilst on the toilet	0.66	0.86	0.44	0.57	-0.33	-1.42	0.91	-0.28	-0.57
	+	Make back-up copies of important documents and files.	-0.13	-0.08	0.31	-0.35	1.14	-0.92	-0.52	1.05	0.88
	+	Charge my devices.	1.65	2.00	1.55	1.23	1.03	-1.71	1.97	0.79	0.61
	+	Limit the amount of time each day I spend using technology.	-1.93	-1.77	-1.34	-1.25	0.26	0.35	-1.41	0.33	0.33
	+	Restrict my use of technology before sleep.	-2.01	-1.73	-1.43	-1.27	0.34	0.74	-1.57	0.43	0.53
**Work and study**	-	Procrastinate.	0.79	0.62	0.48	0.55	-1.18	2.25	0.87	-1.09	-1.14
	-	Work whilst watching TV or listening to music.	0.53	0.49	0.49	0.67	-0.32	-0.08	0.62	-0.10	-0.48
	-	Skip lectures.	-1.87	-1.78	-1.43	-0.98	-1.50	0.84	-2.03	-1.53	-1.43
	-	Multi-task during work.	0.67	0.65	0.54	0.31	0.23	0.07	0.65	0.22	0.19
	+	Study for my course(s).	0.98	0.93	0.80	0.14	1.28	0.81	0.00	1.30	1.20
	+	Take study breaks	0.28	0.01	0.81	0.90	0.36	0.80	-0.06	0.69	0.65

	+	Set goals before engaging in a task.	0.14	-0.13	0.43	0.39	1.01	-0.39	-0.25	0.76	0.89
	+	Pack what I need the night before.	0.35	0.46	0.38	0.07	0.95	0.18	-0.11	0.74	0.56
**Personal hygiene**	-	Pick my nose.	-0.98	-1.23	-1.10	-0.65	-0.93	-0.70	-0.11	-1.22	-1.79
	-	Pick my spots and/ or scabs.	0.02	-0.02	-0.57	-0.20	-1.31	0.59	0.19	-1.22	-1.53
	-	Chew on pencils and/ or pens.	-1.75	-1.79	-2.13	-1.99	-1.39	-1.64	-1.36	-1.24	-1.29
	-	Bite my nails.	-1.10	-1.47	-1.56	-1.50	-1.41	-0.62	-0.72	-1.40	-1.48
	+	Shower every day.	1.03	1.47	1.12	1.47	1.08	-1.26	1.21	1.10	1.17
	+	Cover my mouth when sneezing, coughing or yawning.	1.66	1.55	1.63	1.29	0.88	-1.74	1.85	1.26	1.18
	+	Brush my teeth twice a day.	0.66	1.05	0.79	0.80	1.27	-0.29	0.57	1.23	1.30
	+	Go to sleep and wake up at the same times.	-0.37	-0.18	-0.05	-0.19	0.80	0.61	-0.49	0.56	0.64
**Household**	-	Allow messes to build up in my work area.	-0.43	-0.32	-1.32	-1.29	-1.16	0.35	0.10	-1.19	-1.28
	-	Ignore stains and spills.	-1.63	-1.26	-1.45	-1.38	-1.13	0.10	-1.28	-1.36	-1.48
	-	Leave dishes to wash later	0.24	0.44	-0.19	0.06	-1.07	1.24	0.21	-0.97	-0.97
	-	Leave clothes lying around.	0.04	0.43	-0.61	-0.42	-1.22	0.08	0.40	-1.10	-1.24
	+	Wash my clothes.	1.19	1.40	0.97	0.69	1.18	-0.50	0.72	1.22	1.17
	+	Put things back after I have finished using them.	0.74	0.63	0.55	0.09	1.02	-0.13	0.62	0.87	0.79
	+	Empty the bins.	0.50	0.66	-0.05	0.05	0.94	0.35	0.23	0.96	0.96
	+	Clean my residence	0.78	0.90	0.46	0.70	1.17	0.70	0.16	1.10	1.08
**Finance**	-	Dip into funds I have set aside.	-0.58	-0.89	-0.74	-0.22	-1.18	2.40	-1.29	-1.31	-0.98
	-	Spend to make myself feel better.	-0.39	-0.70	0.11	0.70	-0.86	1.74	-0.47	-0.53	-0.20
	-	Buy brand name products.	0.06	-0.04	0.02	0.05	-0.29	0.05	-0.14	-0.13	0.27
	-	Make impulsive purchases	-0.45	-0.96	-0.12	0.54	-1.10	2.11	-0.40	-0.89	-0.67
	+	Budget.	0.50	0.35	0.69	0.05	1.24	0.20	-0.25	0.91	0.80
	+	Buy from charity and/ or second-hand shops.	-1.20	-1.23	-0.48	-0.43	0.18	-1.22	-1.35	0.62	0.39
	+	Use shopping lists	-0.20	0.21	0.15	0.34	0.55	-1.14	-0.33	0.58	0.48
	+	Shop for groceries.	1.01	1.10	0.77	0.63	0.93	-0.56	0.48	0.81	0.74
**Environment**	-	Litter.	-2.58	-2.58	-2.90	-2.79	-1.58	-1.09	-2.43	-1.97	-1.81
	-	Buy new condition items	0.52	0.17	0.52	0.69	0.36	0.22	0.19	0.18	0.42
	-	Leave plug sockets switched on	-0.21	0.13	-1.18	-1.50	-0.99	-0.90	0.04	-1.19	-1.06
	-	Throw away food.	-0.96	-0.57	-1.64	-1.78	-1.16	1.20	-1.06	-1.46	-1.39
	+	Turn off lights when leaving a room.	0.76	0.81	0.77	0.17	0.99	-1.69	1.25	0.91	0.83
	+	Recycle.	1.05	1.11	1.04	0.47	1.21	-1.13	0.97	1.23	1.21
	+	Reuse carrier bags.	1.02	0.98	1.02	0.61	1.02	-1.26	0.92	1.07	1.00
	+	Use reusable cups.	0.02	0.00	0.05	-0.24	0.55	-1.19	0.35	0.75	0.76
		**Grand Mean**	54.81	59.83	0.86	1.09	0.52	40.77	55.56	0.88	0.99
		**SD**	17.00	12.39	1.45	1.36	2.53	9.28	14.05	2.34	2.43

4. Regression analysis pipeline

The regression analysis pipeline described here was used in all regression analyses to follow. All later tables of regression results were produced using this pipeline.

The primary goals of our analysis pipeline were to: (1) identify effects, (2) establish their effect sizes, and (3) assess their generalizability across participants and behaviors. The dependent variable (behaviour regularity) and its predictors (e.g., consistency immediate reward, long-term reward, conflict, automaticity) were all standardized so that we could specify each predictor's effect in standard deviation units. Thus, each estimated regression coefficient provides a measure of effect size, indicating the standard-deviation-unit change in the dependent variable associated with each standard-deviation-unit change in the predictor. The sign of these standardized coefficients further indicates the direction of the relationship. If, for example, a standardized coefficient for the relation between automaticity and behaviour regularity happened to be .60, this meant that behaviour regularity increased positively by .60 of a standard deviation for each standard deviation increase in automaticity. The larger the absolute value of a coefficient, the larger its effect size.

For each regression analysis, we implemented a sequence of three multilevel mixed-effect models (using the lme4 package in R; Bates, Mächler, Bolker, & Walker, 2015). We will refer these models as *Model 1*, *Model 2*, and *Model 3*. These models were *multilevel* because they predicted a dependent variable such as behaviour regularity using both behavior-level predictors (consistency, immediate reward, long-term reward, conflict, automaticity) and individual-level predictors (self-control, neuroticism). These models were *mixed effect* because they simultaneously assessed both fixed effects (predictors at the individual and behavior levels) and random effects (random intercepts and slopes capturing variability of effects at the individual and behavior levels). Assessing random effects is pivotal for generalizing results beyond a current sample of participants and behaviors (Barr, Levy, Scheepers, & Tily, 2013). Multilevel mixed-effect modelling offers a powerful approach for establishing generalizability across participants and behaviors simultaneously.

In the first stage of our analysis pipeline, Model 1 identified predictors likely to have meaningful effects on the dependent variable (both main effects and interactions). Model 1 included all predictors of interest at the behavior and individual levels, all interactions up through three-way, and random intercepts for behaviors and participants. This relatively liberal model served to identify potentially important predictors that were subsequently examined more closely and conservatively in Models 2 and 3. For a predictor to pass this initial screening, the t for its estimated regression coefficient had to be greater than $|1.96|$ (associated with a p-value $\leq .05$). We assumed that any effect that failed this initial screening would be unlikely to have a meaningful impact on the dependent variable.

For each potentially important predictor identified in Model 1, we then assessed it more conservatively in a unique Model 2 that tested it *maximally* (Barr et al., 2013). Specifically, maximal testing established whether a predictor's effect in Model 1 generalized beyond participant-level and behavior-level variability for the effect in the underlying population of possible observations. Imagine, for example, that the .60 estimated regression coefficient for automaticity survived initial screening in Model 1. If large individual differences in participants and behaviors are present, then the observed effect may not generalize to the broader populations of participants and behaviors. To test an observed effect in Model 1 maximally, Model 2 included one empirically-determined random slope for each participant that modeled the effect for that participant. Additionally, Model 2 included one empirically-determined random slope for each behavior that modeled the effect for that behavior. Of interest was whether the t for the fixed effect in Model 2 remained greater than $|1.96|$ once the variances of the random effects for participants and behaviors were accounted for simultaneously. If the fixed effect passed this maximal testing, we concluded that it generalizes beyond the current samples of participants and behaviors. If the effect failed maximal testing, we assumed that it does not generalize and can be explained in terms of individual-level and behavior-level variability.

Unfortunately, including appropriate random slopes simultaneously in Model 2 for each and every predictor that survives initial screening in Model 1 is typically not possible, as the sheer complexity of the model disrupts optimization and convergence. To circumvent this problem, Barr et al. (2013, p. 276) suggested maximally testing each effect of interest one at a time (i.e., including appropriate random slopes for participants and behaviors associated with the fixed effect of interest, while not including random slopes for any remaining fixed effects). Thus, when maximally testing the effect of (say) automaticity, a unique Model 2 was constructed by adding random slopes for automaticity to Model 1 but not adding random slopes for any other fixed effect. In this manner, a unique Model 2 was constructed for each fixed effect that passed Model 1 screening. Importantly, whenever a higher-order *interaction* passed Model 1 screening, random slopes were also included for all lower-order interactions and main effect terms nested within it (see Barr et al., 2013).

If a predictor passed maximal testing in Model 2, it was evaluated one more time in a unique Model 3 that established how much unique variance in Model 2 was associated with it. In each Model 3, we dropped the main effect or interaction being tested from its Model 2, along with any interactions containing it and any associated random slopes, while keeping everything else the same as in Model 2. We then subtracted the total variance for the predictor's Model 3 from the total variance for its Model 2. The difference in R^2 (ΔR^2 expressed as a percentage) established how much unique variance the predictor captured when included *as a fixed effect together with associated random effects* in Model 2.

Using this analysis pipeline, we established predictors associated with effects that generalize across participants and behaviors (i.e., predictors that survived maximal testing in Model 2). For each predictor that generalized, we obtained two measures of its effect size: (1) its standardized regression coefficient in Model 2, and (2) its ΔR^2 derived from Model 3.

5. Assessing the validity of the a priori valence assignments

Participants in Study 2 rated the valence of each behavior in Table 1. For each behavior participants were asked to judge, “From your perspective, how good or bad is the behaviour?”, using a scale from -5 to 5, with the labels: Very bad, Somewhat bad, Neutral, Somewhat good, Very good. The intraclass correlation for inter-rater agreement was .55 (ICC2).

We predicted that rated evaluations of behavior valence in Study 2 would confirm our a priori assignments of positive versus negative behavior valence in Table 1. The point-biserial correlation between these two judgments, .68, supports this prediction. Figure SM-4 plots the average rated valence of the a priori positive and negative behaviors in the original scale units (-5 to +5). As can be seen, the a priori positive and negative behaviors did not overlap in rated valence, confirming our original valence assignments.

To assess the predictors of a priori and rated valence, we applied our standard regression analysis pipeline, except that logistic regression was performed for the binary dependent variable of a priori valence. Table SM-3 presents the main effects from these two analyses. As the top half of Table SM-3 illustrates, approximately 100% of the variance in our a priori valence assignments was explained by long-term reward and social approval (because long-term reward and social approval correlated .61, removing either one from their respective Model 2 produced a -1% value in ΔR^2). These results suggest that long-term reward and social approval drove our a priori judgments of valence.

Interestingly, prediction of rated valence reflected many more factors (Table SM-3, bottom half). When participants rated valence themselves, social approval became by far the most important predictor. Long-term reward was the second strongest predictor but was much weaker than social approval. Interestingly, behavior regularity was the third most important predictor, indicating that as participants performed a behavior more regularly, they viewed it as increasingly positive. Similarly, automaticity and immediate reward also explained significant positive variance in rated valence. The importance of regularity, automaticity, and immediate reward suggest that behavior strength and enjoyment induce positive attributions of behaviors.

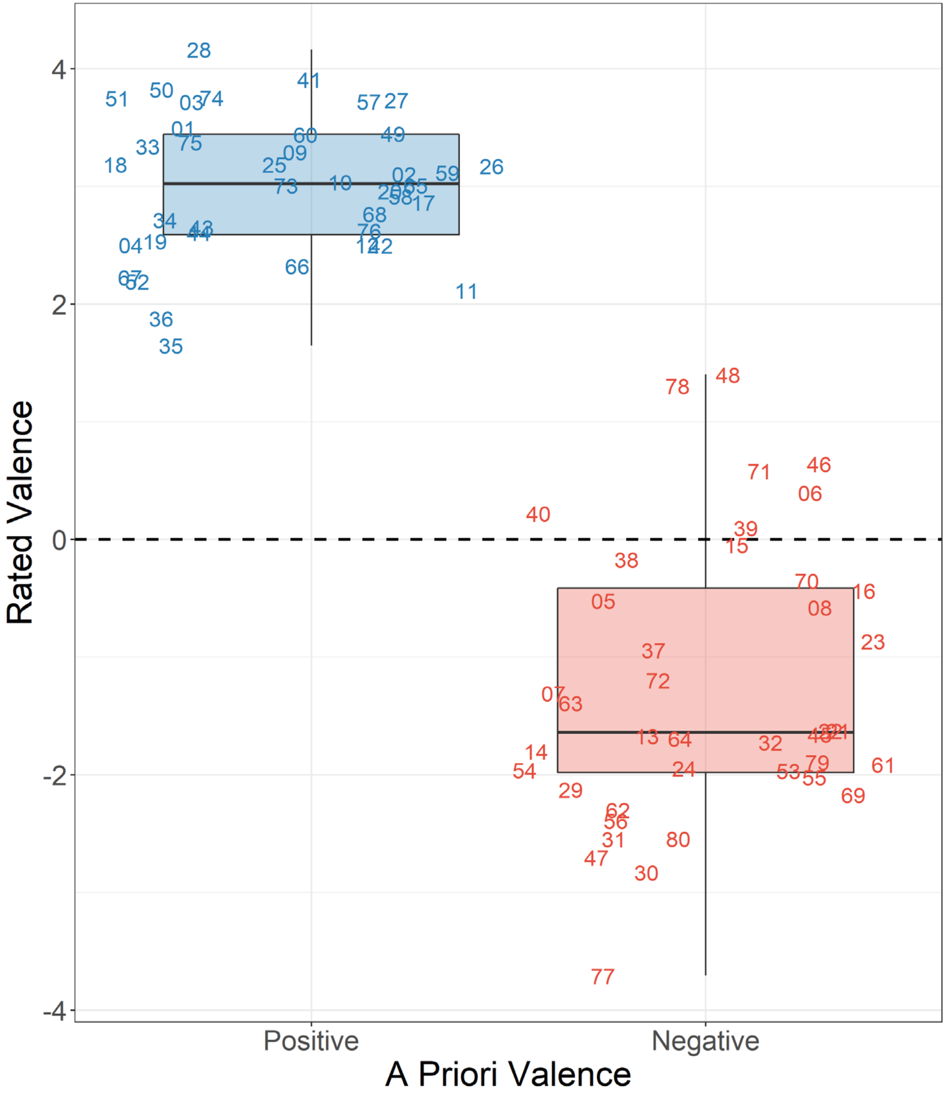

Figure 4. Mean rated valence of the 80 behaviors for habits assigned to a priori positive and negative groups in Table 1 (Study 2). Mean valence is shown in the original scale units (-5 to +5). The numeric identifiers for specific behaviors map onto to the numbers in Table 1, such that specific behaviors in Table 1 can be identified here.

Table SM-3. Results from the group regressions in Study 2 for the prediction of a priori valence (top) and rated valence (bottom) at the behavior level, including predictors for regularity, consistency, immediate reward, long-term reward, conflict, automaticity, and social approval. Section 4 of the SM describes the analysis pipeline used to produce Models 1, 2, and 3.

	**Model 1**			**Model 2**					**Model 3**	
**DV / Predictor**	**Estimate**	**SE**	***t***	**Estimate**	**SE**	***t***	R^2	**AIC**	ΔR^2	**ΔAIC**
**DV: A priori valence**										
Regularity	.10	.269	0.35							
Consistency	-.05	.230	-0.22							
Immediate Reward	-.27	.232	-1.15							
Long-term Reward	.89	.235	3.79*	.89	.235	3.79*	100	228	-1	-7
Conflict	-.16	.174	-0.90							
Automaticity	-.10	.213	-0.47							
Social Approval	1.06	.214	4.94*	1.06	.214	4.94*	99	228	-1	-5
**DV: Rated valence**										
Regularity	.13	.007	18.14*	.13	.016	8.24*	67	24609	-3	841
Consistency	-.02	.006	-2.87*	-.02	.011	-2.15*	66	24956	-1	178
Immediate Reward	.05	.006	7.91*	.04	.012	3.47*	67	24727	-2	461
Long-term Reward	.19	.007	27.95*	.18	.016	11.34*	68	24009	-5	1867
Conflict	-.01	.005	-2.76*	-.01	.008	-1.67	66	24967		
Automaticity	.09	.006	15.46*	.09	.014	6.41*	67	24695	-2	667
Social Approval	.43	.007	64.53*	.44	.023	19.02*	70	23427	-13	5310

Note. All regressions were performed on standardized measures. Thus, an Estimate is the estimate of a standardized regression coefficient in the respective model, with SE and *t*, being the standard error and *t* value of the estimate. R^2 is the total variance explained by Model 2, and ΔR^2 is the amount of variance explained by the main effect or interaction dropped in Model 3 (both in percentage points). AIC is the value of the Akaike Information Criterion for Model 2, and ΔAIC is its change for Model 3. *t* values larger than |1.96| in mixed-effect regressions are statistically significant at approximately $p<.05$ uncorrected (indicated with *).

6. Clustered heatmaps for individual × behavior interactions

A clustered heatmap of the individual × behavior interaction was presented for Study 2 in the main text (Figure 3). Figures SM-7 and SM-8 next present analogous clustered heatmaps for the individual × behavior interactions in Studies 1-all and 3, respectively. See the figure captions and the main article for further details.

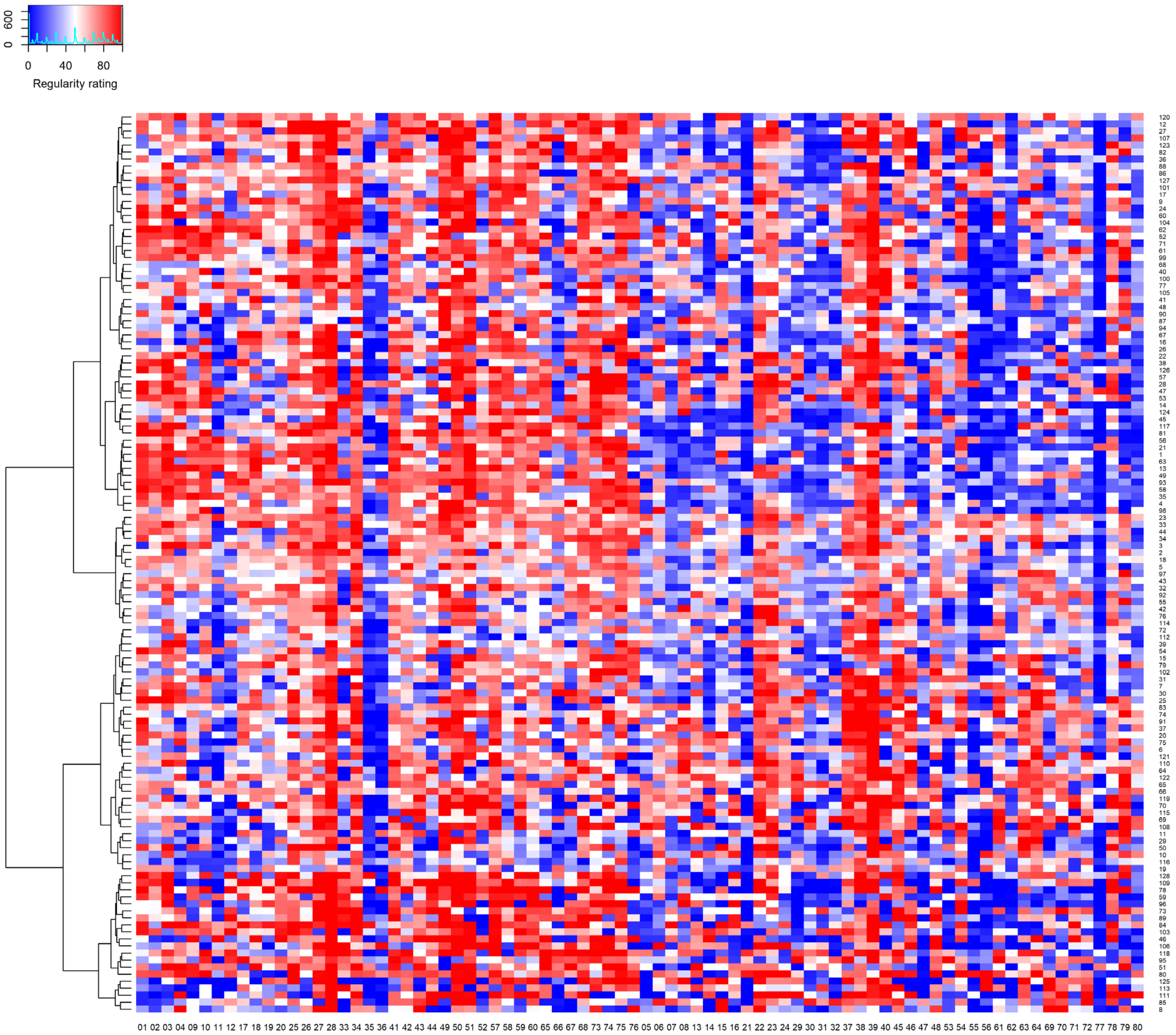

Figure SM-5. A heatmap that visualizes the individual × behavior interaction for behavior regularity in Study 1-all. The 80 regularity judgments for each of the 128 participants are presented in a single row, with their judgments for positive behaviors in the left half, and their judgments for negative behaviors in the right half. The number below each column corresponds to the number of the corresponding behavior in Table 1. As a cell becomes increasingly red, the regularity judgment increasingly approached 100 (on a scale of 0 to 100). As a cell becomes increasingly blue, the regularity judgment increasingly approached 0. As a cell becomes increasingly white, the regularity judgment was increasingly approached 50 . On the left, a hierarchical clustering dendrogram establishes participants having similar vectors of regularity values across situations (from hierarchical clustering with the Ward D measure). Table 1 in the main text provides intraclass correlations that assess inter-rater reliability of the judgments in this map.

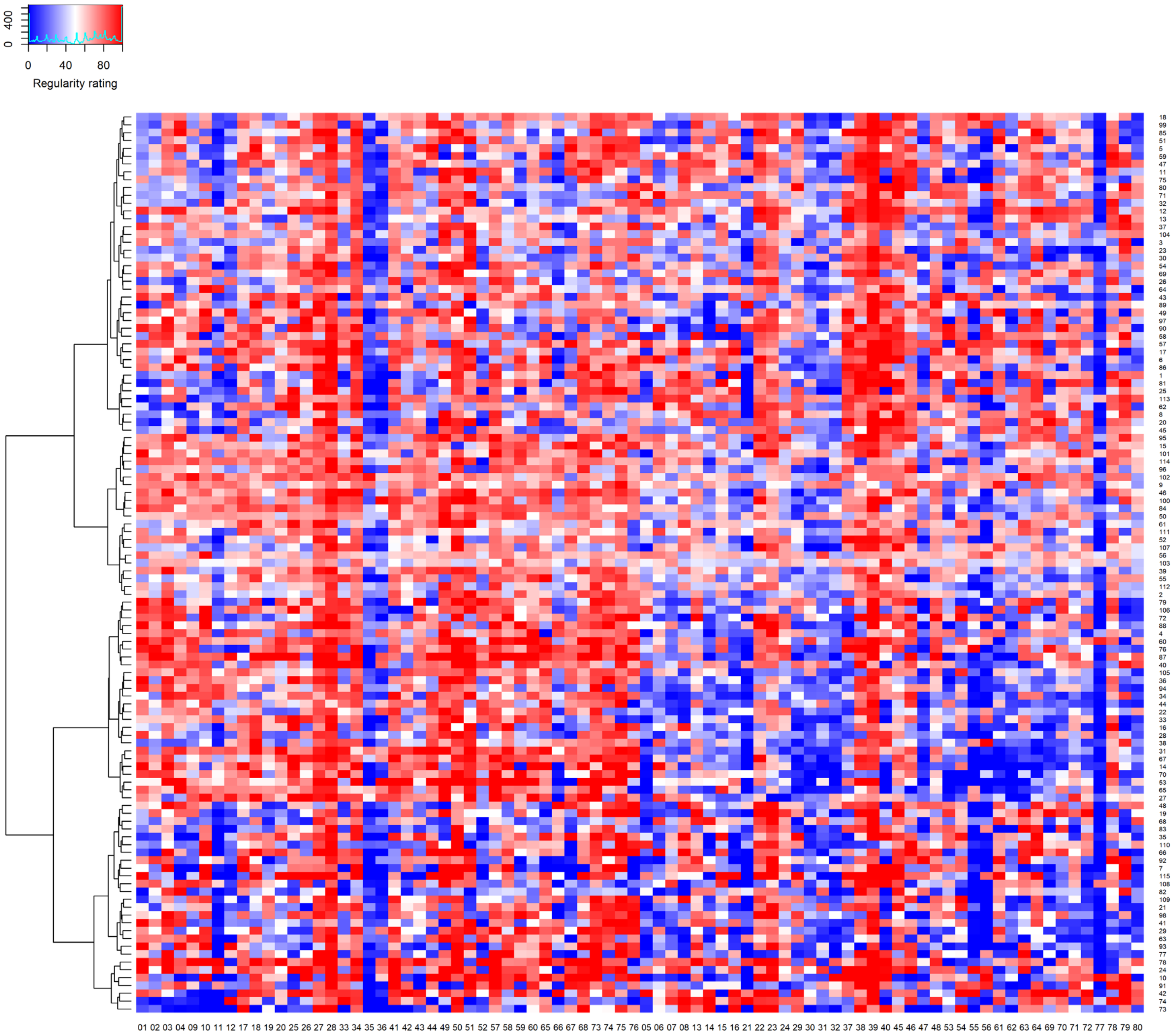

Figure SM-6. A heatmap that visualizes the individual × behavior interaction for behavior regularity in Study 3. The 80 regularity judgments for each of the 115 participants are presented in a single row, with their judgments for positive behaviors in the left half, and their judgments for negative behaviors in the right half. The number below each column corresponds to the number of the corresponding behavior in Table 1. As a cell becomes increasingly red, the regularity judgment increasingly approached 100 (on a scale of 0 to 100). As a cell becomes increasingly blue, the regularity judgment increasingly approached 0. As a cell becomes increasingly white, the regularity judgment was increasingly approached 50 . On the left, a hierarchical clustering dendrogram establishes groups of participants having similar vectors of regularity values across situations (from hierarchical clustering with the Ward D measure). Table 1 in the main text provides intraclass correlations that assess inter-rater reliability of the judgments in this map.

7. Regression results for the group-level analyses of Studies 1-all, 2, and 3

The following three tables contain the complete regression results for group-level results presented in the main text.

Table SM-4 presents the regression results for Study 1-all. Table SM-5 presents the regression results for Study 2. Table SM-6 presents the regression results for Study 3.

Section 4 of the SM describes the analysis pipeline used to produce Models 1, 2, and 3 for all three studies.

Table SM-4. Complete results from the group regressions in Study 1-all for the prediction of behavior regularity, including predictors for valence, consistency, immediate reward, long-term reward, conflict, automaticity, self-control, and neuroticism. Results for all main effects are shown, along with two- and three-way interactions in Model 1 where $t > |1.96|$. Section 4 of the SM describes the analysis pipeline used to produce Models 1, 2, and 3.

**DV: regularity**	**Model 1**			**Model 2**					**Model 3**	
**Predictor**	**Estimate**	**SE**	***t***	**Estimate**	**SE**	***t***	R^2	**AIC**	ΔR^2	**AIC**
Valence (V)	.06	.022	2.87*	.06	.024	2.67*	70	16757	-2	16936
Consistency (Cs)	.45	.010	43.38*	.45	.020	22.30*	71	16481	-14	20003
Immediate Reward (I)	.19	.011	16.41*	.19	.017	11.18*	70	16796	-3	17444
Long-term Reward (L)	.10	.011	9.13*	.11	.016	6.74*	70	16752	-2	17025
Conflict (Cf)	-.01	.010	-0.75							
Automaticity (A)	.30	.011	27.04*	.29	.018	16.24*	70	16714	-7	18703
Self-control (S)	.03	.018	1.42							
Neuroticism (N)	.00	.017	-0.26							
V x L	.06	.010	5.61*	.06	.013	4.90*	70	16720	-1	16922
V x Cf	-.02	.009	-2.06*	-.03	.011	-2.47*	71	16703	-2	16895
Cs x L	.04	.010	3.41*	.03	.012	2.14*	73	16143	-4	16902
Cs x A	.05	.007	7.46*	.03	.009	3.14*	74	16029	-5	16946
Cs x S	.02	.007	2.45*	.03	.016	1.61	71	16493		
Cs x N	.02	.007	2.81*	.02	.016	1.50	71	16488		
I x L	-.04	.009	-3.81*	-.03	.012	-2.23*	71	16685	-2	16905
I x Cf	-.04	.007	-5.23*	-.04	.009	-3.82*	71	16747	-2	16918
I x N	-.04	.008	-4.47*	-.03	.011	-2.88*	70	16809	-1	16911
L x A	-.03	.011	-2.42*	-.02	.013	-1.84	72	16573		
L x N	-.03	.010	-3.12*	-.03	.013	-2.18*	70	16761	-1	16900
V x Cs x Cf	-.02	.009	-2.34*	-.03	.010	-2.90*	73	16255	-4	16896

V x Cs x A	-.02	.009	-2.06*	-.01	.010	-1.12	75	15854		
V x I x L	-.03	.009	-2.87*	-.01	.010	-.92	71	16676		
V x L x S	-.02	.010	-1.98*	-.02	.010	-2.20*	70	16726	-1	16895
Cs x I x A	-.04	.006	-6.10*	-.02	.009	-2.37*	76	15834	-7	16928
Cs x I x S	.03	.007	4.51*	.01	.008	1.45	73	16290		
Cs x L x Cf	.02	.009	2.47*	.02	.010	2.29*	73	16209	-4	16897
Cs x Cf x A	.02	.006	3.87*	.02	.009	2.06*	75	16003	-6	16906
Cs x A x N	.02	.007	3.45*	.01	.008	1.75	74	16040		
Cs x S x N	.02	.008	2.96*	.02	.016	1.39	71	16492		
I x Cf x A	-.02	.006	-3.42*	-.03	.007	-3.74*	72	16586	-3	16902
I x Cf x S	-.02	.006	-2.61*	-.01	.008	-1.69	71	16760		
I x A x N	-.03	.008	-3.22*	-.02	.009	-2.35*	72	16613	-3	16901
Cf x A x S	.02	.007	2.59*	.02	.007	2.10*	71	16703	-2	16897

Note. All regressions were performed on standardized measures. Thus, an Estimate is the estimate of a standardized regression coefficient in the respective model, with SE and *t*, being the standard error and *t* value of the estimate. R^2 is the total variance explained by Model 2, and ΔR^2 is the amount of variance explained by the main effect or interaction dropped in Model 3 (both in percentage points). AIC is the value of the Akaike Information Criterion for Models 2 and 3. *t* values larger than |1.96| in mixed-effect regressions are statistically significant at approximately $p<.05$ uncorrected (indicated with *).

Table SM-5. Complete results in Study 2 from the replication of the group regressions in Study 1 for the prediction of behavior regularity, including predictors for a priori valence, consistency, immediate reward, long-term reward, conflict, automaticity, self-control, and neuroticism. Results for all main effects are shown, along with two- and three-way interactions in Model 1 where $t > |1.96|$. Section 4 of the SM describes the analysis pipeline used to produce Models 1, 2, and 3.

**DV: regularity**	**Model 1**			**Model 2**					**Model 3**	
**Predictor**	**Estimate**	**SE**	***t***	**Estimate**	**SE**	***t***	R^2	**AIC**	ΔR^2	**AIC**
Valence (V)	.03	.022	1.48							
Consistency (Cs)	.44	.008	52.97*	.45	.02	22.14*	69	26809	-10	30274
Immediate Reward (I)	.12	.009	13.22*	.12	.015	8.02*	67	27497	-2	27878
Long-term Reward (L)	.10	.009	11.85*	.11	.015	7.36*	68	27442	-2	27843
Conflict (Cf)	.01	.008	1.24							
Automaticity (A)	.30	.008	35.35*	.29	.016	18.60*	67	27418	-4	28
Self-control (S)	.01	.017	0.33							
Neuroticism (N)	-.01	.017	-0.85							
V x Cf	-.02	.007	-2.40*	-.02	.009	-1.83*	68	27363		
V x S	.02	.008	2.82*	.02	.011	2.11*	67	27501	-1	27712
Cs x I	.02	.006	2.55*	.01	.008	0.69*	71	26213		
Cs x L	.06	.008	7.87*	.02	.01	2.35*	71	26239	-5	27766
Cs x A	.04	.006	7.75*	.01	.011	0.54*	72	25649		
Cs x S	.03	.006	5.12*	.03	.015	2.29*	69	26797	-3	27730
I x L	-.02	.007	-2.46*	-.02	.01	-1.97*	68	27319	-2	27710
I x A	-.02	.006	-3.36*	-.02	.009	-2.49*	68	27133	-2	27715
I x N	-.02	.007	-2.82*	-.02	.01	-1.96*	67	27514		
L x Cf	-.03	.007	-4.58*	-.03	.01	-3.21*	68	27323	-2	27725
L x A	-.05	.008	-5.65*	-.03	.012	-2.77*	69	27085	-3	27736

Cf x S	.02	.006	2.51*	.01	.009	1.25	67	27569		
A x S	-.03	.006	-4.46*	-.03	.011	-2.56*	67	27411	-1	27724
Cs x I x L	.03	.006	4.91*	.02	.007	2.31*	72	26240	-6	27728
Cs x I x A	-.03	.005	-5.14*	-.02	.006	-2.54*	74	25776	-8	27730
Cs x I x S	-.02	.006	-3.78*	-.02	.007	-2.50*	71	26454	-5	27718
Cs x I x N	-.03	.006	-4.61*	-.02	.007	-2.48*	71	26471	-5	27725
Cs x L x Cf	-.02	.007	-3.18*	-.01	.007	-0.83	73	26210		
Cs x L x A	-.02	.007	-2.20*	-.01	.008	-0.89	74	25687		
Cs x Cf x A	.02	.005	4.34*	.01	.006	1.30	73	25921		
Cs x Cf x S	-.03	.005	-4.95*	-.01	.007	-1.67	70	26604		
Cs x A x N	.01	.006	2.25*	-.01	.007	-0.88	72	26068		
Cs x S x N	-.01	.005	-2.41*	-.02	.012	-1.32	69	26840		
I x L x Cf	.02	.007	2.52*	.02	.008	2.82*	69	27230	-3	27710
I x L x A	-.02	.006	-3.44*	-.01	.007	-1.80	70	27066		
I x L x S	.02	.008	2.12*	.01	.008	1.64	68	27331		
I x Cf x A	-.02	.005	-3.13*	-.01	.006	-1.74	69	27153		
I x Cf x S	.01	.006	2.21*	.01	.006	1.94	68	27410		
L x S x N	.01	.006	2.09*	.01	.009	1.56	68	27457		
Cf x A x S	.01	.006	2.52*	.01	.007	2.08*	68	27324	-2	27710
A x S x N	.01	.005	2.06*	.01	.008	1.00d	67	27446		

Note. All regressions were performed on standardized measures. Thus, an Estimate is the estimate of a standardized regression coefficient in the respective model, with SE and *t*, being the standard error and *t* value of the estimate. R^2 is the total variance explained by Model 2, and ΔR^2 is the amount of variance explained by the main effect or interaction dropped in Model 3 (both in percentage points). AIC is the value of the Akaike Information Criterion for Models 2 and 3. *t* values larger than |1.96| in mixed-effect regressions are statistically significant at approximately $p<.05$ uncorrected (indicated with *).

Table SM-6. Complete results from the group regressions in Study 3 for the prediction of behavior regularity, including predictors for valence, consistency, immediate reward, long-term reward, conflict, automaticity, self-control, and neuroticism. Results for all main effects are shown, along with two- and three-way interactions in Model 1 where $t > |1.96|$. Section 4 of the SM describes the analysis pipeline used to produce Models 1, 2, and 3.

**DV: regularity**	**Model 1**			**Model 2**					**Model 3**	
**Predictor**	**Estimate**	**SE**	***t***	**Estimate**	**SE**	***t***	R^2	**AIC**	ΔR^2	**AIC**
Valence	.05	.025	2.04*	.04	.028	1.29	65	16387		
Consistency	.37	.013	27.67*	.37	.020	18.54*	64	16471	-9	18312
Immediate Reward	.15	.012	12.18*	.15	.018	8.39*	64	16576	-2	16792
Long-term Reward	.11	.012	9.02*	.12	.017	6.69*	65	16450	-3	16754
Conflict	-.02	.011	-2.02*	-.02	.012	-1.86	63	16619		
Automaticity	.30	.013	22.14*	.32	.022	14.91*	65	16315	-7	17748
Self-control	-.03	.016	-1.91							
Neuroticism	.03	.016	1.78							
V x Cf	-.02	.010	-2.35*	-.036	.011	-3.26*	65	16388	-2	16617
V x S	.03	.010	2.96*	.021	.017	1.23	65	16379		
Cs x L	.03	.012	2.68*	.016	.014	1.14	66	16286		
Cs x Cf	-.03	.009	-3.23*	-.023	.011	-2.08*	65	16477	-1	16622
Cs x N	-.02	.009	-2.31*	-.018	.013	-1.37	64	16475		
I x Cf	-.02	.007	-2.94*	-.018	.008	-2.21*	64	16601	-1	16620
I x S	-.02	.007	-2.38*	-.022	.011	-2.13*	64	16577	-1	16617
I x N	-.02	.008	-2.77*	-.019	.010	-1.98*	64	16587	-1	16619
L x A	-.06	.013	-4.35*	-.041	.014	-2.96*	67	16153	-4	16630
A x S	.03	.009	3.64*	.021	.016	1.35	65	16328		
A x N	.02	.008	2.57*	.021	.015	1.35	65	16336		
V x Cs x A	-.03	.011	-2.30*	-.001	.012	-0.06	69	15951		

V x I x L	-.04	.010	-3.65*	-.031	.011	-2.94*	66	16361	-2	16625
V x I x N	.03	.011	2.84*	.018	.011	1.63	65	16373		
V x L x Cf	.03	.010	3.16*	.029	.010	2.90*	65	16386	-2	16621
V x L x A	.03	.013	2.28*	.022	.013	1.65	67	16120		
V x L x N	-.04	.010	-3.95*	-.030	.011	-2.84*	65	16375	-2	16627
V x A x N	-.03	.012	-2.14*	-.021	.012	-1.71	66	16132		
Cs x I x Cf	.02	.008	2.25*	.019	.008	2.40*	65	16460	-2	16616
Cs x I x A	-.04	.007	-5.79*	-.026	.007	-3.73*	68	16127	-5	16645
Cs x L x Cf	-.03	.011	-2.47*	-.015	.012	-1.32	67	16303		
Cs x L x A	-.03	.010	-2.47*	-.021	.010	-2.02*	69	15991	-6	16617
Cs x Cf x N	.03	.008	3.29*	.023	.008	2.77*	65	16490	-2	16622
L x Cf x A	.03	.011	2.79*	.028	.012	2.47*	66	16193	-3	16619
L x Cf x S	.02	.009	2.25*	.025	.010	2.65*	65	16462	-2	16616
Cf x A x N	-.02	.008	-2.78*	-.017	.009	-2.01*	65	16329	-2	16619
A x S x N	.02	.009	2.05*	.020	.016	1.28	65	16328		

Note. All regressions were performed on standardized measures. Thus, an Estimate is the estimate of a standardized regression coefficient in the respective model, with SE and *t*, being the standard error and *t* value of the estimate. R^2 is the total variance explained by Model 2, and ΔR^2 is the amount of variance explained by the main effect or interaction dropped in Model 3 (both in percentage points). AIC is the value of the Akaike Information Criterion for Models 2 and 3. *t* values larger than |1.96| in mixed-effect regressions are statistically significant at approximately $p<.05$ uncorrected (indicated with *).

8. Clustered heatmaps of individual predictive correlations

A clustered heatmap of individual predictive correlations was presented for Study 2 in the main text (Figure 6). Figures SM-7 and SM-8 present analogous clustered heatmaps for individual predictive correlations in Studies 1-all and 3, respectively. See the figure captions and the main article for further details.

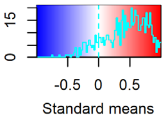

Figure SM-7. A heatmap that visualizes correlations between behavior regularity and individual factors from the Situated Action Cycle (Conflict, Immediate Reward, Long-Term Reward, Automaticity, and Consistency). The six correlations for each of the 128 participants in Study 1-all appear in a single row. As a cell becomes increasingly red, the correlation was increasingly positive. As a cell becomes increasingly blue, the correlation was increasingly negative. As a cell becomes increasingly white, the correlation increasingly approached 0. On the left, a hierarchical clustering dendrogram establishes groups of participants having similar prediction vectors (from hierarchical clustering with the Ward D measure). Table 4 in the main text summarizes the correlations shown below.

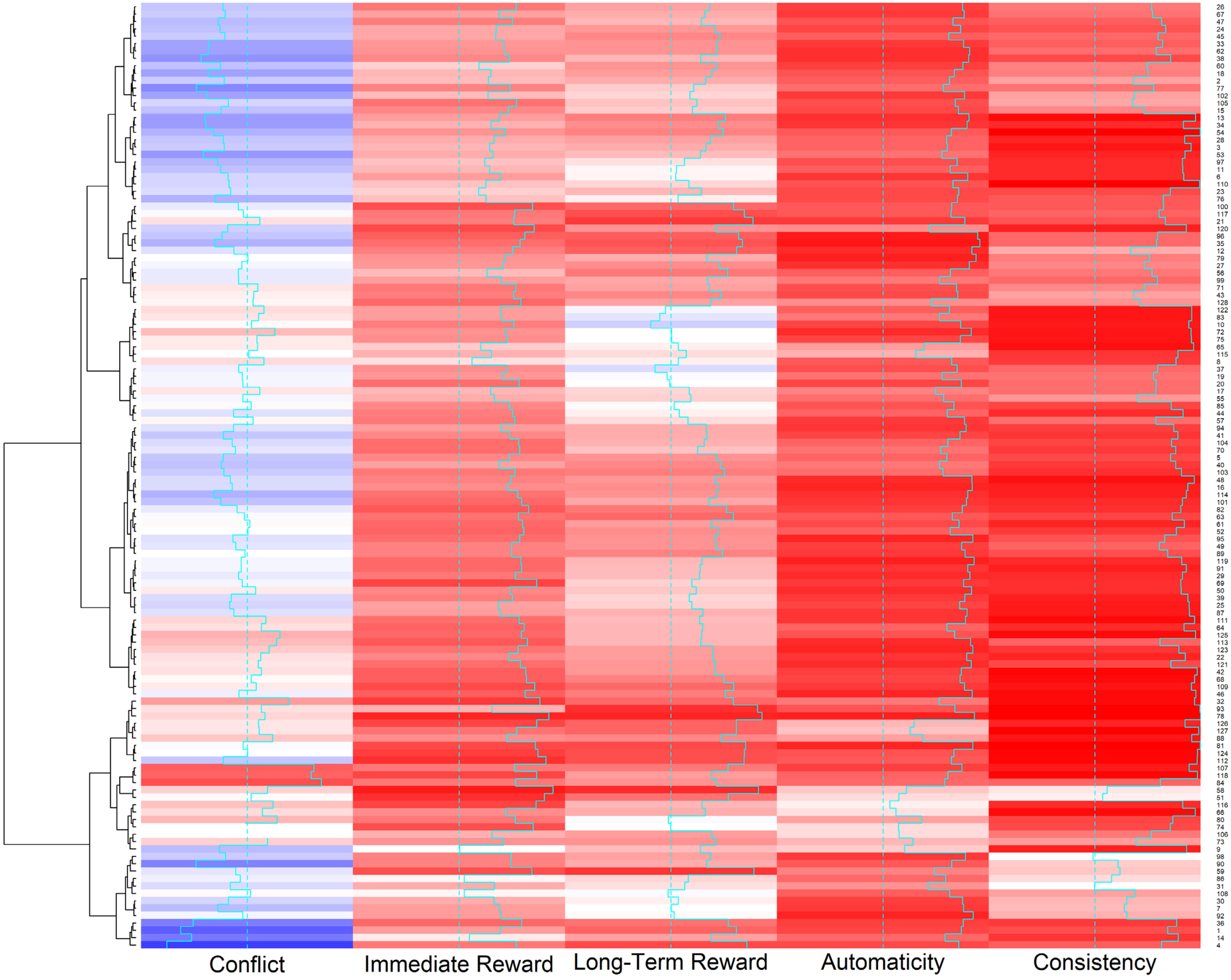

Figure SM-8. A heatmap that visualizes correlations between behavior regularity and individual factors from the Situated Action Cycle (Conflict, Immediate Reward, Long-Term Reward, Automaticity, and Consistency). The six correlations for each of the 115 participants in Study 3 appear in a single row. As a cell becomes increasingly red, the correlation was increasingly positive. As a cell becomes increasingly blue, the correlation was increasingly negative. As a cell becomes increasingly white, the correlation increasingly approached 0. On the left, a hierarchical clustering dendrogram establishes groups of participants having similar prediction vectors (from hierarchical clustering with the Ward D measure). Table 4 in the main text summarizes the correlations shown below.

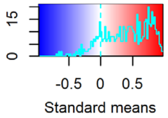

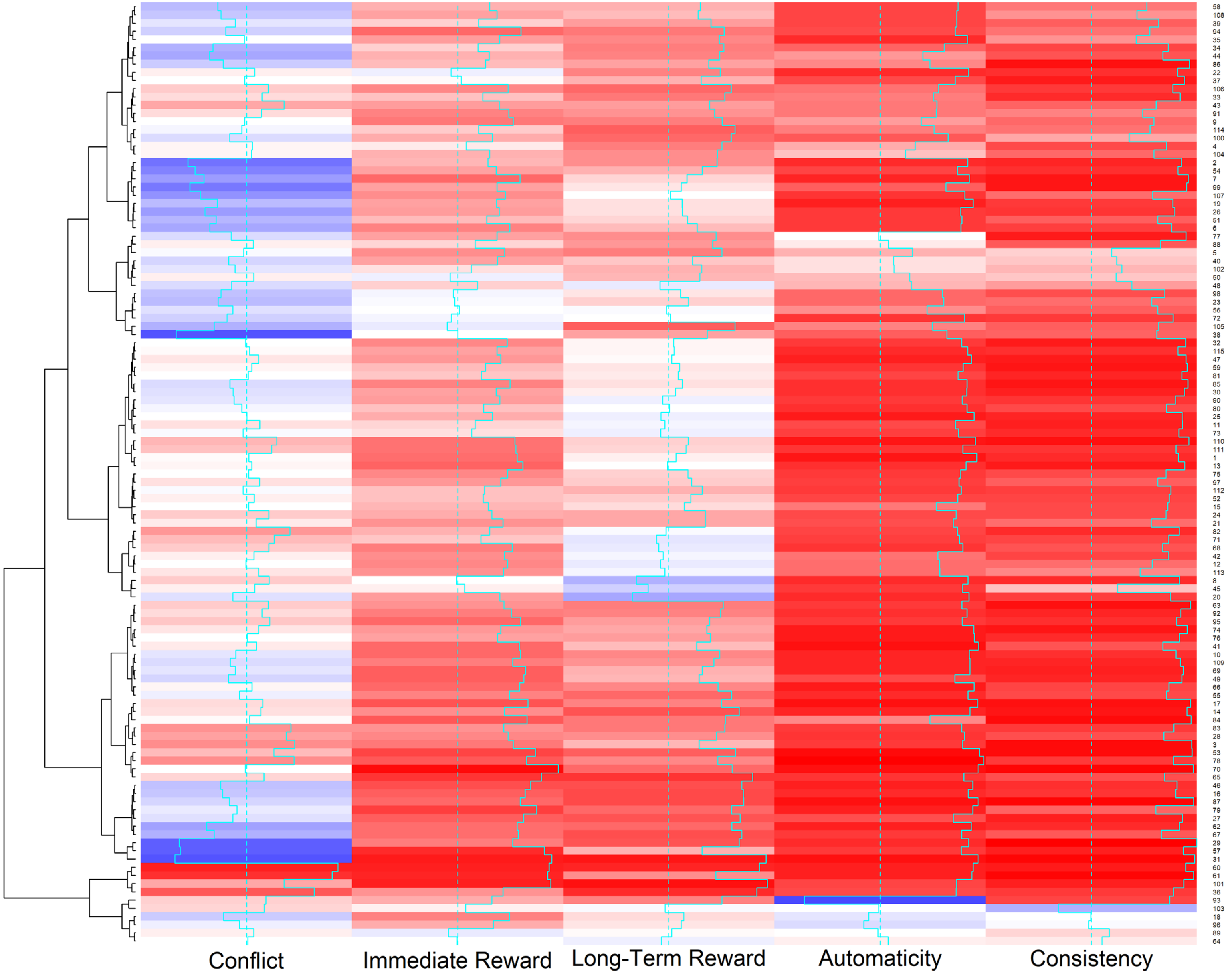

9. Regression analyses that assessed self-control and neuroticism

These next regression analyses produced the results that were displayed in Figure 7 and 8 of the main text.

To test the predicted valence × self-control interaction (Figure 7), we modeled behavior regularity with valence, self-control, and their interaction as predictors. Analogously, to test the predicted valence × neuroticism interaction, we modeled behavior regularity with valence, neuroticism, and their interaction as predictors (Figure 8). Both analyses used the analysis pipeline in Section 4 of the SM to establish Model 1, Model 2, and Model 3 for Studies 1-all, 2, and 3.

The results are shown in the rows of Table SM-7 labelled "V × S (partial)" and "V × N (partial)"). Estimates of the standardized regression coefficients in Model 2 and ΔR^2 in Model 3 provide two measures of effect size. As can be seen, each predicted interaction survived maximal testing in Model 2 for all three studies, explaining 5% to 6% unique variance in behavior regularity.

Table SM-7. Results for the valence x self-control interactions (V x S) and valence x neuroticism interactions (V x N) from group regressions in Studies 1-all, 2, and 3. The partial models predicted behavior regularity with only valence and self-control or neuroticism, whereas the full models further added consistency, immediate reward, long-term reward, conflict, and automaticity (in Study 2, social approval was included as well). See the text for further details. Section 4 of the SM describes the analysis pipeline used to produce Models 1, 2, and 3.

**DV: regularity**		**Model 1**			**Model 2**					**Model 3**	
**Study**	**Predictors**	**Estimate**	**SE**	***t***	**Estimate**	**SE**	***t***	R^2	**AIC**	ΔR^2	**ΔAIC**
1-all	V x S (partial)	.12	.008	14.89*	.12	.018	6.40	39	24758	-5	495
	V x S (full)	.02	.009	1.86							
2	V x S (partial)	.13	.006	19.31*	.13	.016	7.64*	36	39088	-5	803
	V x S (full)	.03	.009	3.69*	.02	.013	1.93	67	27467		
3	V x S (partial)	.13	.009	15.81*	.13	.021	6.39*	38	22554	-6	553
	V x S (full)	.03	.010	2.96*	.02	.017	1.23	65	16379		
1-all	V x N (partial)	-.09	.008	-11.22*	-.09	.019	-4.69*	39	24795	-5	461
	V x N (full)	-.01	.008	-0.67							
2	V x N (partial)	-.09	.007	-13.88*	-.09	.018	-5.02*	37	39096	-5	799
	V x N (full)	-.00	.010	-0.41							
3	V x N (partial)	-.09	.009	-10.57*	-.09	.023	-3.96*	38	22561	-6	546
	V x N (full)	-.01	.009	-1.25							

Note. All regressions were performed on standardized measures. Thus, an Estimate is the estimate of a standardized regression coefficient in the respective model, with SE and *t*, being the standard error and *t* value of the estimate. R^2 is the total variance explained by Model 2, and ΔR^2 is the amount of variance explained by the main effect or interaction dropped in Model 3 (both in percentage points). AIC is the value of the Akaike Information Criterion for Model 2, and ΔAIC is its change for Model 3. *t* values larger than |1.96| in mixed-effect regressions are statistically significant at approximately $p<.05$ uncorrected (indicated with *).

10. Stepwise regressions to establish processes from the Situated Action Cycle that underlie self-control and neuroticism

These analyses explore the interactions in Figure 7 and 8 reported in the main text, producing the further related results also displayed there in Figures 9 and 10

To establish factors from the Situated Action Cycle that underlie self-control and neuroticism, we added the SAM^2 predictors for consistency, immediate reward, long-term reward, conflict, and automaticity to the “partial” regression models in Table SM-7, thereby creating the “full” models shown there (see Section 9 of the SM). If the interactions just reported in Figures 7 and 8 of the main text disappear, then the SAM^2 predictors explain the variance associated with self-control and neuroticism. If, however, these interactions remain, then these predictors do not explain variance associated with the personality measures.

Table SM-7 in the previous section presents the results of these regressions in the rows labelled “V × S (full)” and “V × N (full)”). As can be seen, both interactions disappeared when all five SAM^2 predictors were added to the regression models, indicating that these predictors explained the variance in self-control and neuroticism (Figure SM-9 and SM-10 plot these interactions from the full models).

We next used stepwise regression to identify the specific SAM^2 mechanisms that explained variance in these interactions. Specifically, each step added the mechanisms, one at a time, into a partial Model 2 and assessed how much doing so decreased the estimated regression coefficient for the interaction of interest. After each step, we added the remaining mechanism that decreased the coefficient the most. Every Model 2 contained random intercepts for behaviors and participants, random slopes for valence, self-control and valence × self-control, no random slopes for the added SAM^2 predictors, and all two-way and three-way interactions. Section 4 of the SM describes the regression procedure in detail

Figure 9 in the main text presents results from the stepwise process for the valence × self-control interaction. Across studies, as self-control increased, automaticity, consistency, long-term reward, and immediate reward explained the increased regularity of positive behaviors and the decreased regularity of negative behaviors. Figure 10 in the main text presents the analogous results for the valence × neuroticism interaction. Across studies, as neuroticism increased, automaticity, long-term reward, and consistency explained the decreased performance of positive behaviors and the increased performance of negative behaviors.

Tables SM-8 and SM-9 here present the statistical details for both stepwise analyses. Specifically, Table SM-8 documents the regression results at each step of the stepwise process when evaluating the valence × self-control interaction, as does Table SM-9 for the valence × neuroticism interaction. See the table headings and the main article for further details.

Figure SM-9. The absence of the interaction of behavior valence with self-control across Studies 1-all, 2, and 3. Each interaction modeled here was established in a mixed-effect regression analysis that predicted behavior regularity as a function of valence and self-control, along with the SAM^2 predictors for consistency, immediate reward, long-term reward, conflict, and automaticity (with all variables standardized prior to analysis). Valence was an a priori variable that contrasted positive versus negative behaviors (Table 1). See the full models in Table SM-7 for analysis details.

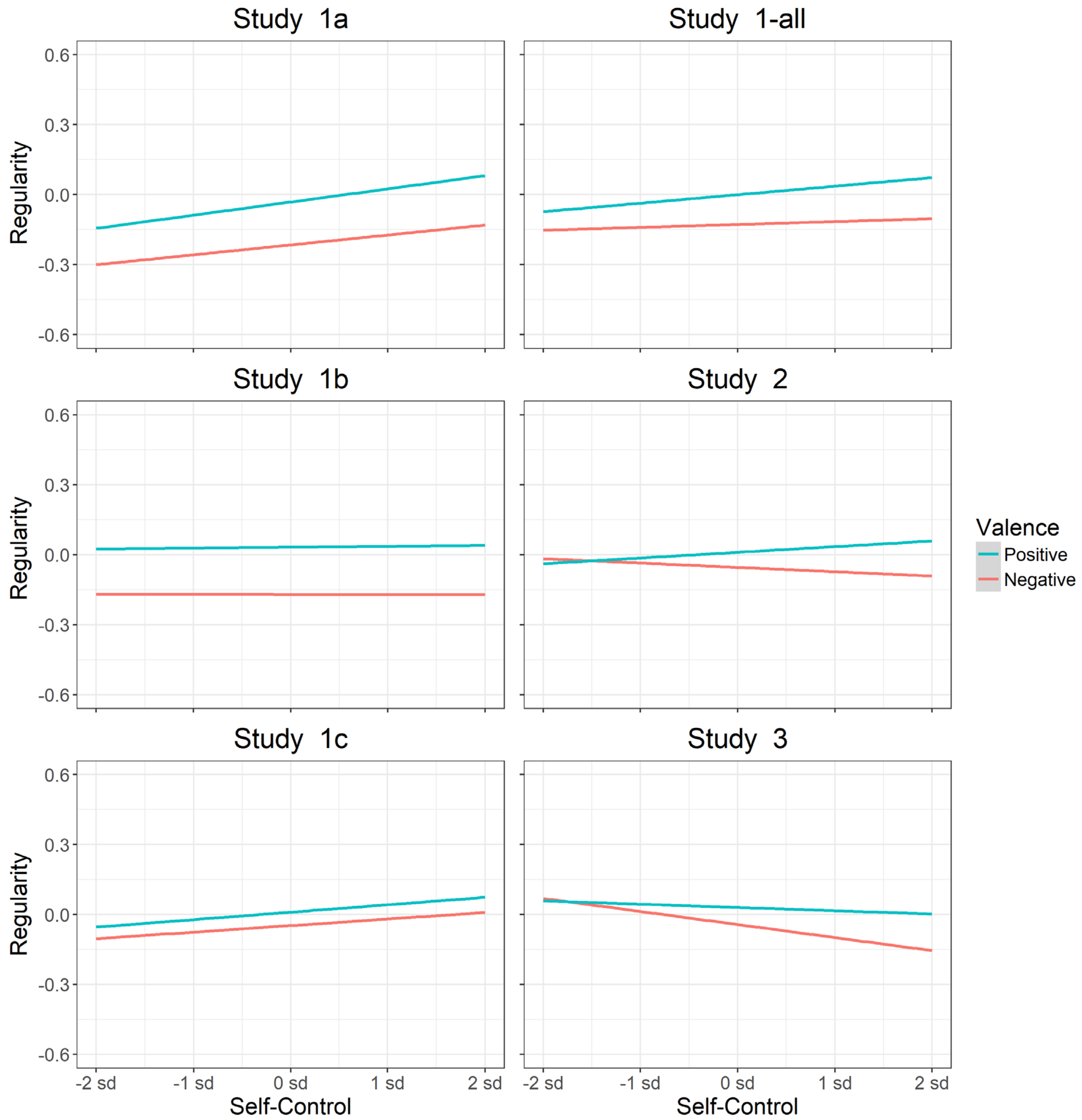

Figure SM-10. The absence of the interaction of behavior valence with neuroticism across Studies 1-all, 2, and 3. Each interaction modeled here was established in a mixed-effect regression analysis that predicted behavior regularity as a function of valence and neuroticism, along with the SAM^2 predictors for consistency, immediate reward, long-term reward, conflict, and automaticity (with all variables standardized prior to analysis). Valence was an a priori variable that contrasted positive versus negative behaviors (Table 1). See the full models in Table SM-7 for analysis details.

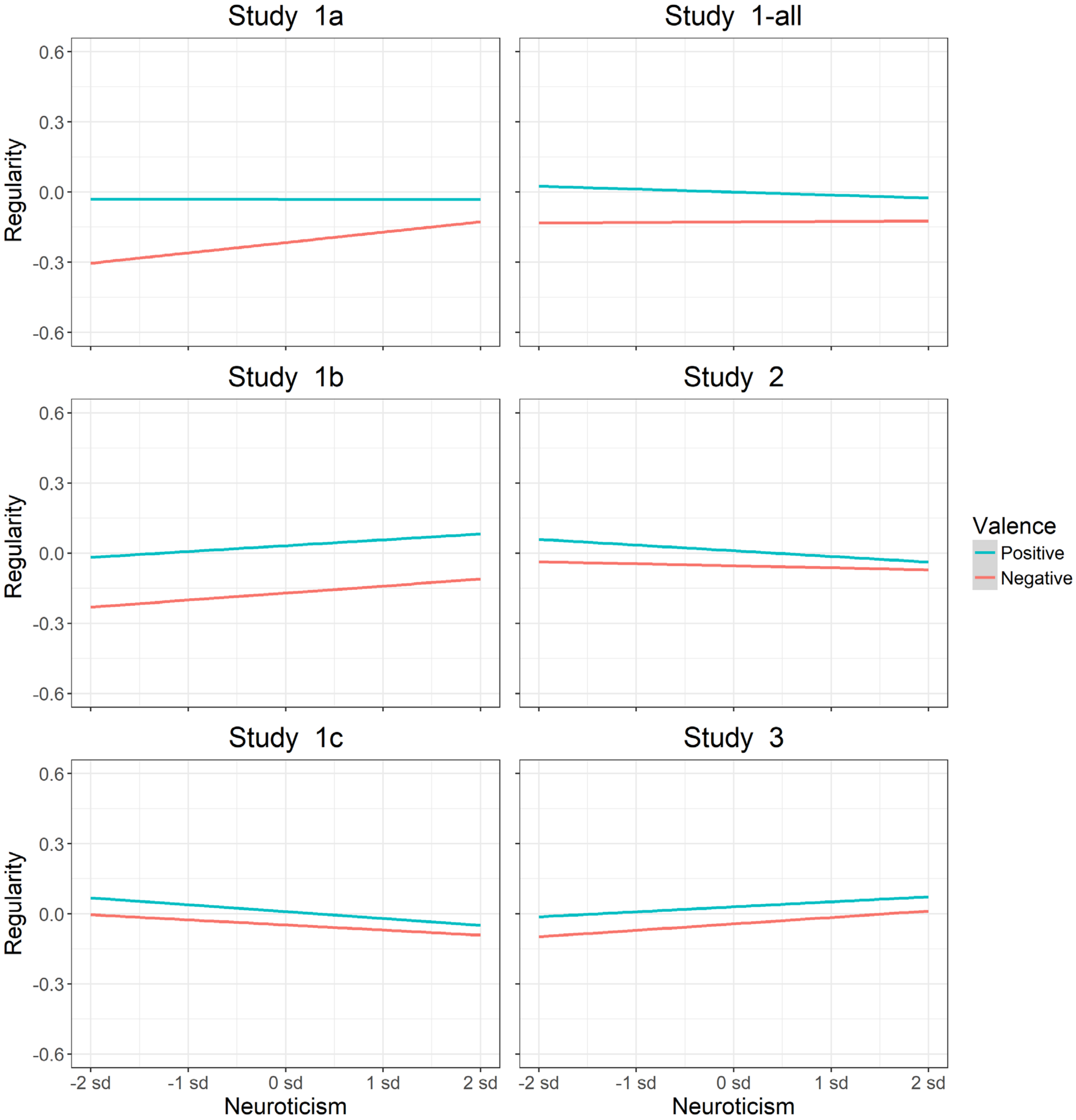

Table SM-8. Results from stepwise regression to establish the SAM^2 predictors that explained variance in the valence × self-control interaction in Studies 1-all, 2, and 3 (Figure 7). In the original analyses (the first row for each study), behavior regularity was regressed onto only valence and self-control in Model 2 from the analysis pipeline in section 4 of the SM to test the valence × self-control interaction maximally. The five primary SAM^2 predictors (Figure 1) were then added one at a time into the original regression to assess how much each alone decreased the coefficient for the valence × self-control interaction ($est_{V \times S}$). The predictor that decreased $est_{V \times S}$ the most is shown in the next row for the study, along with test statistics for the $est_{V \times S}$ interaction and the overall Model 2. In four further iterations of the stepwise process, the remaining SAM^2 predictors were again added one by one to identify the predictor that next decreased the $est_{V \times S}$ interaction the most. These results are shown in the third, fourth, fifth, and sixth rows for each study. Figure 9 in the main text plots each sequence of interactions that resulted from the stepwise analysis. Each model contained random intercepts for participants and behaviors, random slopes for valence, self-control and valence × self-control, no random slopes for the added SAM^2 predictors, and all two-way and three-way interactions.

**DV: regularity**		**V× S interaction**			**Model 2**	
**Study**	**Predictors**	**Estimate**	**SE**	***t***	**R^2**	**AIC**
1-all	Original	.12	.018	6.42*	39	24763
	+ Automaticity	.05	.013	3.72*	52	20962
	+ Immediate	.03	.014	1.98*	57	20118
	+ Consistency	.02	.011	1.66	69	17000
	+ Long-term	.01	.013	1.18	69	16868
	+ Conflict	.02	.013	1.25	70	16789
2	Original	.13	.016	7.64*	36	39088
	+ Consistency	.06	.012	5.45*	56	31995
	+ Automaticity	.04	.009	4.13*	64	28812
	+ Long-term	.03	.010	2.92*	65	28286
	+ Immediate	.02	.010	2.38*	67	27682
	+ Conflict	.03	.010	2.77*	67	27509
3	Original	.14	.022	6.46*	38	22553
	+ Automaticity	.06	.017	3.45*	54	18727
	+ Long-term	.03	.019	1.39	55	18415
	+ Immediate	.02	.019	0.92	58	18083
	+ Conflict	.01	.020	0.57	59	18003
	+ Consistency	.02	.016	1.01	65	16393

Note. All regressions were performed on standardized measures. Thus, an Estimate is the estimate of a standardized regression coefficient in the respective model, with SE and *t*, being the standard error and *t* value of the estimate. R^2 is the total variance explained (in percentage points), and AIC is the value of the Akaike Information Criterion. *t* values larger than |1.96| in mixed-effect regressions are statistically significant at approximately $p<.05$ uncorrected (indicated with *).

Table SM-9. Results from stepwise regression to establish the SAM^2 predictors that explained variance in the valence × neuroticism interaction in Studies 1-all, 2, and 3 (Figure 8). In the original analyses (the first row for each study), behavior regularity was regressed onto only valence and neuroticism in Model 2 from the analysis pipeline in section 4 of the SM to test the valence × neuroticism interaction maximally. The five primary SAM^2 predictors (Figure 1) were then added one at a time into the original regression to assess how much each alone decreased the coefficient for the valence × neuroticism interaction ($est_{V \times N}$). The predictor that decreased $est_{V \times N}$ the most is shown in the next row for the study, along with test statistics for the $est_{V \times N}$ interaction and the overall Model 2. In four further iterations of the stepwise process, the remaining SAM^2 predictors were again added one by one to identify the predictor that next decreased the $est_{V \times N}$ interaction the most. These results are shown in the third, fourth, fifth, and sixth rows for each study. Figure 10 in the main text plots each sequence of interactions that resulted from the stepwise analysis. Each model contained random intercepts for participants and behaviors, random slopes for valence, neuroticism and valence × neuroticism, no random slopes for the added SAM^2 predictors, and all two-way and three-way interactions.

**DV: regularity**		**V× N interaction**			**Model 2**	
**Study**	**Predictors**	**Estimate**	**SE**	***t***	**R^2**	**AIC**
1-all	Original	-.09	.019	-4.70*	39	24795
	+ Long-term	-.05	.020	-2.70*	41	24208
	+ Consistency	-.02	.014	-1.26	60	19660
	+ Automaticity	-.01	.012	-1.07	68	17369
	+ Immediate	-.01	.011	-1.16	69	16823
	+ Conflict	-.01	.012	-0.95	70	16758
2	Original	-.09	.018	-5.02*	37	39096
	+ Automaticity	-.05	.012	-4.08*	48	34159
	+ Consistency	-.03	.009	-3.24*	63	28946
	+ Long-term	-.02	.010	-1.76	65	28390
	+ Immediate	-.02	.010	-1.71	66	27755
	+ Conflict	-.02	.010	-1.68	67	27626
3	Original	-.09	.024	-3.71*	38	22589
	+ Automaticity	-.02	.017	-1.24*	53	18756
	+ Long-term	.00	.018	0.20	55	18457
	+ Consistency	-.01	.015	-0.81	63	16720
	+ Immediate	-.01	.015	-0.56	64	16501
	+ Conflict	-.01	.015	-0.46	65	16438

Note. All regressions were performed on standardized measures. Thus, an Estimate is the estimate of a standardized regression coefficient in the respective model, with SE and *t*, being the standard error and *t* value of the estimate. R^2 is the total variance explained (in percentage points), and AIC is the value of the Akaike Information Criterion. *t* values larger than |1.96| in mixed-effect regressions are statistically significant at approximately $p<.05$ uncorrected (indicated with *).

11. Valence × personality interactions using *rated* valence in Study 2

In the main text, the valence × self-control interaction in Figure 7 and the valence × neuroticism interaction in Figure 8 were both obtained originally using the a priori assignments of valence Table 1. Of interest here is whether these interactions also occur when rated valence replaces a priori valence.

To assess this issue, we modeled behavior regularity with *rated* valence, self-control, and their interaction as predictors. Analogously, to test the predicted valence × neuroticism interaction, we modeled behavior regularity with *rated* valence, neuroticism, and their interaction.

Figure SM-11 displays the valence × self-control interaction and the valence × neuroticism interaction for *rated valence* in Study 2. The analogous interactions with a priori valence from Study 2 are shown again here for the purpose of comparison (i.e., all interactions involving a priori valence were already presented in the main article).

As can be seen, the same interactions occur regardless of whether valence is assigned a priori or rated empirically. See the figure caption and the main article for further details.

Figure SM-11. The behavior valence X self-control interaction in Study 2 for a priori valence (A) and for rated valence (C). The behavior valence X neuroticism interaction in Study 2 for a priori valence (B) and for rated valence (D). The standardized coefficient, β, is shown for each interaction from mixed-effects regression that modeled behavior regularity as a function of valence and either self-control or neuroticism, with no other SAM[2] predictors included.

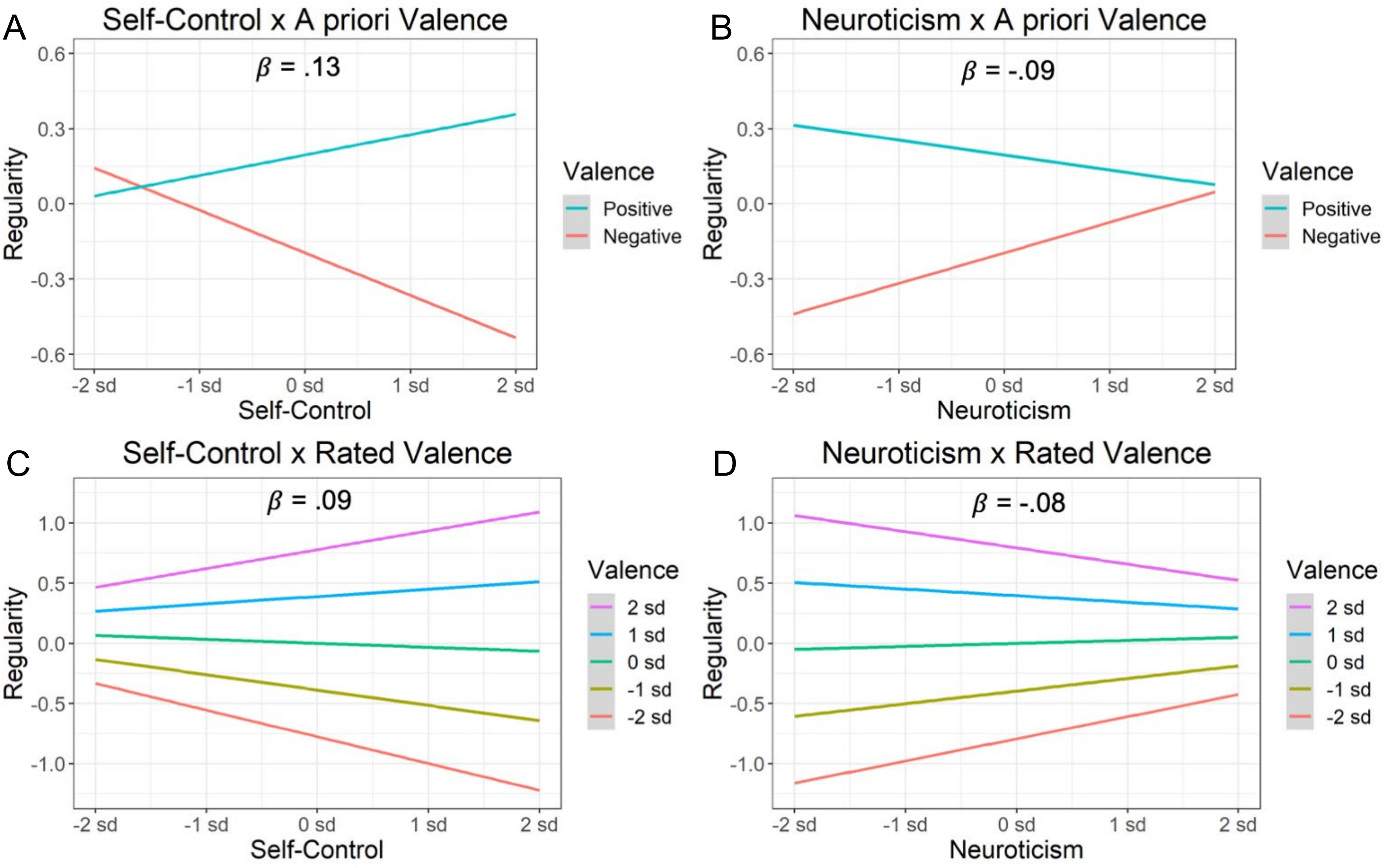

12. Analysis of social approval as a predictor of behavior regularity

Participants in Study 2 rated social approval for each behavior in Table 1. For each behavior participants were asked to judge, "How good or bad do people in general view this behaviour?", using a scale from -5 to 5, with the labels: Very bad, Somewhat bad, Neutral, Somewhat good, Very good. The intraclass correlation for inter-rater agreement was .58 (ICC2).

Of interest is whether including social approval as a predictor improved the prediction of behavior regularity. To assess this issue in Study 2, we replaced a priori valence with rated valence and added social approval to the models in Table SM-5 that previously assessed the prediction of behavior regularity. Both rated valence and social approval were included to assess the roles of these closely related predictors together. Whereas rated valence assessed how *individuals* view positive versus negative behaviors themselves, social approval assessed how individuals believe that *others* view positive versus negative behaviors. Of interest was the relative contributions of these two evaluative judgments to explaining behavior regularity.

Table SM-10 presents the results for the main effects from Models 1, 2, and 3. The top panel of Figure SM-12 presents the estimated regression coefficients from Model 2. Importantly, the overall patterns were highly similar to those in Table SM-5 in the SM and Figure 4 in the main text. Again, consistency and then automaticity were the best predictors of behavior regularity, followed by immediate and then long-term reward, with conflict being unrelated. Interestingly, rated valence was a somewhat stronger predictor of behavior regularity than was a priori valence, suggesting that individuals judged behaviors more positively as they performed them more regularly.

To our surprise, social approval was negatively related to behavior regularity. This result is surprising, given that the correlation between social approval and regularity was positive ($r = .24$). We subsequently discovered that social approval became negative in the regression because of its high collinearity with rated valence ($r = .76$). When rated valence was removed from the regression model, social approval became a positive predictor, indicating that its original negative coefficient resulted from high collinearity. These results indicate that rated valence largely captured the importance of social approval. Social approval does not need to be included if valence is in the predictive model.

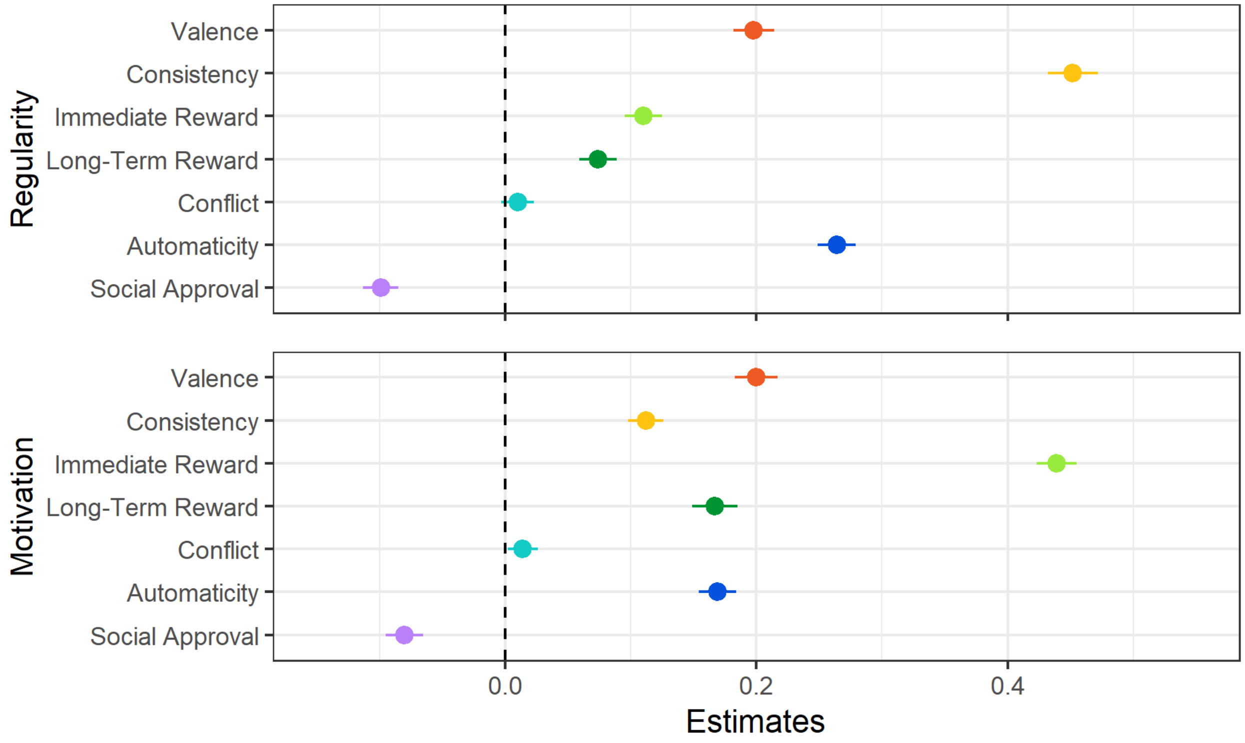

Figure 12. Estimated regression coefficients for the prediction of behavior regularity (top) and behavior motivation (bottom) in Study 2, including predictors for *rated valence*, consistency, immediate reward, long-term reward, conflict, automaticity, and social approval. All coefficients are standardized and were established in Model 2 of the analysis pipeline (see section 4 of the SM for details). The line for each coefficient is its standard error. Tables SM-10 and SM-11 provides details of the relevant regression analyses.

Table SM-10. Complete results from the group regressions in Study 2 for the prediction of behavior regularity, including predictors for rated valence, consistency, immediate reward, long-term reward, conflict, automaticity, social approval, self-control, and neuroticism. Results for all main effects are shown, along with two- and three-way interactions in Model 1 where $t > |1.96|$. Section 4 of the SM describes the analysis pipeline used to produce Models 1, 2, and 3.

**DV: regularity**	**Model 1**			**Model 2**					**Model 3**	
**Predictor**	**Estimate**	**SE**	***t***	**Estimate**	**SE**	***t***	R^2	**AIC**	ΔR^2	**AIC**
Valence (V)	.19	.011	17.60*	.20	.016	12.07*	68	27041	-2	27528
Consistency (Cs)	.45	.008	54.28*	.45	.020	23.01*	69	26347	-10	29910
Immediate Reward (I)	.11	.009	11.87*	.11	.015	7.41*	68	27054	-1	27362
Long-term Reward (L)	.07	.010	7.29*	.07	.016	4.73*	68	27002	-2	27275
Conflict (Cf)	.01	.008	1.61							
Automaticity (A)	.27	.008	32.15*	.26	.015	17.44*	68	26959	-3	28221
Social Approval (So)	-.10	.010	-1.17	-.10	.014	-7.04*	68	27093	-1	27325
Self-control (S)	.00	.016	0.21							
Neuroticism (N)	-.01	.016	-0.65							
V x Cf	-.05	.009	-5.19*	-.05	.010	-4.35*	69	26926	-2	27249
V x A	-.04	.009	-4.16*	-.04	.011	-3.29*	69	26698	-2	27239
V x S	.03	.010	3.14*	.02	.013	1.48	68	27038		
Cs x I	.02	.006	2.41*	.00	.008	0.47	72	25819		
Cs x L	.05	.007	6.91*	.02	.009	2.48*	72	25823	-5	27269
Cs x A	.04	.006	7.30*	.01	.010	0.45	72	25269		
Cs x So	-.02	.009	-2.27*	-.02	.011	-1.75	71	26149		
Cs x S	.03	.006	5.07*	.03	.015	2.30*	70	26340	-3	27248
I x L	-.02	.007	-2.28*	-.02	.010	-2.23*	69	26888	-2	27227
I x A	-.03	.006	-4.16*	-.03	.009	-3.16*	69	26707	-2	27239
I x N	-.02	.007	-2.70*	-.02	.010	-2.00*	68	27064	-1	27229
L x Cf	-.02	.007	-2.61*	-.01	.009	-1.52	69	26902		
L x A	-.03	.008	-3.65*	-.02	.011	-1.88	70	26669		
L x S	.02	.008	1.97*	.01	.012	1.15	69	26993		
Cf x S	.02	.006	2.64*	.01	.009	1.41	68	27104		

Cf x N	.01	.006	2.02*	.01	.010	1.40	68	27115		
A x So	.03	.009	3.78*	.03	.010	3.31*	69	26814	-2	27236
A x S	-.03	.006	-5.15*	-.03	.011	-3.00*	68	26956	-1	27248
So x S	-.02	.009	-2.48*	-.02	.011	-1.54	68	27087		
V x Cs x L	-.04	.008	-4.84*	-.03	.009	-3.13*	73	25872	-6	27245
V x Cs x A	-.05	.007	-6.65*	-.03	.007	-4.29*	74	25328	-8	27266
V x Cs x N	.03	.009	3.54*	.02	.010	1.67	72	26007		
V x I x L	.02	.008	1.99*	.02	.008	2.17*	70	26843	-3	27226
V x Cf x A	-.03	.008	-3.43*	-.03	.008	-3.10*	70	26669	-3	27234
V x Cf x So	-.01	.006	-2.08*	-.01	.007	-1.37	69	26904		
V x Cf x S	.02	.009	2.60*	.02	.010	2.27*	69	26926	-2	27229
V x A x N	-.02	.009	-2.57*	-.03	.011	-2.19*	69	26780	-2	27228
Cs x I x L	.04	.006	6.21*	.02	.007	2.66*	73	25854	-6	27260
Cs x I x A	-.02	.005	-3.82*	-.01	.006	-1.98*	74	25433	-7	27236
Cs x I x S	-.02	.006	-3.66*	-.02	.007	-2.46*	72	26056	-4	27235
Cs x I x N	-.03	.006	-4.76*	-.02	.007	-2.63*	71	26068	-4	27244
Cs x L x Cf	-.01	.006	-2.12*	-.00	.007	-0.21	73	25822		
Cs x L x N	-.02	.008	-2.18*	-.02	.009	-1.89	72	26006		
Cs x Cf x A	.02	.005	4.19*	.01	.006	1.37	73	25543		
Cs x Cf x S	-.03	.005	-4.78*	-.01	.007	-1.73	71	26175		
Cs x A x So	.04	.007	5.32*	.03	.007	3.83*	74	25489	-7	27250
I x L x A	-.03	.006	-4.17*	-.02	.007	-2.47*	70	26668	-3	27239
I x Cf x A	-.01	.005	-2.34*	-.01	.006	-1.42	70	26742		
Cf x A x So	.02	.007	1.98*	.02	.009	1.93	70	26727		

Note. All regressions were performed on standardized measures. Thus, an Estimate is the estimate of a standardized regression coefficient in the respective model, with SE and *t*, being the standard error and *t* value of the estimate. R^2 is the total variance explained by Model 2, and ΔR^2 is the amount of variance explained by the main effect or interaction dropped in Model 3 (both in percentage points). AIC is the value of the Akaike Information Criterion for Models 2 and 3. *t* values larger than |1.96| in mixed-effect regressions are statistically significant at approximately $p<.05$ uncorrected (indicated with *).

13. Predicting behavior motivation

Participants in Study 2 rated social approval for each behavior in Table 1. For each behavior participants were asked to judge, "How strongly are you motivated to perform this behaviour in the relevant situations??", using a scale from -5 to 5, with the labels: Not at all, Somewhat, A lot. The intraclass correlation for inter-rater agreement was .22 (ICC2).

Of interest was establishing factors from the Situated Action Cycle for habitual behavior in Figure 1 that predict behavior motivation and comparing them to the factors that predict behavior regularity. Because motivation often results from anticipated reward, we predicted that immediate and long-term reward would be more important for predicting behavior motivation than for predicting behavior regularity. Additionally, because of the explicit focus on behavior motivation, we predicted that long-term reward would become especially important, exceeding the importance of immediate reward.

To test these predictions in Study 2, we predicted behavior motivation instead of behavior regularity with the same model that included rated valence and social approval in Table SM-10 (top). Table SM-13 and the bottom half of Figure SM-12 present the results.

As predicted, both immediate and long-term reward increased in importance for predicting behavior motivation, relative to predicting behavior regularity. Unexpectedly, however, immediate reward was the most important predictor of motivation (not long-term reward), more than tripling in magnitude, relative to its magnitude for the prediction of regularity. Long-term reward doubled in importance but was about a third as important as immediate reward. As reward became more important for behavior motivation, consistency and automaticity become less important. Interestingly, however, both remained important, suggesting that behavior strength produces impetus for behavior motivation. Again, the co-linearity of social approval and rated valence caused social approval to predict motivation negatively (as found in the previous section for social approval as a predictor of behavior regularity). When rated valence was removed, social approval became positively related to motivation.

Table SM-11. Complete results from the group regressions in Study 2 for the prediction of behavior motivation, including predictors for rated valence, consistency, immediate reward, long-term reward, conflict, automaticity, social approval, self-control, and neuroticism. Results for all main effects are shown, along with two- and three-way interactions in Model 1 where $t > |1.96|$. Section 4 of the SM describes the analysis pipeline used to produce Models 1, 2, and 3.

**DV: motivation**	**Model 1**			**Model 2**					**Model 3**	
**Predictor**	**Estimate**	**SE**	***t***	**Estimate**	**SE**	***t***	R^2	**AIC**	ΔR^2	**AIC**
Valence (V)	.21	.011	19.07*	.20	.017	12.12*	69	27236	-3	28176
Consistency (Cs)	.12	.008	14.08*	.11	.014	8.14*	68	27544	-1	28014
Immediate Reward (I)	.42	.009	45.15*	.44	.016	28.19*	68	27279	-5	29726
Long-term Reward (L)	.14	.010	14.12*	.17	.018	9.47*	70	26782	-4	28015
Conflict (Cf)	.02	.008	2.55*	.01	.012	1.15*	68	27607		
Automaticity (A)	.17	.009	2.21*	.17	.015	11.01*	68	27370	-2	28217
Social Approval (So)	-.08	.010	-8.03*	-.08	.015	-5.20*	69	27285	-1	27881
Self-control (S)	-.00	.015	-0.21							
Neuroticism (N)	.02	.015	1.13							
V x Cs	.02	.010	2.03*	.01	.012	1.19	70	26965		
V x Cf	-.02	.009	-2.16*	-.02	.011	-1.89	70	26927		
Cs x I	-.02	.007	-2.61*	-.02	.009	-2.55*	70	26737	-3	27824
Cs x N	.03	.007	4.07*	.03	.012	2.17*	68	27551	-1	27834
I x L	-.04	.007	-5.93*	-.04	.011	-3.23*	72	26164	-5	27852
I x So	.03	.010	2.91*	.01	.011	1.15	71	26684		
I x S	-.02	.007	-2.41*	-.01	.013	-0.85	68	27297		
I x N	-.06	.007	-8.07*	-.05	.014	-3.55*	68	27280	-1	27882
L x Cf	-.02	.007	-2.55*	.00	.010	0.00	71	26380		
L x A	-.02	.008	-2.08*	.01	.011	0.75	72	26064		
L x S	.03	.008	3.61*	.03	.016	1.55	70	26778		

L x N	-.04	.008	-4.43*	-.03	.016	-1.88	70	26785		
Cf x So	-.02	.008	-2.86*	-.01	.011	-1.22	70	27014		
Cf x S	-.02	.006	-2.73*	-.01	.011	-1.12	68	27601		
Cf x N	-.03	.006	-4.40*	-.02	.011	-1.62	68	27617		
A x S	-.03	.006	-3.92*	-.03	.013	-2.16*	68	27381	-1	27832
V x Cs x So	-.02	.007	-3.19*	-.02	.008	-2.29*	70	26854	-3	27827
V x I x L	-.02	.008	-2.86*	-.03	.009	-2.69*	74	26015	-6	27825
V x I x So	.02	.007	3.45*	.01	.008	0.57	72	26387		
V x I x S	-.03	.010	-2.97*	-.02	.011	-2.22*	72	26459	-4	27826
V x I x N	-.04	.010	-3.77*	-.02	.011	-2.15*	72	26457	-4	27831
V x L x A	.02	.009	2.81*	.03	.010	2.78*	73	25989	-6	27825
V x L x So	.02	.007	3.31*	.01	.008	1.65	72	26571		
V x Cf x So	-.02	.006	-3.44*	-.01	.008	-1.65	71	26862		
V x Cf x N	-.02	.009	-2.63*	-.02	.012	-1.57	70	26978		
V x A x S	.04	.010	4.20*	.03	.011	3.06*	71	26705	-4	27835
V x A x N	.03	.010	3.45*	.02	.011	1.90	71	26709		
V x S x N	.03	.007	4.51*	.03	.013	2.06*	69	27248	-2	27837
Cs x I x L	.02	.006	3.14*	.00	.007	0.12	74	25877		
Cs x I x A	-.01	.005	-2.53*	-.01	.007	-1.04	72	26355		
Cs x I x N	-.03	.006	-4.35*	-.02	.007	-2.57*	70	26889	-3	27836
Cs x L x A	-.02	.007	-3.19*	-.03	.008	-3.35*	73	25942	-6	27827
Cs x L x So	.03	.008	3.64*	.02	.010	1.87	72	26281		
Cs x L x S	.02	.008	2.37*	.02	.009	2.07*	71	26434	-4	27823
Cs x A x N	.02	.006	3.61*	.01	.006	0.85	69	27227		
I x Cf x So	.03	.007	3.77*	.02	.008	2.81*	72	26397	-5	27831
I x A x So	-.03	.008	-3.47*	-.02	.008	-2.13*	73	26007	-6	27829
I x A x S	-.01	.006	-2.32*	-.02	.007	-2.33*	71	26586	-4	27822

I x A x N	-.02	.006	-3.14*	-.02	.007	-2.63*	71	26587	-4	27827
I x So x N	.02	.009	1.96*	.03	.010	2.52*	71	26710	-4	27821
L x A x So	-.02	.009	-2.80*	-.03	.010	-3.07*	73	26039	-6	27825
L x A x N	-.03	.008	-3.14*	-.02	.009	-2.05*	72	26153	-5	27827
A x So x S	-.02	.009	-2.53*	-.02	.010	-2.42*	70	26807	-3	27823

Note. All regressions were performed on standardized measures. Thus, an Estimate is the estimate of a standardized regression coefficient in the respective model, with SE and *t*, being the standard error and *t* value of the estimate. R^2 is the total variance explained by Model 2, and ΔR^2 is the amount of variance explained by the main effect or interaction dropped in Model 3 (both in percentage points). AIC is the value of the Akaike Information Criterion for Models 2 and 3. *t* values larger than |1.96| in mixed-effect regressions are statistically significant at approximately $p<.05$ uncorrected (indicated with *).

14. Assessing explicit awareness of factors that predict behavior regularity

Because habitual behaviors relatively implicit and automatic, participants should be relatively unaware of the specific SAM^2 factors from the Situated Action Cycle in Figure 1 most associated with their individual patterns of behavior regularity. To assess this prediction in Study 3, participants produced 5 responses on a 0 to 100 scale to estimate how much overall influence each factor has on their performance of the 80 behaviors (where the factors were consistency, immediate reward, long-term reward, conflict, and automaticity). Figure SM-13 presents the items used to collect these responses.

For each participant, a second vector of 5 values was created that contained the actual correlations of these 5 factors with behavior regularity in their individual data. These two vectors were then correlated for each participant, indicating how well their prediction estimates correlated with their actual predictive relations.

Figure SM-14 presents the results, with each point being the correlation between the estimated and observed prediction vectors for one of the 115 participants in Study 3. As can be seen, the median correlation across participants was .25, with a broad range from -.85 to +.98, suggesting large individual differences. As we predicted, many participants did not appear to have much awareness of what actually predicted their behavior regularity. No other measure correlated well with these values, suggesting that they may not be systematic (e.g., self-control correlated -.18, neuroticism correlated -.19).

Interestingly, the average estimates across participants departed substantially from the overall relationships between the SAM^2 predictors and behavior regularity actually observed in Figures 7 and 11. Specifically, participants' average estimates (on a 0 to 100 scale) were 63.33 for consistency, 75.45 for immediate reward, 63.01 for long-term reward, 59.29 for conflict, and 75.47 for automaticity. Participants mistakenly perceived immediate and long-term reward as being comparable in importance to consistency and automaticity, when consistency and automaticity were actually much more important. Participants mistakenly believed that conflict was quite important, when actually it was not. Participants mistakenly believed that automaticity was more important than consistency, when consistency was actually more important. These misconceptions indicate that, overall, participants had little awareness of the factors associated with behavior regularity.

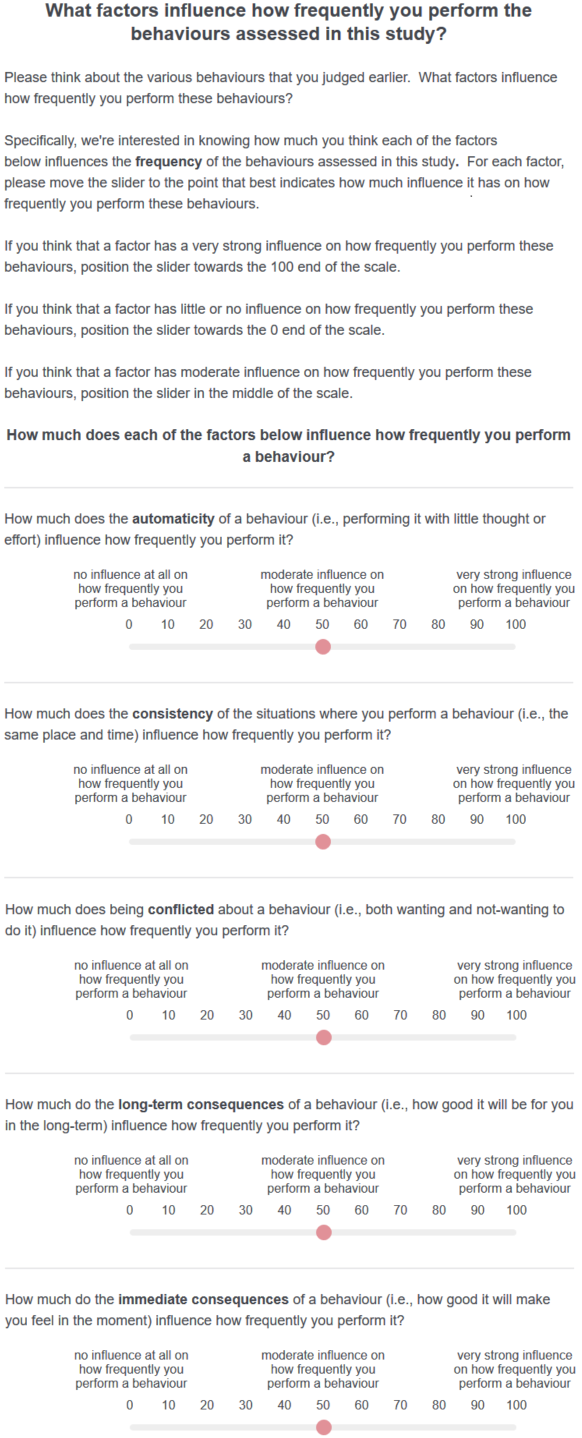
What factors influence how frequently you perform the behaviours assessed in this study?

Please think about the various behaviours that you judged earlier. What factors influence how frequently you perform these behaviours?

Specifically, we're interested in knowing how much you think each of the factors below influences the **frequency** of the behaviours assessed in this study. For each factor, please move the slider to the point that best indicates how much influence it has on how frequently you perform these behaviours.

If you think that a factor has a very strong influence on how frequently you perform these behaviours, position the slider towards the 100 end of the scale.

If you think that a factor has little or no influence on how frequently you perform these behaviours, position the slider towards the 0 end of the scale.

If you think that a factor has moderate influence on how frequently you perform these behaviours, position the slider in the middle of the scale.

How much does each of the factors below influence how frequently you perform a behaviour?

How much does the **automaticity** of a behaviour (i.e., performing it with little thought or effort) influence how frequently you perform it?

no influence at all on how frequently you perform a behaviour | moderate influence on how frequently you perform a behaviour | very strong influence on how frequently you perform a behaviour

0 10 20 30 40 50 60 70 80 90 100

How much does the **consistency** of the situations where you perform a behaviour (i.e., the same place and time) influence how frequently you perform it?

no influence at all on how frequently you perform a behaviour | moderate influence on how frequently you perform a behaviour | very strong influence on how frequently you perform a behaviour

0 10 20 30 40 50 60 70 80 90 100

How much does being **conflicted** about a behaviour (i.e., both wanting and not-wanting to do it) influence how frequently you perform it?

no influence at all on how frequently you perform a behaviour | moderate influence on how frequently you perform a behaviour | very strong influence on how frequently you perform a behaviour

0 10 20 30 40 50 60 70 80 90 100

How much do the **long-term consequences** of a behaviour (i.e., how good it will be for you in the long-term) influence how frequently you perform it?

no influence at all on how frequently you perform a behaviour | moderate influence on how frequently you perform a behaviour | very strong influence on how frequently you perform a behaviour

0 10 20 30 40 50 60 70 80 90 100

How much do the **immediate consequences** of a behaviour (i.e., how good it will make you feel in the moment) influence how frequently you perform it?

no influence at all on how frequently you perform a behaviour | moderate influence on how frequently you perform a behaviour | very strong influence on how frequently you perform a behaviour

0 10 20 30 40 50 60 70 80 90 100

Figure SM-13. The screen in Study 3 used to collect each participant's 5 estimates of how much each of the SAM^2 predictors affect how often they perform the 80 behaviors they had just judged in the five previous blocks of the study. The five scales were randomized for each participant.

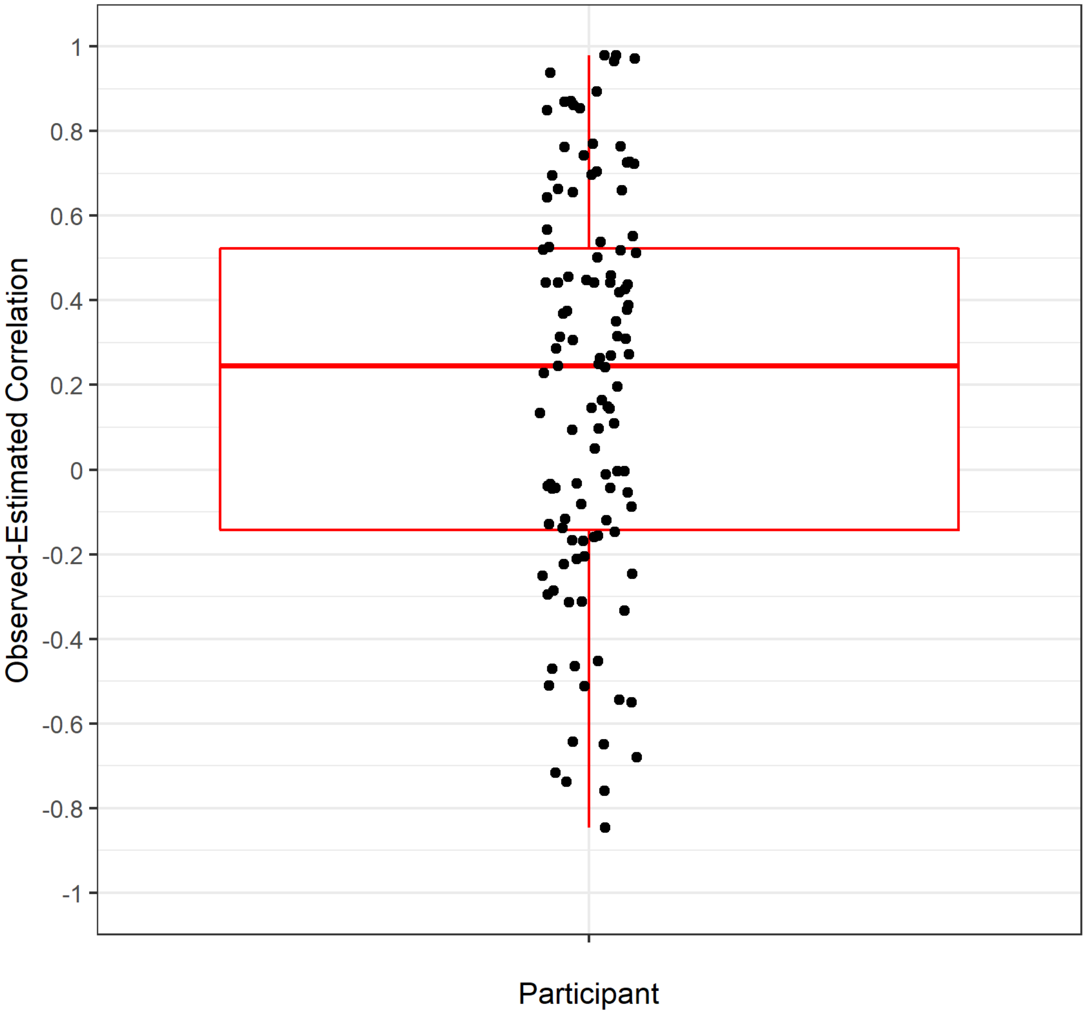

Figure SM-14. Individual correlations for participants between their estimated prediction vectors and their observed prediction vectors (Study 3). Specifically, each point represents the correlation between: (1) a participant's estimates of how much overall influence each of the 6 predictors had on their regularity of performing the 80 behaviors, and (2) the 6 correlations of the 6 predictors with behavior regularity in their individual data. The mean value of these correlations differed significantly from 0 in the predicted direction (t = 4.59, df = 114, p < .00001, one-tailed).
